# A Transferable Force Field for Simulating Adsorption in Metal–Organic Frameworks with Open Metal Sites Based on the 12–6–4 Lennard-Jones Potential

**DOI:** 10.1021/acs.jcim.5c02893

**Published:** 2026-01-24

**Authors:** Meng Du, Alan Rodriguez, Matthew Z. Lin, Haoyuan Chen

**Affiliations:** † Department of Chemistry, 2765 Southern Methodist University, Dallas, Texas 75275, United States; ‡ Department of Physics and Astronomy, 12331 The University of Texas Rio Grande Valley, Edinburg, Texas 78539, United States; ¶ 41618 South Texas ISD Science Academy, Mercedes, Texas 78570, United States

## Abstract

Metal–organic frameworks (MOFs) that contain coordinatively unsaturated open metal sites (OMSs) provide strong host–guest interactions, making them promising sorbents for low-concentration gas adsorption applications such as direct air capture and atmospheric water harvesting. However, accurately modeling host–guest interactions involving OMSs remains challenging for classical force fields (FFs) based on the 12–6 Lennard–Jones (LJ) potential, as the polarization effect of the guest molecule induced by the positively charged OMS is not considered. Here, we introduce an FF based on the 12–6–4 LJ potential, which incorporates charge–induced dipole interactions and is parametrized against a diverse set of host–guest potential energy surfaces (PESs) obtained from density functional theory (DFT). The resulting FF, trained on a generic trimetallic cluster, performs well in both host–guest binding energetics and gas adsorption isotherms across different OMS-containing MOFs, including MOF-74 series and Cu-BTC. These results highlight the excellent transferability of our approach and its potential to enhance the accuracy and robustness of high-throughput MOF discovery workflows, particularly for gas adsorption and separation in large and diverse MOF databases.

## Graphical abstract

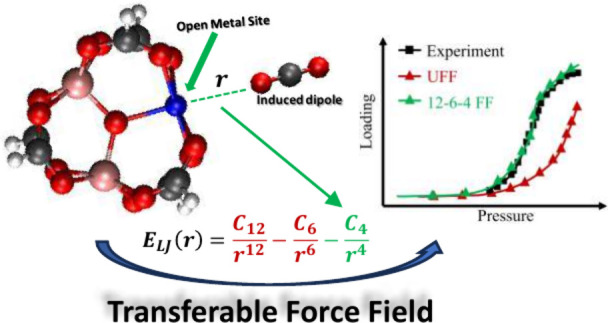

## Introduction

Metal–organic frameworks (MOFs) have emerged as a versatile class of nanoporous materials with applications spanning gas adsorption, separation, catalysis and others.^1–3^ For gas adsorption/separation, the unique advantages of MOFs are their high porosity and tunable host–guest chemistry. Coordinatively unsaturated open metal sites (OMSs), which are seen in many popular MOFs such as MIL-101,^4^ MOF-74 ^5^ and Cu-BTC,^6^ provide strong binding sites for guest molecules that enables enhanced uptake particularly at low concentrations.^7,8^ However, accurate modeling of adsorption in OMS-containing MOFs remains challenging, as the OMS-guest interactions involve polarization effects which are not included in the framework of conventional force fields (FFs).^9–12^ This limits the accuracy and efficiency of high-throughput computational MOF discovery for adsorption applications, since MOFs with OMSs constitute a significant fraction of commonly used databases.^13^

Despite the recent surge of machine learning interatomic potentials (MLIPs),^14–18^ classical FFs remain the “default” choice for grand-canonical Monte Carlo (GCMC) adsorption simulations due to their efficiency and interpretability. Generic FFs such as UFF^19^ and DREIDING^20^ are widely used in this field and have proven successful for many MOFs.^21–25^ However, they have been shown to yield inconsistent results in OMS-containing systems^26–29^ where polarization effects become non-negligible. Many efforts have been made to overcome this shortcoming. One could refit the FF parameters for a specific system (which might also involve using alternate functional forms for the FF, such as the Buckingham potential),^30–36^ but the transferability across different types of MOFs can be limited. For instance, the parameters fitted by Mercado et al.^33^ for Mg-MOF-74 produced significant deviations when applied to Mg_2_(dobdpc), a closely related analogue. Polarizable FFs have also been developed for GCMC adsorption simulations,^9,11,12,37–39^ but the computational cost can be significantly higher due to the need of iterative self-consistent calculations.

The essential physics of OMS–guest binding can be approximated as the interaction between a point charge on the OMS and the dipole induced on the guest molecule by the electric field of that charge.^40^ Back polarization (the electric field of the guest acting on the OMS) and higher-order terms can be neglected, since the positively charged OMS is less polarizable than the guest. This charge-induced dipole interaction follows a *r* ^–4^ dependence, where *r* is the interatomic distance, and can be incorporated into classical FFs by extending the standard 12–6 Lennard–Jones (LJ) potential to a 12–6–4 form. In this way, the essential interactions are captured while the simplicity of conventional FFs is retained, without requiring self-consistent iterations. This approach was pioneered by Li, Merz and co-workers for aqueous ions^41–43^ and has been widely applied in biomolecular simulations,^43–50^ and materials modeling.^51–53^ Recently, We have implemented the 12–6–4 FF in the widely used GCMC code RASPA^54^ and tested it on water adsorption in Mg-MOF-74.^55^ Without reparametrization, the 12–6–4 FF with the original parameters derived for aqueous Mg^2+^ ions was quite accurate in describing water binding on Mg-OMS and yielded a water adsorption isotherm that agreed much better with experiments than UFF did.^55^ Here, we present a systematic parametrization of the 12–6–4 FF for GCMC adsorption simulations. By having 60 metal-guest combinations from 12 common metals in MOFs and 5 representative guest molecules and fitting the coefficients of the *r* ^–4^ term against density functional theory (DFT)-derived potential energy surfaces (PESs), we obtained an FF that agrees well with DFT on OMS-guest binding energies and demonstrates good transferability across different types of OMS-containing MOFs in terms of both host–guest binding energetics and simulated gas adsorption isotherms. These findings demonstrate the potential of our approach in enhancing high-throughput MOF discovery workflows, particularly for adsorption and separation in large-scale MOF databases that contain significant amounts of OMS-containing MOFs.

## Methods

### Generation of PESs with DFT

To represent the typical local coordination environment of OMSs in MOFs, a trimetallic cluster (TMC) (Figure 1) as appeared in widely studied MOFs such as MIL-101 ^4^ and PCN-250 ^56^ was selected as the molecular model for FF parametrization. The chemical composition of TMC is M_3_O­(HCOO)_6_, where the sum of oxidation states for the three metal centers is +8 to ensure charge neutrality. This allows the incorporation of both +2 and +3 metals. Specifically, when the guest molecule-binding OMS was a + 2 metal (Mg­(II), Mn­(II), Fe­(II), Co­(II), Ni­(II), Cu­(II), or Zn­(II)), both of the two distal metal sites (spectators) were fixed as Al­(III). When the guest molecule-binding OMS was a + 3 metal (Mn­(III), Fe­(III), Co­(III), Al­(III), or Cr­(III)), the two distal ions were set as one Al­(III) and one Mg­(II). Closed-shell Al­(III) and Mg­(II) were chosen for distal metal sites to avoid potential complications in the total spin of the system.

**Figure 1:**
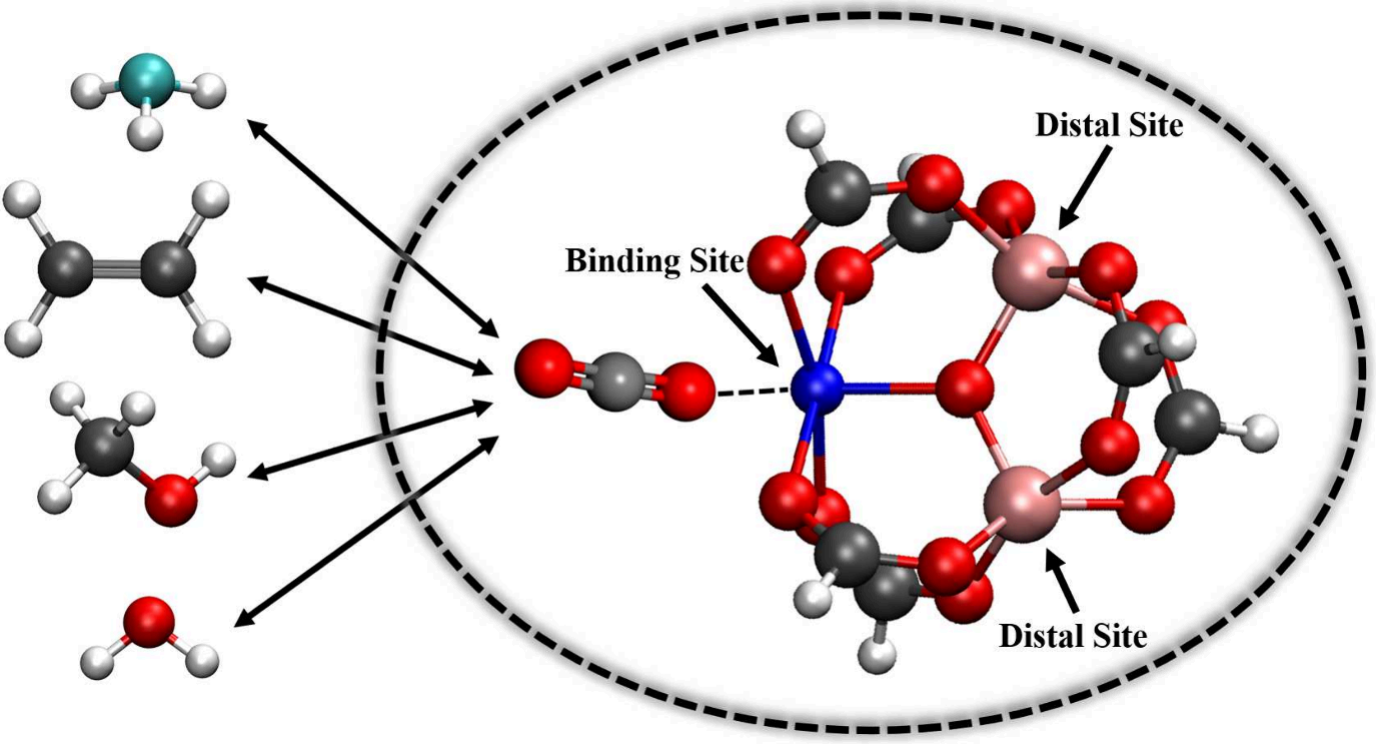
Structure of the TMC used for FF parametrization and the guest molecules considered. Left: guest molecules (NH_3_, C_2_H_4_, CH_3_OH, H_2_O, and CO_2_). Right: a representative DFT-optimized structure of CO_2_ binding at the open metal site (Cu^2+^) in the TMC, with the Al^3+^ centers located at distal positions. Color code: H–white, C–gray, N–cyan, O–red, Al–pink, Cu–blue.

For each of the 12 TMCs considered, its DFT-optimized geometry was combined with each of the five guest molecules (CO_2_, C_2_H_4_, CH_3_OH, H_2_O, and NH_3_) to generate a PES of TMC-guest binding energy as a function of OMS-guest distance *r*. The point on guest molecule used to calculate *r* from the OMS is the O atom for H_2_O and CH_3_OH, one of the O atoms for CO_2_, the N atom for NH_3_, and the center of two C atoms for C_2_H_4_. For all guest molecules except C_2_H_4_, the range of *r* was set to 1.8–2.7 Å with a 0.1 Å step size to ensure that the global minimum (often within 2.0–2.2 Å) was included. For C_2_H_4_, the range was adjusted to 2.1–3.0 Å, since the minimum often appear near 2.4–2.8 Å. All DFT calculations were carried out using Gaussian16 ^57^ at the M06-L^58^/def2-TZVP^59^ level of theory with Grimme’s DFT-D3 dispersion correction.^60^ This level of theory has been shown to perform reliably for related systems from extensive benchmarks.^61,62^ For each metal-guest pair, the binding energy was defined as

1 $$E_{\mathrm{binding}}^{\mathrm{DFT}}=E_{\mathrm{TMC}+\mathrm{guest}}^{\mathrm{DFT}}-E_{\mathrm{TMC}}^{\mathrm{DFT}}-E_{\mathrm{guest}}^{\mathrm{DFT}}$$

### Formulation of the 12–6–4 FF

The 12–6–4 LJ-based FF potential energy as a function of interatomic distance *r*_*ij*_ between atoms *i* and *j* has the following form:^41^

2 $$E_{ij}\left(r_{ij}\right)=4\varepsilon_{ij}\left[{\left(\frac{\sigma_{ij}}{r_{ij}}\right)}^{12}-{\left(\frac{\sigma_{ij}}{r_{ij}}\right)}^{6}\right]-\frac{C_{4}^{ij}}{r_{ij}^{4}}+\frac{q_{i}q_{j}}{4\pi \varepsilon_{0}r_{ij}}=\frac{C_{12}^{ij}}{r_{ij}^{12}}-\frac{C_{6}^{ij}}{r_{ij}^{6}}-\frac{C_{4}^{ij}}{r_{ij}^{4}}+\frac{q_{i}q_{j}}{4\pi \varepsilon_{0}r_{ij}}$$

in which the first, second and last terms on the r.h.s. are the same as in conventional FFs such as UFF and DREIDING. The third term on the r.h.s. describes the interaction between a point charge and an induced dipole, so the *C*_4_^*ij*^ parameter can be derived by

3 F(rij)=q4πε0εrrij2μind=αF(rij)=αq4πε0εrrij2Eijind(rij)=−12μindF(rij)=−12αF2(rij)=−12α(q4πε0εrrij2)2=−C4ijrij4C4ij=12α(q4πε0εr)2∝q2

where α is the polarizability of the guest, *q* is the charge on the metal center, *ε*_*r*_ is the relative dielectric constant (set to 1 in this work), *F*(*r*_*ij*_) is the electric field from the charge, and μ^*ind*^ is the induced dipole on the guest.^63^ This provides the physical meaning of the additional *r* ^–4^ term and suggests that the *C*_4_ parameter for the same metal in different chemical environments should be scaled by the square of the metal atom’s partial charge.

### Parametrization Workflow

The 12–6–4 FF was parametrized to fit the DFT-derived PESs of guest molecule binding on TMCs by minimizing the mean absolute error (MAE), in which *C*_12_, *C*_6_, and *C*_4_ were treated as adjustable parameters for each OMS-guest atom pair. The guest atom is defined as C_2_H_4_: both C atoms; CO_2_: both O atoms; CH_3_OH and H_2_O: O atom; NH_3_: N atom. For other interactions, the standard 12–6 LJ potential was used. The MOF atoms were described using DREIDING (nonmetal) or UFF (metal). C_2_H_4_ and CH_3_OH were modeled with the TraPPE united-atom (UA) model,^64,65^ while CO_2_ and NH_3_ were modeled with the TraPPE all-atom (AA) model.^66,67^ For H_2_O, five widely used rigid models—TIP3P,^68^ TIP4P,^68^ TIP4PEW,^69^ TIP5P,^70^ and TIP5PE^71^—were tested, and the fitted parameters were found to be insensitive to the water model (Figure S24 in the Supporting Information). For consistency, the TIP4PEW model was used throughout this work. Lorentz–Berthelot mixing rules were used to obtain the pairwise 12–6 LJ parameters. All FF energy calculations were performed using our code FFEnergy (https://github.com/haoyuanchen/FFEnergy). The total host–guest binding energy is thus expressed as

4 $$E_{\mathrm{binding}}=E_{\mathrm{LJ,}\mathrm{OMS‐guest}}^{12-6-4}+E_{\mathrm{LJ,}\mathrm{others}}^{12-6}+E_{\mathrm{Coulomb}}$$

Here, the 12–6–4 potential is applied exclusively to interactions between the open metal sites and guest molecules, while the conventional 12–6 potential is used for all remaining van der Waals interactions, and only *E*_LJ_^12–6–4^ was subject to parametrization. In the parameter optimization, the ranges for the parameters were defined as *C*_12_: 10^6^–10^9^ K·Å^12^, *C*_6_: 1–10^6^ K·Å^6^, and *C*_4_: 0–6 × *C*_6_ K·Å^4^, in which the ranges for *C*_12_ and *C*_6_ were chosen after surveying UFF, DREIDING, and TraPPE parameters, ensuring that the search space remains broad yet physically meaningful. Here, K is used as the energy unit following the common practice in FF parametrization and GCMC simulations. For easier interpretation in chemistry context, the optimized parameters reported in Table 1 have been converted from K to kJ/mol. The constraint that *C*_4_ cannot be larger than 6 × *C*_6_ was adapted from Li and Merz^41^ (more details are in the Supporting Information). Also, to avoid the fitting being skewed by a few outliers, all points in the PES with DFT binding energies higher than 12.5 kJ mol^–1^ (about 5 *k*_*B*_ *T* at room temperature) were excluded from fitting, as those high energy configurations were largely inaccessible in GCMC simulations. *E*_Coulomb_ was computed using CHELPG charges^72^ computed at the aforementioned M06-L-D3/def2-TZVP level.

**Table 1:** Optimized 12–6–4 FF Parameters for Selected Metal–Guest Atom Pairs

**Guest:** CO_2_		**Pair atom:** O	
Metal	*C*_12_ [kJ mol^–1^ Å^12^]	*C*_6_ [kJ mol^–1^ Å^6^]	*C*_4_ [kJ mol^–1^ Å^4^]
Co(II)	152308.13	70.12	353.10
Cu(II)	95673.97	64.04	314.20
Fe(II)	178415.69	88.00	436.39
Mg(II)	101511.42	47.53	230.66
Mn(II)	187882.73	68.44	319.76
Ni(II)	133158.78	66.35	359.28
Zn(II)	135940.44	46.19	206.20

**Guest:** H_2_O (TIP4PEW)		**Pair atom:** O	
Metal	*C*_12_ [kJ mol^–1^ Å^12^]	*C*_6_ [kJ mol^–1^ Å^6^]	*C*_4_ [kJ mol^–1^ Å^4^]
Co(II)	176286.42	89.41	463.69
Cu(II)	123387.66	117.08	557.00
Fe(II)	189131.45	88.90	459.27
Mg(II)	119953.26	18.57	67.48
Mn(II)	246820.13	56.10	285.75
Ni(II)	154263.33	109.63	565.11
Zn(II)	215673.86	64.25	318.61

The particle swarm optimization (PSO) algorithm was used to efficiently sample the high-dimensional parameter space.^73^ The hyperparameters were optimized to improve accuracy and efficiency while preventing overfitting (see the Supporting Information for details). The workflow in Figure 2 represents one complete PSO cycle. This procedure was independently repeated 10 times, and the final FF parameters for each metal–guest pair were determined as the representative closest to the geometric center among the 10 cycle representatives.

**Figure 2:**
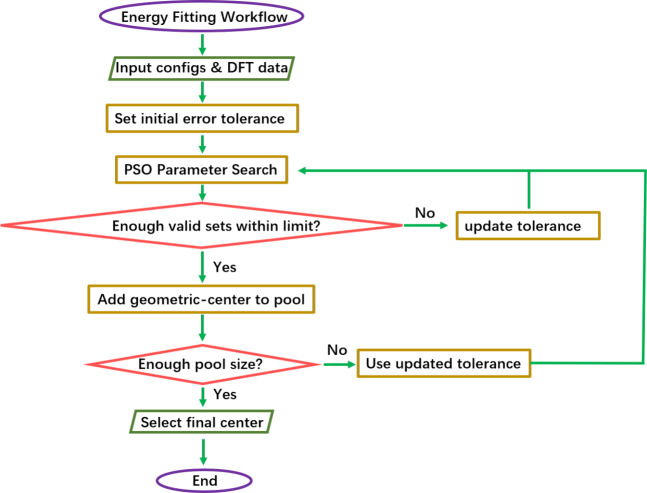
Flowchart of a single PSO parameter fitting run. The full parametrization strategy repeats this process 10 times, followed by geometric-center-based selection of the final representative.

### GCMC Simulations

Adsorption isotherms were simulated using GCMC in RASPA2.^54^ All MOF structures were taken directly from the QMOF database,^74^ where they had been optimized at the PBE-D3­(BJ) level^60,75^ and the partial atomic charges were assigned using the DDEC method.^76^ In all simulations, the frameworks were treated as rigid, thus eliminating interactions between MOF atoms. The host–guest interactions were modeled using the 12–6 DREIDING­(nonmetal)/UFF­(metal), and the 12–6–4 FF overrode the 12–6 FF only for OMS-guest atom pairs^55^ (https://github.com/haoyuanchen/RASPA-tools/tree/master/LJ1264Potential). Guest–guest interactions were modeled using TraPPE. The Peng–Robinson equation of state was used to describe the implicit bulk phase,^77^ with the critical temperature, critical pressure and acentric factor taken from Poling et al.^78^

For each state point in an adsorption isotherm, the GCMC simulation consisted of 100,000 production cycles after 10,000 initialization cycles. The number of MC moves in each cycle was max­(20, number of adsorbate molecules). Insertion, deletion, translation, rotation and reinsertion moves were all attempted with equal probability. A cutoff radius of 12.8 Å was applied, and long-range electrostatics was handled with Ewald summation with a precision of 10^–6^. Supercells were used to satisfy the minimum image convention: 4 × 2 × 2 for M-MOF-74; 2 × 2 × 2 for Cu-BTC.

## Results and Discussion

### Comparison of FF and DFT PESs

#### Parametrization: TMCs

As described in the Methods Section, *C*_12_, *C*_6_ and *C*_4_ parameters were obtained for a total of 60 metal-guest atom pairs in TMCs. The optimized parameters between CO_2_, H_2_O and all metals used for GCMC simulations later in this work are summarized in Table 1, while the full set of parameters is provided in the Supporting Information (Table S4). One representative 12–6–4 PES for each guest molecule is shown in Figure 3, alongside the DFT reference and DREIDING/UFF comparisons. The complete set of PESs is provided in the Supporting Information (Figures S2–S13). As seen in the figures, the parametrized 12–6–4 FF consistently led to much better agreement with DFT, compared to DREIDING/UFF with largely overestimated the energies. For the entire data set, it is also clear from Figure 4 that the 12–6–4 energies correlated much better with DFT energies. Compared to DREIDING/UFF, the MAEs were reduced from over 100 kJ mol^–1^ to less than 10 kJ mol^–1^. For H_2_O, we tested five water models (TIP3P, TIP4P, TIP4PEW, TIP5P, and TIP5PE) and no model dependence was observed (Figure S24). These results all showed that our parametrized 12–6–4 FF can precisely reproduce DFT PESs for OMS-guest binding, which is necessary for accurate GCMC simulations.

**Figure 3:**
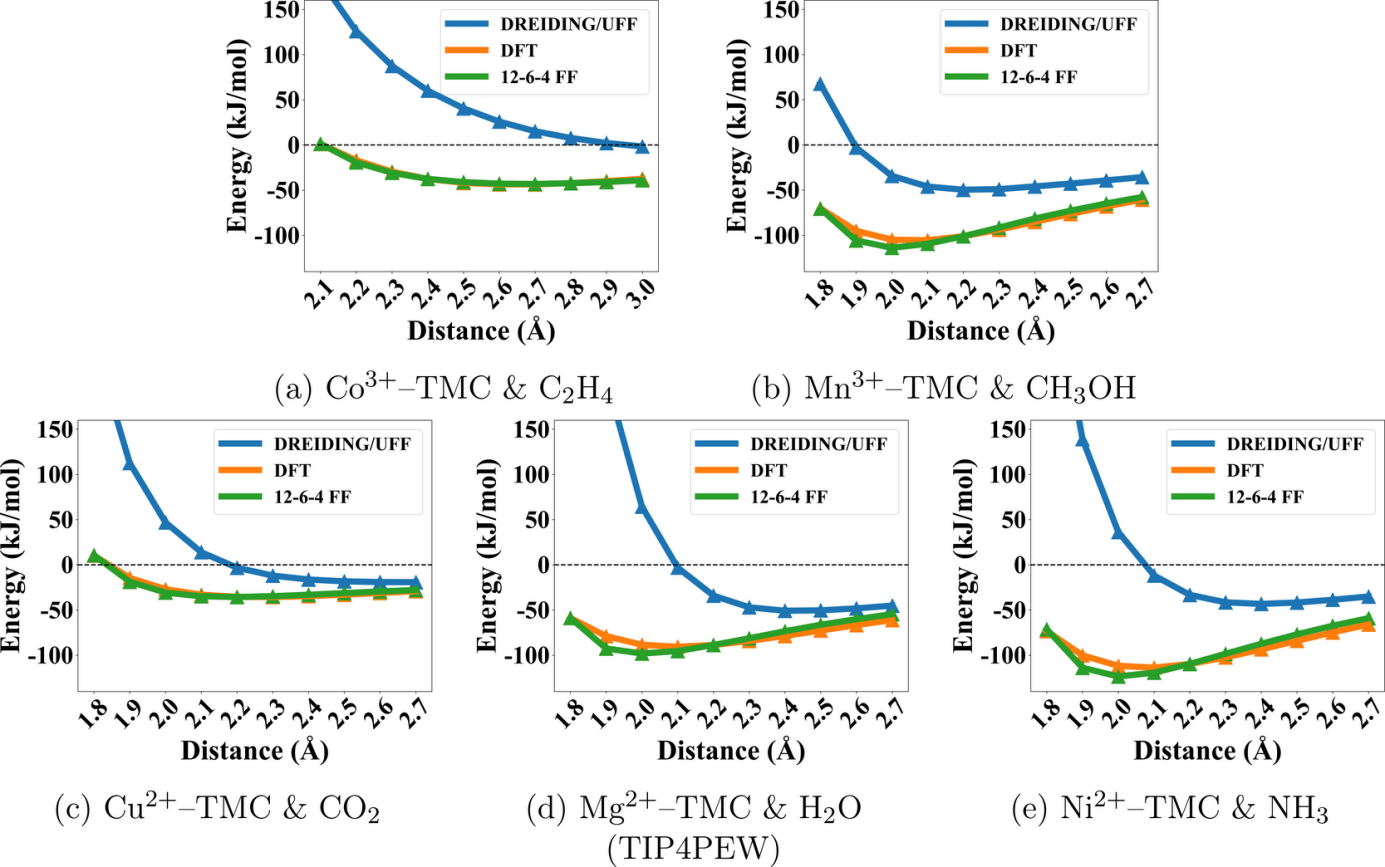
Representative potential energy surface (PES) comparisons for five TMC–guest systems as a function of the metal–guest distance. DFT reference interaction energies (orange) are compared with predictions from DREIDING/UFF (blue) and the fitted 12–6–4 force field (green). Panels correspond to (a) Co^3+^–TMC with C_2_H_4_, (b) Mn^3+^–TMC with CH_3_OH, (c) Cu^2+^–TMC with CO_2_, (d) Mg^2+^–TMC with H_2_O (TIP4PEW), and (e) Ni^2+^–TMC with NH_3_. The black dashed line indicates zero binding energy (0 kJ mol^–1^).

**Figure 4:**
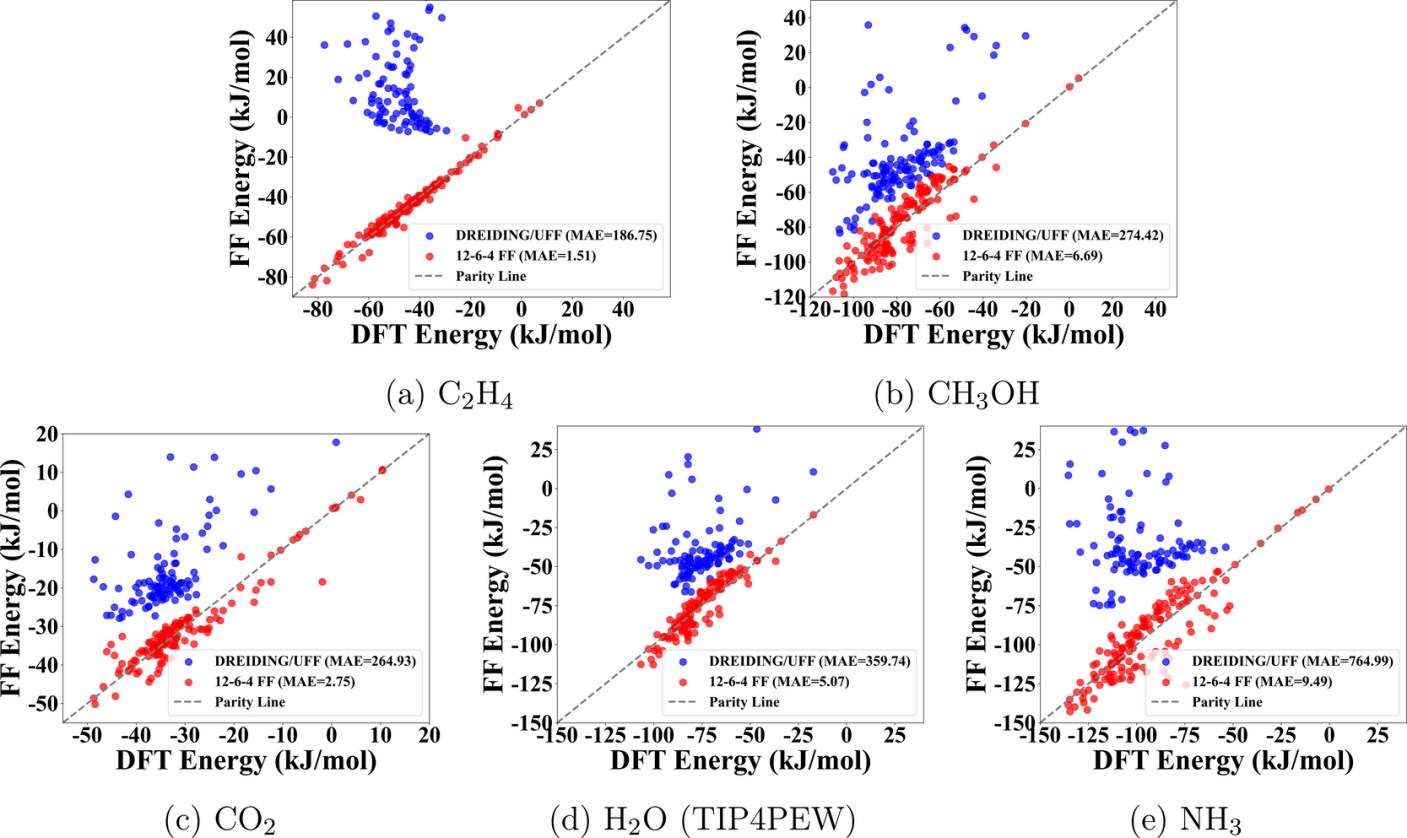
Correlation plots between FF (*Y*-axis) and DFT (*X*-axis) TMC-guest binding energies for all guest molecules: (a) C_2_H_4_, (b) CH_3_OH, (c) CO_2_, (d) H_2_O (TIP4PEW), and (e) NH_3_. Blue: DREIDING/UFF; red: parametrized 12–6–4 FF; black dashed line: parity line.

To further explore the parameter space, we also tested multiple alternative fitting protocols. These included 1) keeping the *C*_12_ and *C*_6_ parameters combined from DREIDING/UFF and TraPPE FFs, only fit *C*_4_; 2) make *C*_4_ proportional to *ε* of the guest atom (as *ε* reflects the polarizability α of the guest, which is proportional to *C*_4_, see eq 3). Both approaches reduce the parameter space, but both led to significantly higher MAEs (Figure S22). This suggested that refitting *C*_12_, *C*_6_, and *C*_4_ together is necessary, which is consistent with a previous work showing that the 3-parameter 12–6–4 potential is necessary to ensure the robustness of the FF, while the 2-parameter 12–6 potential could lead to overfitting.^79^

#### Transferability Test: MOF-74 and Cu-BTC

To test the transferability of our 12–6–4 FF parameters fitted to TMCs, we performed OMS-guest binding PES scan for two other types of popular OMS-containing MOFs: M-MOF-74 (M = Fe, Co, Cu, Mg, Mn, Ni, and Zn) and Cu-BTC, with the same five adsorbates NH_3_, C_2_H_4_, CH_3_OH, H_2_O, and CO_2_. In these DFT-referenced PES comparisons, the *C*_4_ term was consistently adjusted to reflect differences in the metal partial charges between the TMC training models and the extended MOF systems, including both the M-MOF-74 series and Cu-BTC. Specifically, metal charges obtained from CHELPG analyses were used to rescale the magnitude of the *C*_4_ contribution, ensuring a physically consistent treatment of charge–induced dipole interactions across different coordination environments. As seen from the correlation plots in Figure 5, the 12–6–4 FF still agreed much closer with DFT than DREIDING/UFF did, even though its parameters were not trained on these systems. All PESs for guest binding on Cu-BTC are shown in Figure 6, and all PESs for guest binding on M-MOF-74 are provided in the Supporting Information (Figures S14–S20). Again, the 12–6–4 PESs agreed very well with DFT, while DREIDING/UFF continued to overestimate the binding energies, particularly in the short-range. These results suggested that our 12–6–4 FF has excellent transferability and could significantly improve the reliability of GCMC simulation results for gas adsorption in OMS-containing MOFs.

**Figure 5:**
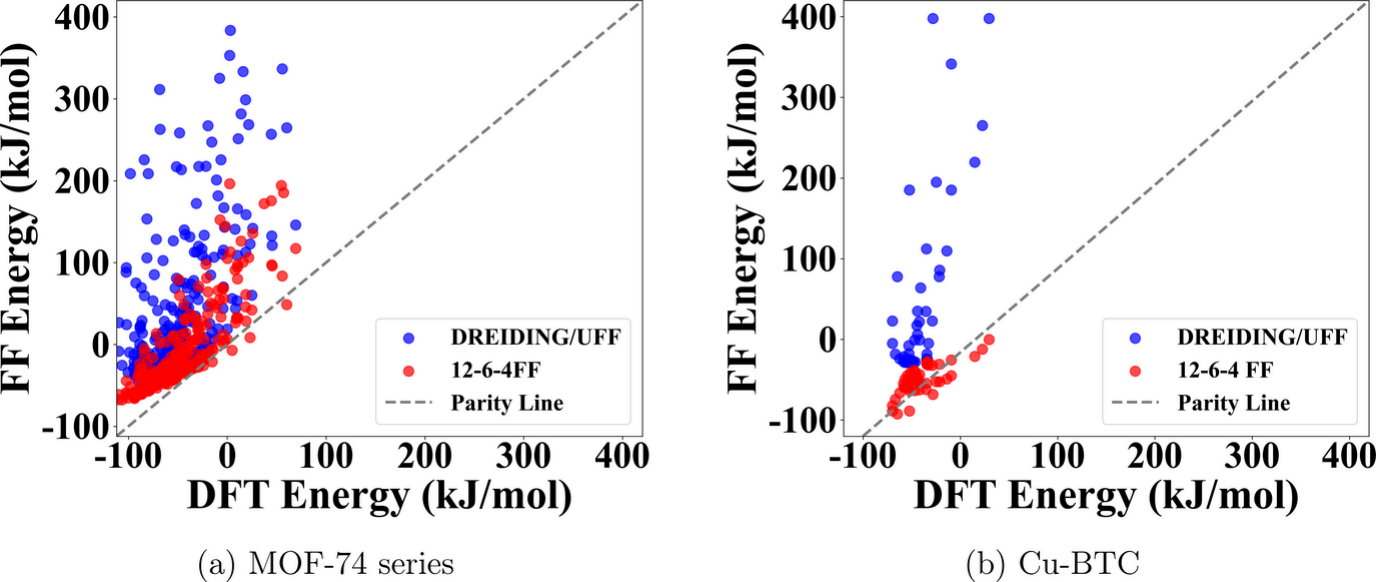
Correlation plots of classical versus DFT PES for MOF-74 series (a) and Cu-BTC (b). Each panel aggregates *all* metal–guest combinations for the five representative adsorbates­(C_2_H_4_, CH_3_OH, CO_2_, H_2_O (TIP4PEW), and NH_3_). Black dashed line: parity line. Blue: DREIDING/UFF; red: fitted 12–6–4.

**Figure 6:**
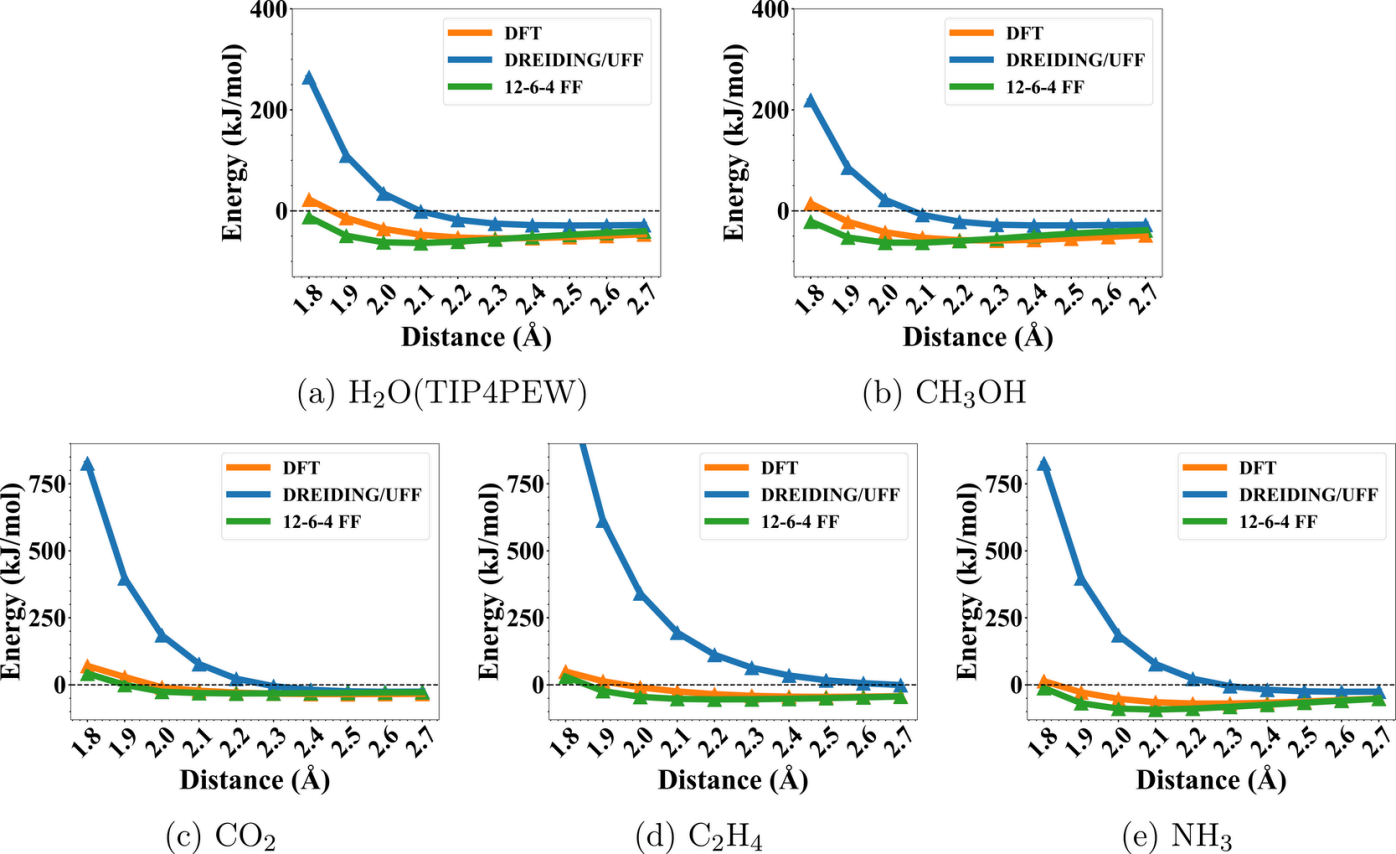
Potential energy surface (PES) comparisons for five Cu–BTC–guest systems as a function of the Cu–guest distance. DFT reference interaction energies (orange) are compared with predictions from DREIDING/UFF (blue) and the fitted 12–6–4 force field (green). Panels correspond to (a) H_2_O (TIP4PEW), (b) CH_3_OH, (c) CO_2_, (d) C_2_H_4_, and (e) NH_3_. The black dashed line indicates zero binding energy (0 kJ mol^–1^).

### GCMC Simulations of Gas Adsorption Isotherms

#### Charge-Dependent Scaling of *C*_4_

With the parametrized 12–6–4 FF showing excellent accuracy and transferability in terms of OMS-guest binding energies, we then tested it in GCMC simulations of gas adsorption in M-MOF-74 and Cu-BTC. To account for the difference in partial atomic charges of the same metal in different MOFs, the *C*_4_ parameter in the GCMC simulations was scaled by *C*_4_^*MOF*^ = *C*_4_^*TMC*^ × (*q*_*M*_^*MOF*^/*q*_*M*_^*TMC*^)^2^, where *q*_*M*_ is the partial charge of the metal atom, obtained from CHELPG (TMC) or DDEC (MOF). As mentioned above, this scaling method is based on the physical fact that *C*_4_ reflects charge-induced dipole interactions and is proportional to *q*_*M*_^2^ (eq 3). Details on partial atomic charges and scaling of *C*_4_ are provided in the Supporting Information (Tables S1–S3).

GCMC simulations were performed to simulate CO_2_ and H_2_O adsorption in M-MOF-74 (M = Fe, Co, Cu, Mg, Mn, Ni, and Zn) as well as Cu-BTC. These two adsorbates were selected because of the larger amounts of experimental and simulation data available in the literature. For CO_2_, simulations were conducted over pressures from 1 Pa to 4.0 MPa at multiple temperatures (278, 296, 298, 313, 343, 393, and 473 K for M-MOF-74; 295, 298, 323, 348, 373, and 378 K for Cu-BTC) to compare with other results. For H_2_O, isotherms were computed at 298 K up to 5000 Pa. To account for the difference between ideal crystal structures used in simulations and real materials used in experiments, all simulated isotherms were uniformly scaled by the ratio between experimental and theoretical pore volumes of each MOF. For Cu-BTC, the experimental pore volume was taken as 0.658 cm^3^ g^–1^ reported by Wang et al.,^80^ while the theoretical pore volume was 0.82 cm^3^ g^–1^ as calculated by Liu et al.,^81^ which was also consistent with the upper bound of a wide set of experimental data. For M-MOF-74, Queen et al. have shown that experimental pore volumes are typically around 15% smaller than theoretical values.^82^ Therefore, a uniform scaling factor of 0.85 was applied to all M-MOF-74 adsorption isotherms for simplicity.^9^ The main text here focuses on the adsorption of CO_2_ and H_2_O in Mg-MOF-74, Co-MOF-74, and Cu-BTC at 298 K, which are among the most widely studied systems. The complete set of simulated GCMC isotherms, along with comparisons with other experimental and simulated data in the literature, are provided in Figures S25–S68 of the Supporting Information.

#### MOF-74

The simulated adsorption isotherms of H_2_O and CO_2_ in Co- and Mg-MOF-74 as well as previously reported experimental/simulated results are summarized in Figure 7. For H_2_O in Co-MOF-74 (Figure 7a), the experimental reference from Glover et al. shows a sharp step around 200 Pa, with saturation near 28 mol kg^–1^.^83^ Mercado et al. trained a FF specifically on Co-MOF-74, but it predicted a step that occurs at much lower pressure.^33^ The step predicted by our 12–6–4 FF trained on TMCs was at a slightly higher pressure but was closer to the experimental value than the step from Mercado’s simulations. For H_2_O in Mg-MOF-74 (Figure 7b), multiple sets of experimental results exhibited noticeable variation on both the saturation loading and the step location,^83–85^ as water adsorption in MOFs is intrinsically difficult to measure experimentally due to several factors including incomplete activation, insufficient equilibration, and residual moisture. The simulated results from Mercado^33^ again predicted a step at a much lower pressure, while our 12–6–4 FF results aligned well with the consensus/average of multiple experimental results, particularly with Yang’s Mg_C data.^85^

**Figure 7:**
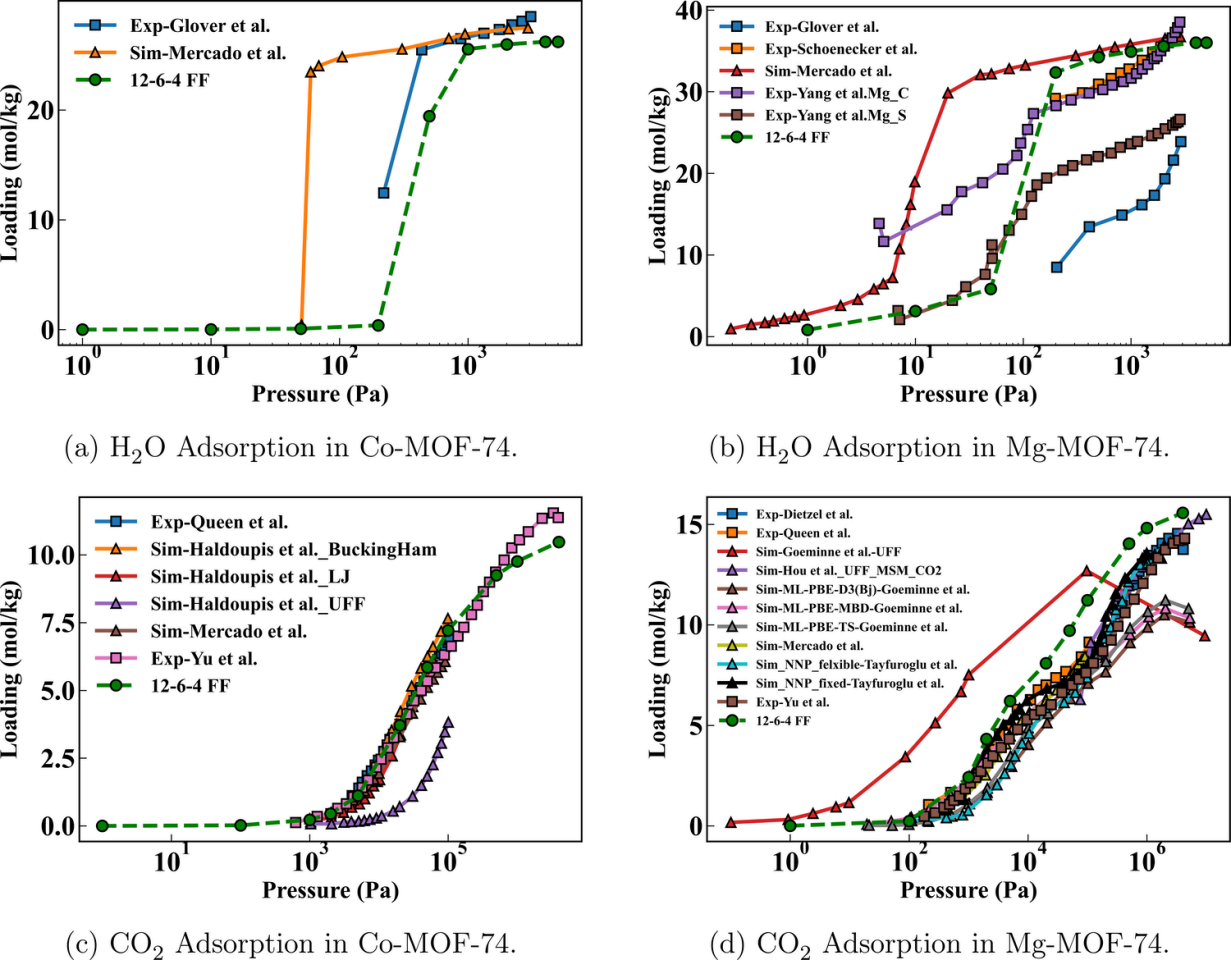
Comparison of experimental and simulated adsorption isotherms of H_2_O and CO_2_ in Co- and Mg-MOF-74 at 298 K. Panels correspond to (a) H_2_O adsorption in Co-MOF-74, (b) H_2_O adsorption in Mg-MOF-74, (c) CO_2_ adsorption in Co-MOF-74, and (d) CO_2_ adsorption in Mg-MOF-74. Experimental data from the literature are shown as filled squares, while simulation results reported in previous studies are shown as filled upward triangles. Results obtained from the present 12–6–4 force field are shown as filled green circles connected by green dashed lines.

For CO_2_ in Co-MOF-74 (Figure 7c), all experimental^86,87^ and simulated^33,88^ isotherms almost overlap, except for the one simulated using UFF. However, both Haldoupis^88^ and Mercado^33^ trained their FF specifically on Co-MOF-74, while our 12–6–4 FF was trained on TMCs. A similar situation was observed for CO_2_ in Mg-MOF-74 (Figure 7d), where the only outlier among all experimental^86,87,89^ and simulated^33,90–92^ isotherms was the one simulated using UFF. The agreement between (12–6–4) simulated and experimental isotherms for CO_2_ is even better compared to H_2_O, likely because strong hydrogen bonding and cooperative adsorption effects make H_2_O uptake more sensitive to subtle variations in the pore environment and host–guest interaction strength. The accuracy of our 12–6–4 FF was comparable with MLIPs,^90,92^ which were significantly slower and required much more data for training.

#### Cu-BTC

The simulated adsorption isotherms of H_2_O and CO_2_ in Cu-BTC as well as previously reported experimental/simulated results are summarized in Figure 8. For H_2_O adsorption (Figure 8a), our simulated isotherm overall agreed well with experiments,^84,85,93^ despite having a slightly higher pressure for the step. For CO_2_ adsorption (Figure 8b), all experimental^93–96^ and simulated^96^ isotherms including ours are closely aligned. The simulation by Yazaydin et al.^96^ using DREIDING/UFF slightly underestimated the adsorption, particularly at low to intermediate pressures.

**Figure 8:**
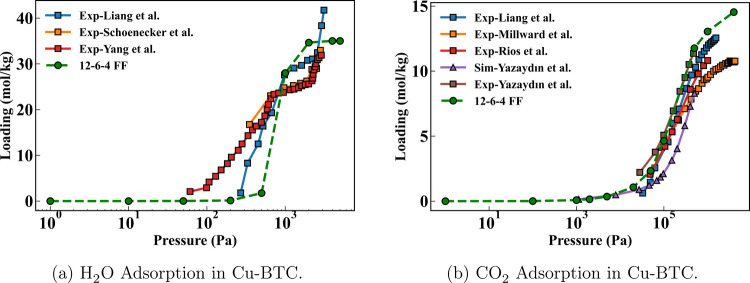
Comparison of experimental and simulated adsorption isotherms of H_2_O and CO_2_ in Cu-BTC at 298 K. Panels correspond to (a) H_2_O adsorption in Cu-BTC and (b) CO_2_ adsorption in Cu-BTC. Experimental data from the literature are shown as filled squares, while simulation results reported in previous studies are shown as filled upward triangles. Results obtained from the present 12–6–4 force field are shown as filled green circles connected by green dashed lines.

Overall, the GCMC results confirmed that our 12–6–4 FF is an accurate and transferable FF for the simulation of gas adsorption in OMS-containing MOFs.

## Conclusion

In this work, we developed an accurate and transferable force field based on the 12–6–4 Lennard-Jones potential for simulating gas adsorption in MOFs with open metal sites. Parametrized against DFT-derived potential energy surfaces of 60 metal-guest pairs (12 metals × 5 guest molecules) in a generic trimetallic cluster model, our force field explicitly incorporates a *r* ^–4^ polarization term to capture charge–induced dipole interactions which is absent in conventional force fields. The excellent accuracy and transferability of our force field is shown in the validation against other MOFs that also contain open metal sites, namely MOF-74 series and Cu-BTC. In terms of both host–guest binding potential energy surfaces and gas adsorption isotherms, our force field leads to better agreement with DFT and experimental data than not only conventional force fields like DREIDING and UFF but also some force fields specifically parametrized for those MOFs. This demonstrates the potential of our approach in improving the robustness of high-throughput computational screening over large and diverse MOF databases for adsorption and separation applications, as it does not require system-specific tuning of parameters and is much less computationally demanding than self-consistent polarizable force fields. Future work in our group aims to address the limitations of the current method–most notably the lack of universal mixing rules and the resulting reliance on explicitly fitted metal–guest interaction pairs—through more fundamental studies of polarization and electrostatics in host–guest binding.

## Supplementary Material





## Data Availability

The modified RASPA/RASPA2 force-field scripts used in this study are available at https://github.com/haoyuanchen/RASPA-tools/tree/master/LJ1264Potential. The FFEnergy package for classical force-field binding-energy calculations, together with the PSO optimization codes, can be accessed at https://github.com/haoyuanchen/FFEnergy. All representative input files and structural models employed in this work are provided in the Supporting Information.

## Notes

All fitted 12–6–4 force field parameters and all CHELPG/DDEC partial charges, potential energy surfaces for all five adsorbates binding on all TMC clusters, M-MOF-74 clusters, and Cu-BTC cluster, comparison of different parametrization schemes, CO_2_ and H_2_O adsorption isotherms in all MOFs considered in this work, sample Gaussian and RASPA input files (PDF)
Cartesian coordinates of cluster models and crystallographic information files of periodic structures (ZIP)
