# Supplementary material for: A Transferable Force Field for Simulating Adsorption in Metal–Organic Frameworks with Open Metal Sites Based on the 12–6–4 Lennard-Jones Potential
Source: J Chem Inf Model. 2026 Jan 24;66(3):1704–14. doi: 10.1021/acs.jcim.5c02893 (PMC12900520; doi:10.1021/acs.jcim.5c02893)
Supplement: Supplementary file 1 [file ci5c02893_si_001.pdf]

Supporting Information for: A Transferable Force Field for Simulating Adsorption in Metal-Organic Frameworks with Open Metal Sites Based on the 12-6-4 Lennard-Jones Potential

Meng Du,[†] Alan Rodriguez,[‡] Matthew Z. Lin,[¶] and Haoyuan Chen[*,†,‡]

†*Department of Chemistry, Southern Methodist University, Dallas, Texas 75275, USA*

‡*Department of Physics and Astronomy, The University of Texas Rio Grande Valley, Edinburg, Texas 78539, USA*

¶*South Texas ISD Science Academy, Mercedes, Texas 78570, USA*

E-mail: haoyuan@smu.edu

Contents

Contents

Partial Atomic Charges and Force Field Parameters **S4**

CHELPG Charges of TMC Clusters S4

CHELPG Charges of MOF-74 and Cu-BTC Clusters S4

DDEC Charges of Periodic Frameworks S4

Optimized 12-6-4 Parameters for Metal-Guest Pairs S5

Force Field Parameters of Atoms in Guest Molecules S9

Force Field Parameters of Atoms in MOFs S9

Parametrization Workflow **S12**

Underlying Theory S12

Code Implementation S13

Potential Energy Surfaces **S15**

Al(III)-TMC S15

Mn(III)-TMC S15

Cr(III)-TMC S17

Fe(III)-TMC S18

Co(III)-TMC S19

Co(II)-TMC S20

Cu(II)-TMC S21

Fe(II)-TMC S22

Mg(II)-TMC S23

Mn(II)-TMC S24

Ni(II)-TMC S25

Zn(II)-TMC S26

Co-MOF-74 . . . . S27
Cu-MOF-74 . . . . S28
Fe-MOF-74 . . . . S29
Mg-MOF-74 . . . . S30
Mn-MOF-74 . . . . S31
Ni-MOF-74 . . . . S32
Zn-MOF-74 . . . . S33
Cu-BTC S34

Comparison of Parametrization Schemes **S35**
Parameter Space S35
Search Range of κ S36
Choice of Water Model S37

Adsorption Isotherms **S38**
CO_2 adsorption in M-MOF-74 . . . . S38
H_2O adsorption in M-MOF-74 . . . . S53
CO_2 adsorption in Cu-BTC S56
H_2O adsorption in Cu-BTC S59

Example Input Files **S61**
Tri-Metal Cluster (TMC) Prototype: $MgAl_2$ S61
Mg-MOF-74 with CO_2 (Representative PES Scan) S62
Cu-BTC with CO_2 (Representative PES Scan) S65
GCMC S69

Partial Atomic Charges and Force Field Parameters

This section provides all partial atomic charges and force field parameters used in this work, including CHELPG[1] charges of TMC clusters at the M06-L-D3/def2-TZVP[2–4] level of theory, DDEC charges of periodic frameworks at the PBE-D3(BJ) level[4–6] (from QMOF database[7]), fitted 12-6-4 parameters for metal-guest pairs, and Lennard-Jones parameters for both guest molecules and MOF frameworks.

CHELPG Charges of TMC Clusters

Table S1: CHELPG charges of metal atoms in TMC clusters.

Metal	Mg(II)	Mn(II)	Fe(II)	Co(II)	Ni(II)	Cu(II)
Charge (e)	1.7156	1.3652	1.4359	1.3883	1.3701	1.2103
Metal	Zn(II)	Mn(III)	Fe(III)	Co(III)	Al(III)	Cr(III)
Charge (e)	1.4332	1.4947	1.6961	1.6395	2.0858	1.7422

—

CHELPG Charges of MOF-74 and Cu-BTC Clusters

Table S2: CHELPG Charges of metal atoms in MOF-74 and Cu-BTC clusters.

System	Mg-MOF74	Mn-MOF74	Fe-MOF74	Co-MOF74
Charge (e)	1.4326	1.0212	1.0127	0.9266
System	Ni-MOF74	Cu-MOF74	Zn-MOF74	Cu-BTC
Charge (e)	1.0835	0.7304	1.0690	1.0188

—

DDEC Charges of Periodic Frameworks

—

Table S3: Average DDEC charges of metal atoms in periodic MOF-74 and Cu-BTC frameworks.

System	Mg-MOF74	Mn-MOF74	Fe-MOF74	Co-MOF74
Charge (e)	1.4069	1.0180	0.9070	0.9435
System	Ni-MOF74	Cu-MOF74	Zn-MOF74	Cu-BTC
Charge (e)	0.9140	0.8700	0.9730	0.8360

Optimized 12-6-4 Parameters for Metal-Guest Pairs

Table S4: Optimized 12-6-4 parameters for all metal–guest pairs. Units: C_{12} (K·Å^{12}), C_6 (K·Å^6), C_4 (K·Å^4). Energy parameters expressed in K can be directly converted to kJ/mol using $E(\text{kJ/mol}) = R \times E(\text{K})$.

Metal	Guest Atom	C_{12}	C_6	C_4
Cu^{2+}	C_C2H4	20618801	38319	170269
Ni^{2+}	C_C2H4	46916620	395898	134562
Zn^{2+}	C_C2H4	44003159	30985	160674
Co^{2+}	C_C2H4	48381872	638602	65612
Co^{3+}	C_C2H4	79948003	647475	140365
Fe^{2+}	C_C2H4	20686371	34362	174627
Mg^{2+}	C_C2H4	62732550	28836	162858
Mn^{2+}	C_C2H4	86547535	33986	183560
Mn^{3+}	C_C2H4	67743347	682679	113507
Al^{3+}	C_C2H4	104429996	2014608	314
Fe^{3+}	C_C2H4	86643964	342636	176285
Cr^{3+}	C_C2H4	60169379	547409	181933
Cu^{2+}	O_CH3OH	14407422	18167	85841
Ni^{2+}	O_CH3OH	19985166	19214	97202
Zn^{2+}	O_CH3OH	19134747	13358	67244
Co^{2+}	O_CH3OH	20166892	17434	84093

Metal	Guest Atom	C_{12}	C_6	C_4
Co^{3+}	O_CH3OH	23617817	22657	114311
Fe^{2+}	O_CH3OH	23019243	18045	91781
Mg^{2+}	O_CH3OH	17350947	7984	42244
Mn^{2+}	O_CH3OH	24517577	11836	60904
Mn^{3+}	O_CH3OH	21496460	28498	139440
Al^{3+}	O_CH3OH	10466982	49906	227356
Fe^{3+}	O_CH3OH	25561925	19389	95987
Cr^{3+}	O_CH3OH	26441521	26623	149387
Cu^{2+}	O_H2O_TIP3P	16996278	18195	94382
Ni^{2+}	O_H2O_TIP3P	20688631	18031	90704
Zn^{2+}	O_H2O_TIP3P	28579558	13097	68206
Co^{2+}	O_H2O_TIP3P	23243693	16005	80744
Co^{3+}	O_H2O_TIP3P	33223582	22241	123106
Fe^{2+}	O_H2O_TIP3P	25058751	14858	79737
Mg^{2+}	O_H2O_TIP3P	18962795	8078	37621
Mn^{2+}	O_H2O_TIP3P	32651585	11947	61170
Mn^{3+}	O_H2O_TIP3P	21836946	25318	125074
Al^{3+}	O_H2O_TIP3P	17559174	24055	113815
Fe^{3+}	O_H2O_TIP3P	35764968	20077	107695
Cr^{3+}	O_H2O_TIP3P	27156696	27415	140327
Mg^{2+}	O_H2O_TIP4P	14158541	3431	17126
Zn^{2+}	O_H2O_TIP4P	25849335	8941	45409
Mn^{2+}	O_H2O_TIP4P	29273959	8693	42979
Fe^{2+}	O_H2O_TIP4P	22686783	12500	63335
Ni^{2+}	O_H2O_TIP4P	18401100	13852	73632

Metal	Guest Atom	C_{12}	C_6	C_4
Co^{2+}	O_H2O_TIP4P	21051364	12770	62435
Cr^{3+}	O_H2O_TIP4P	24669321	25096	123230
Al^{3+}	O_H2O_TIP4P	15100555	20345	97543
Cu^{2+}	O_H2O_TIP4P	14904009	15082	73473
Mn^{3+}	O_H2O_TIP4P	20015395	21433	110839
Fe^{3+}	O_H2O_TIP4P	32019972	16013	87082
Co^{3+}	O_H2O_TIP4P	30257863	19851	100528
Mg^{2+}	O_H2O_TIP4PEW	14427061	2234	8116
Mn^{2+}	O_H2O_TIP4PEW	29685638	6747	34368
Co^{2+}	O_H2O_TIP4PEW	21202383	10754	55769
Zn^{2+}	O_H2O_TIP4PEW	25939603	7728	38320
Cu^{2+}	O_H2O_TIP4PEW	14840124	14082	66992
Fe^{3+}	O_H2O_TIP4PEW	32116059	15975	79737
Fe^{2+}	O_H2O_TIP4PEW	22747285	10692	55238
Cr^{3+}	O_H2O_TIP4PEW	24390597	24588	116243
Co^{3+}	O_H2O_TIP4PEW	30355600	19326	94084
Mn^{3+}	O_H2O_TIP4PEW	20048497	18933	104422
Al^{3+}	O_H2O_TIP4PEW	15006035	17093	91357
Ni^{2+}	O_H2O_TIP4PEW	18553614	13185	67967
Al^{3+}	O_H2O_TIP5P	21934420	85280	321297
Fe^{3+}	O_H2O_TIP5P	38389922	71405	252839
Cr^{3+}	O_H2O_TIP5P	27213864	76715	307243
Mn^{3+}	O_H2O_TIP5P	24898446	104459	264518
Co^{3+}	O_H2O_TIP5P	31850633	75218	252424
Mg^{2+}	O_H2O_TIP5P	14801076	38622	199743

Metal	Guest Atom	C_{12}	C_6	C_4
Cu^{2+}	O_H2O_TIP5P	17340615	46820	199225
Fe^{2+}	O_H2O_TIP5P	25464184	56492	233031
Ni^{2+}	O_H2O_TIP5P	18618434	37821	201127
Co^{2+}	O_H2O_TIP5P	23707517	38873	206696
Zn^{2+}	O_H2O_TIP5P	29668460	40758	194949
Mn^{2+}	O_H2O_TIP5P	26612715	35328	182581
Al^{3+}	O_H2O_TIP5PE	22031992	139850	309576
Cr^{3+}	O_H2O_TIP5PE	28867502	151970	297646
Mn^{3+}	O_H2O_TIP5PE	23422529	70916	268898
Cu^{2+}	O_H2O_TIP5PE	15909872	38756	200643
Co^{3+}	O_H2O_TIP5PE	31866981	58076	252284
Mg^{2+}	O_H2O_TIP5PE	15328016	41789	197130
Fe^{3+}	O_H2O_TIP5PE	35579837	85832	255013
Co^{2+}	O_H2O_TIP5PE	23059161	45044	202367
Fe^{2+}	O_H2O_TIP5PE	25679425	48597	231829
Ni^{2+}	O_H2O_TIP5PE	18577798	36898	201894
Zn^{2+}	O_H2O_TIP5PE	29673682	36506	192477
Mn^{2+}	O_H2O_TIP5PE	25603202	34855	178698
Cu^{2+}	O_CO2	11506934	7702	37790
Ni^{2+}	O_CO2	16015320	7980	43212
Zn^{2+}	O_CO2	16349877	5555	24800
Co^{2+}	O_CO2	18318458	8433	42468
Co^{3+}	O_CO2	19465222	10060	49846
Fe^{2+}	O_CO2	21458475	10584	52486
Mg^{2+}	O_CO2	12209018	5716	27742

Metal	Guest Atom	C_{12}	C_6	C_4
Mn^{2+}	O_CO2	22597098	8231	38458
Mn^{3+}	O_CO2	15186043	8534	40537
Al^{3+}	O_CO2	8366259	124009	57539
Fe^{3+}	O_CO2	22684336	9654	47668
Cr^{3+}	O_CO2	22460972	16031	85485
Cu^{2+}	N_NH3	23147494	35584	188199
Ni^{2+}	N_NH3	24298258	26902	143318
Zn^{2+}	N_NH3	28442711	23082	115609
Co^{2+}	N_NH3	26992697	24928	129938
Co^{3+}	N_NH3	30059578	37165	177659
Fe^{2+}	N_NH3	29180513	23541	122214
Mg^{2+}	N_NH3	23380887	9465	46628
Mn^{2+}	N_NH3	33299012	18265	89684
Mn^{3+}	N_NH3	29369804	55564	231175
Al^{3+}	N_NH3	20867548	44615	198802
Fe^{3+}	N_NH3	30969207	26859	136359
Cr^{3+}	N_NH3	29654688	47043	207390

—

Force Field Parameters of Atoms in Guest Molecules

—

Force Field Parameters of Atoms in MOFs

Table S5: Lennard-Jones parameters and partial atomic charges of atoms in guest molecules used in this work.[8,8–15]

Atom Type	ε (K)	σ (Å)	q (e)
OwH2O_TIP3P	76.53	3.151	-0.834
HwH2O_TIP3P	0.0	1.0	0.417
OwH2O_TIP4P	78.0	3.154	0.0
HwH2O_TIP4P	0.0	1.0	0.52
M_H2O_TIP4P	0.0	1.0	-1.04
OwH2O_TIP4PEW	81.9	3.164	0.0
HwH2O_TIP4PEW	0.0	1.0	0.52422
M_H2O_TIP4PEW	0.0	1.0	-1.04844
OwH2O_TIP5P	80.52	3.12	0.0
HwH2O_TIP5P	0.0	1.0	0.241
M_H2O_TIP5P	0.0	1.0	-0.241
OwH2O_TIP5PE	89.57	3.097	0.0
HwH2O_TIP5PE	0.0	1.0	0.241
M_H2O_TIP5PE	0.0	1.0	-0.241
N_NH3	185.0	3.420	0.0
H_NH3	0.0	0.0	0.410
M_NH3	0.0	0.0	-1.230
C_CO2	27.0	2.80	0.7
O_CO2	79.0	3.05	-0.35
H_ua	0.0	0.0	0.265
O_roh	93.0	3.02	-0.700
H_roh	0.0	0.0	0.435
C_sp3	98.0	3.75	0.265
C_C2H4	85.0	3.675	0.0
H_C2H4	0.0	0.0	0.0

Table S6: Force field (DREIDING/UFF[16,17]) parameters of atoms in MOFs used in this work.

Atom	ε (K)	σ (Å)
H	7.64893	2.84642
C	47.8562	3.47299
O	48.1581	3.03315
Al	155.998	3.91105
Co	7.04507	2.55866
Cr	7.54829	2.69319
Cu	2.5161	3.11369
Fe	6.54185	2.5943
Mg	55.8574	2.69141
Mn	6.54185	2.63795
Ni	7.54829	2.52481
Zn	62.3992	2.46155

Parametrization Workflow

Underlying Theory

The functional form of the 12-6-4 Lennard-Jones potential and the physical meaning of the C_4 parameter have been discussed in the main text. In addition, the C_6 parameter can be estimated by the empirical formula:[18,19]

$$C_{6,ij} = \frac{3}{2}\,\frac{I_i I_j}{I_i + I_j}\,\alpha_i \alpha_j, \tag{1}$$

where α and I denote the polarizability and ionization energy, respectively. Combining Eq. 1 with the definition $C_4^{ij} = \kappa_{ij} C_6^{ij}$, where κ_{ij} was originally introduced by Li and Merz[20] to relate the polarization and dispersion contributions, yields

$$\kappa_{ij} \propto \frac{q_j^2 (I_i + I_j)}{\alpha_j I_i I_j}. \tag{2}$$

This expression indicates that κ_{ij} depends primarily on the metal–ion charge q_j, its polarizability α_j, and the ionization energies I_i and I_j of the interacting species. Since ionization energies vary only modestly among small guest molecules (H_2O: 12.6 eV; C_2H_4: 10.52 eV; CH_3OH: 10.85 eV; NH_3: 10.15 eV; CO_2: 13.77 eV[21–23]), their contributions can be approximated as constant. Therefore, κ is mainly governed by the intrinsic electronic properties of the metal ion, and its value is expected to be nearly independent of the ligand type. To avoid unphysical parameter values, we limited κ within the range of 0–6 Å^{-2}, as suggested by Li and Merz.[20] Notably, testing a narrower range of 0–3 Å^{-2} yielded fitted parameters whose resulting MOF-74 interaction energies differed by only ∼1.6% in MAE compared to those obtained using the broader 0–6 Å^{-2} range, confirming the robustness of the fitting (see Figure S23 for the energy correlation comparison). The final search ranges for the parameters were defined as: C_{12}: 10^6–10^9 K·Å^{12}, C_6: 1–10^6 K·Å^6, and C_4: 0–6 × C_6 K·Å^4, in which the ranges for C_{12} and C_6 were chosen after surveying UFF, DREIDING, and TraPPE

parameters, ensuring that the search space remains broad yet physically meaningful.

Implementation and Optimization Workflow

We used the FFEnergy package(`https://github.com/haoyuanchen/FFEnergy`) that we developed for energy calculations and parameter space explorations in batch. For a given host-guest system, the tool can compute the host-guest binding energy using different types of force fields with adjustable parameters and customizable options such as mixing rules and cutoffs, enabling systematic comparison with and parametrization against DFT data. This is integrated with the particle swarm optimization (PSO) method to construct the complete parametrization workflow. Each PSO run has 500 particles evolved for 500 iterations, with inertia weights and social/cognitive coefficients gradually adjusted to balance exploration and exploitation. To enhance sampling diversity, 20% of particles were randomly reinitialized every 50 steps. The candidate selection and filtering strategy is summarized in Fig. S1, where an 80% convergence threshold was introduced as a robustness measure. This criterion prevents premature termination of the optimization process and ensures that the swarm performs sufficiently extensive exploration before convergence. At the end of each run, the geometric center of all accepted solutions was computed, and the candidate nearest to this center was designated as the representative of that run. The workflow in Fig. S1 represents one complete PSO cycle. This procedure was independently repeated 10 times, and the final FF parameters for each metal–guest pair were determined as the representative closest to the geometric center among the 10 cycle representatives. This large-scale parameter search, combined with geometric-center-based representative selection, enhances the robustness of the fitting procedure by mitigating the risk of the error function becoming trapped in local minima and by reducing sensitivity to stochastic fluctuations. Several preliminary trial runs confirmed that the resulting parameter sets were highly consistent across independent cycles, demonstrating that the approach effectively suppresses the non-uniqueness of solutions that often arises in high-dimensional parameter fitting.

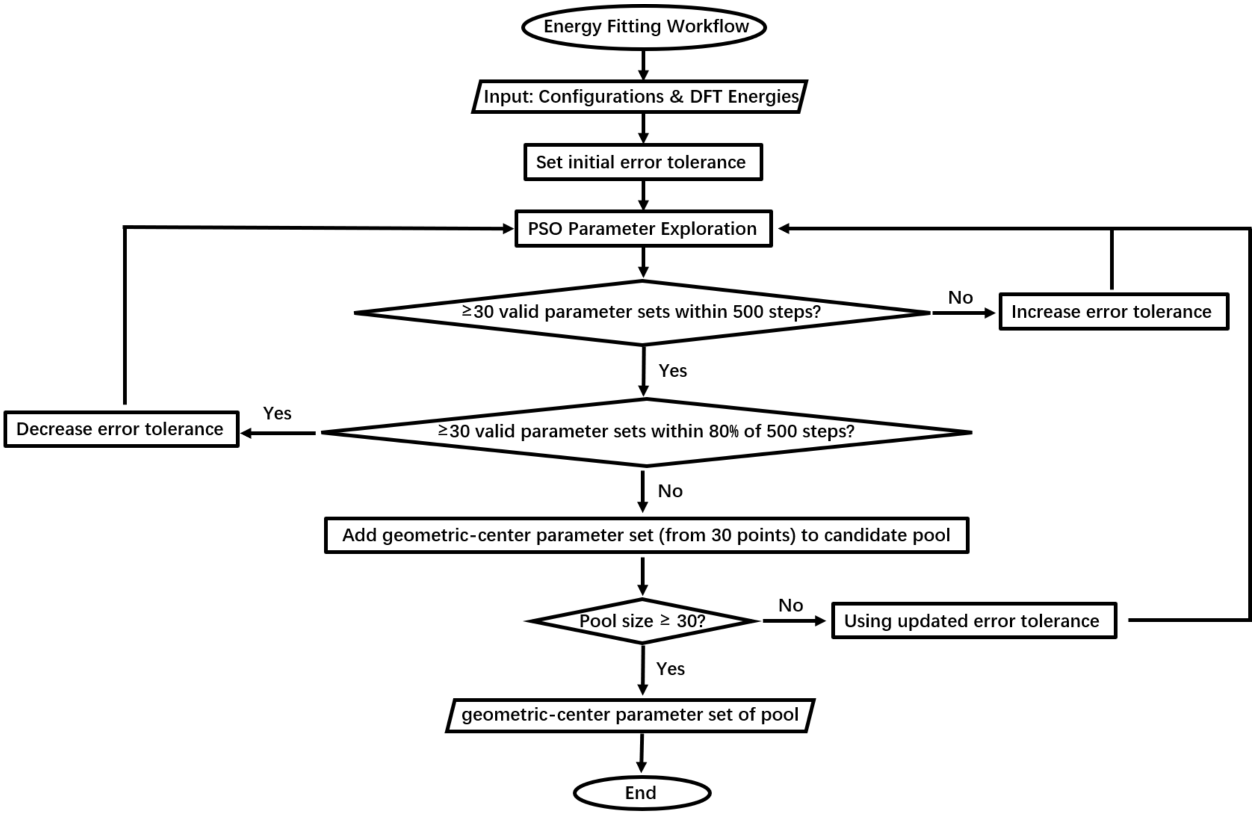

Figure S1: Flowchart of a single PSO parameter fitting run. The full parameterization strategy repeats this process 10 times, followed by geometric-center-based selection of the final representative.

Potential Energy Surfaces

This section summarizes PES scans of guest molecule (C_2H_4, CH_3OH, CO_2, H_2O, NH_3) binding on (1) twelve TMCs which are used to fit 12-6-4 parameters, (2) seven MOF-74 variants (Mg, Mn, Fe, Co, Ni, Cu, Zn), and (3) Cu-BTC for validation. Each figure compares 12-6-4 and DREIDING/UFF with DFT.

—

Al(III)-TMC

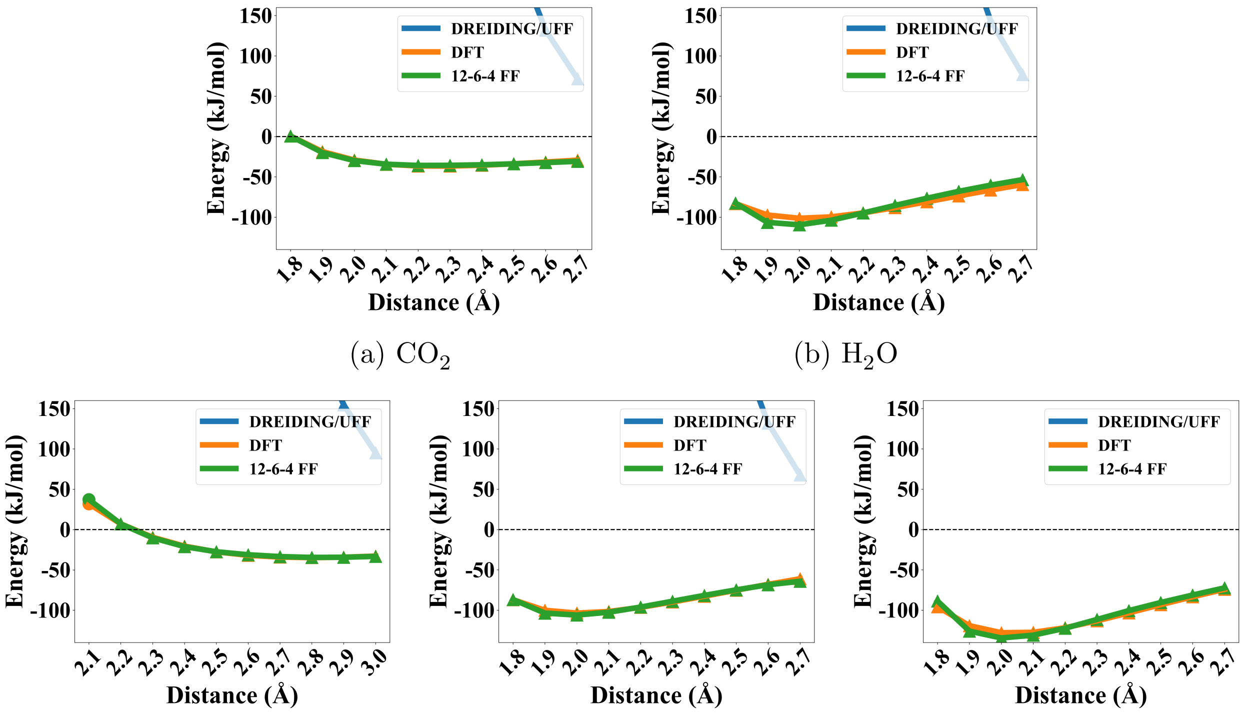

(a) CO_2 (b) H_2O

(c) C_2H_4 (d) CH_3OH (e) NH_3

Figure S2: PES scans for CO_2, H_2O, C_2H_4, CH_3OH and NH_3 binding on Al(III)-TMC.

—

Mn(III)-TMC

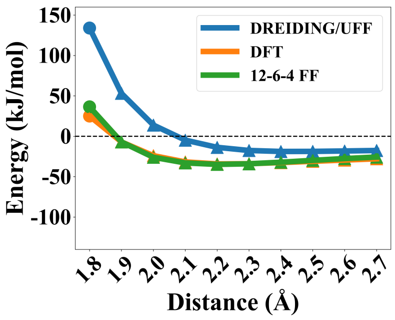

(a) CO_2

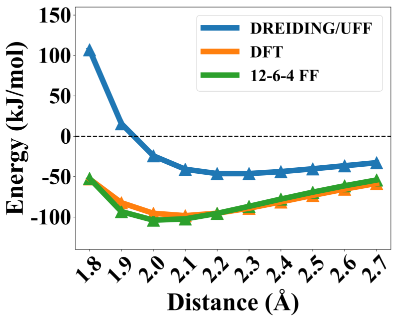

(b) H_2O

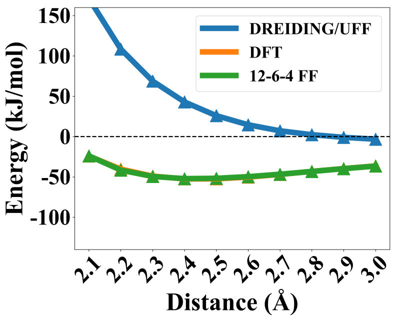

(c) C_2H_4

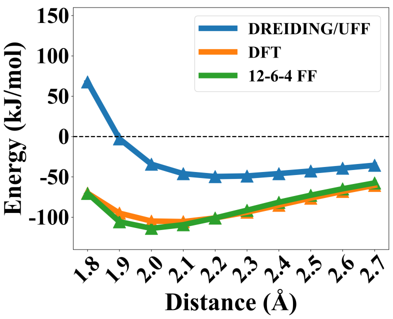

(d) CH_3OH

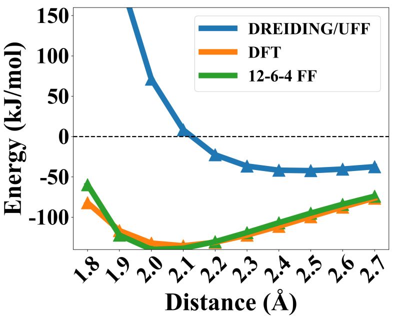

(e) NH_3

Figure S3: PES scans for CO_2, H_2O, C_2H_4, CH_3OH and NH_3 binding on Mn(III)-TMC.

Cr(III)-TMC

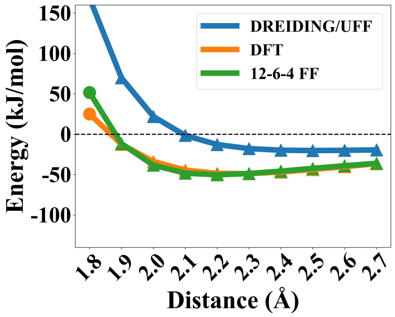

(a) CO_2

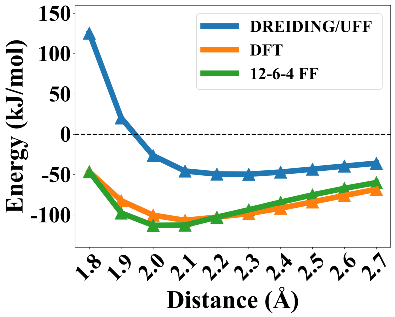

(b) H_2O

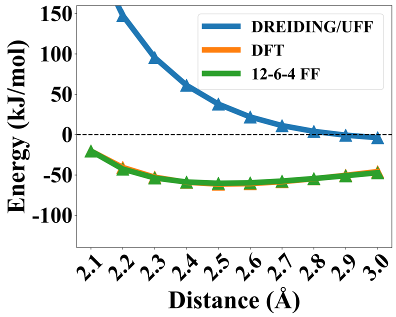

(c) C_2H_4

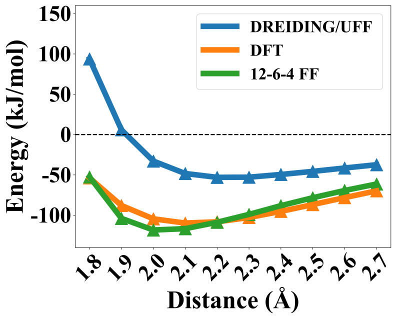

(d) CH_3OH

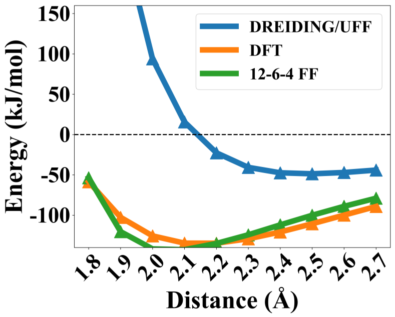

(e) NH_3

Figure S4: PES scans for CO_2, H_2O, C_2H_4, CH_3OH and NH_3 binding on Cr(III)-TMC.

Fe(III)-TMC

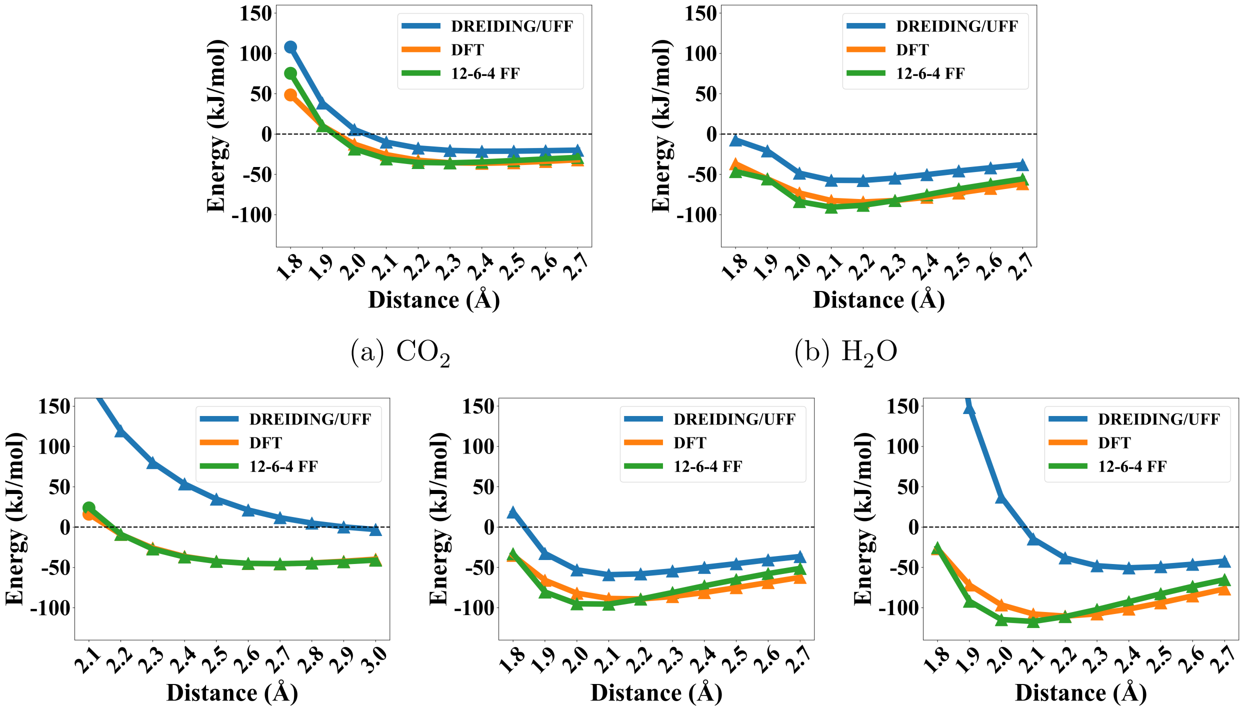

(a) CO_2 (b) H_2O

(c) C_2H_4 (d) CH_3OH (e) NH_3

Figure S5: PES scans for CO_2, H_2O, C_2H_4, CH_3OH and NH_3 binding on Fe(III)-TMC.

Co(III)-TMC

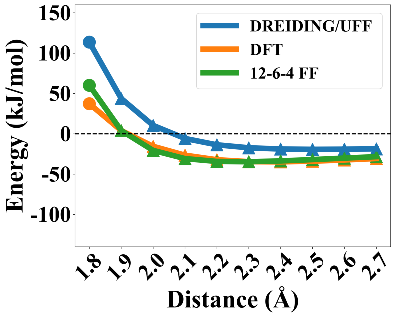

(a) CO_2

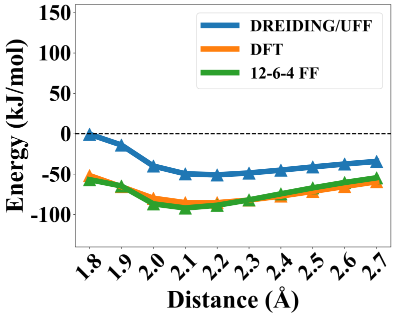

(b) H_2O

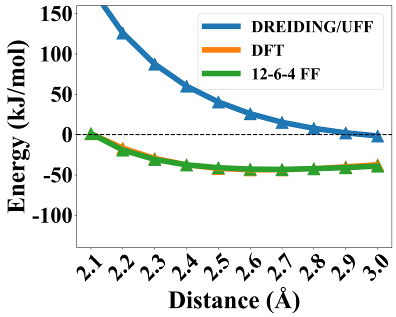

(c) C_2H_4

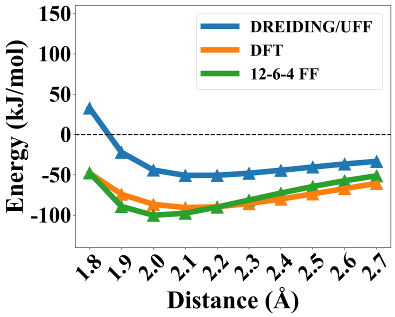

(d) CH_3OH

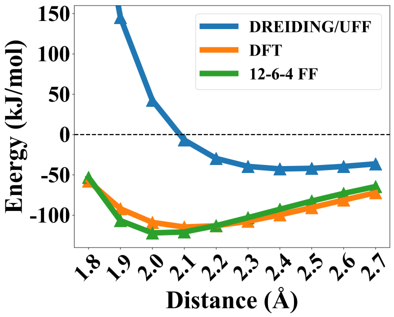

(e) NH_3

Figure S6: PES scans for CO_2, H_2O, C_2H_4, CH_3OH and NH_3 binding on Co(III)-TMC.

Co(II)-TMC

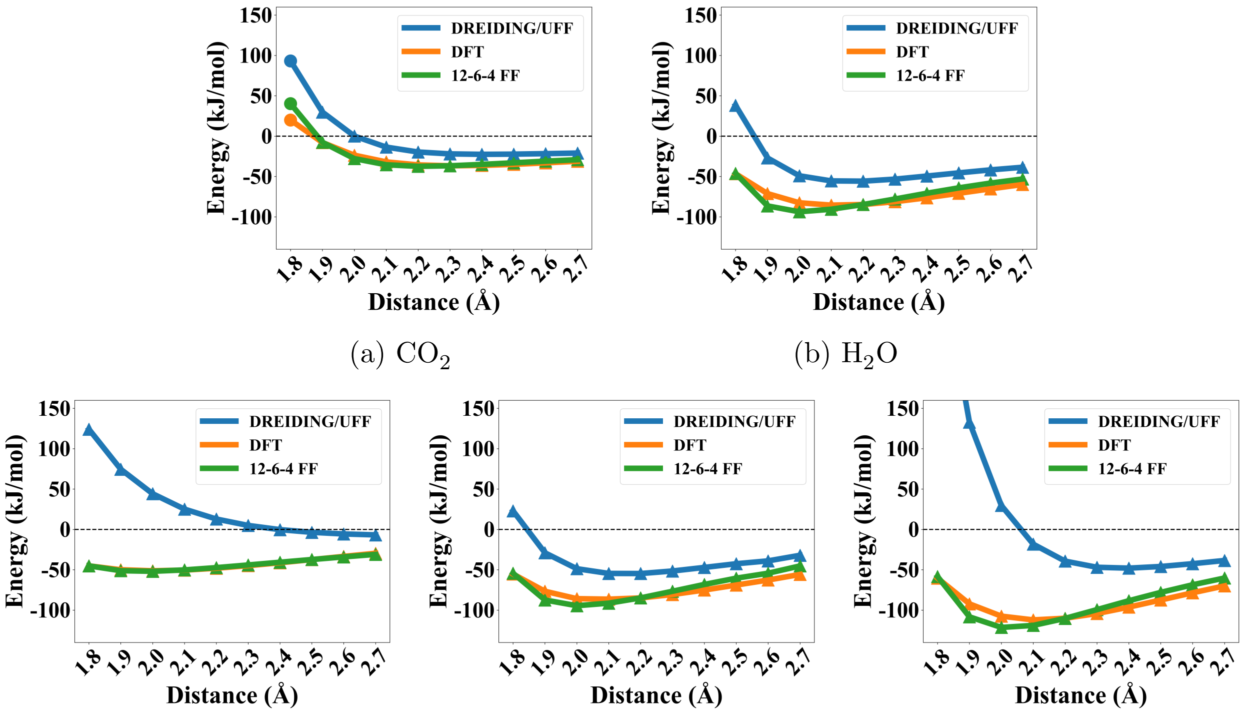

(a) CO_2 (b) H_2O

(c) C_2H_4 (d) CH_3OH (e) NH_3

Figure S7: PES scans for CO_2, H_2O, C_2H_4, CH_3OH and NH_3 binding on Co(II)-TMC.

Cu(II)-TMC

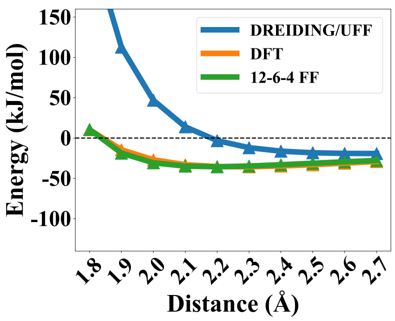

(a) CO_2

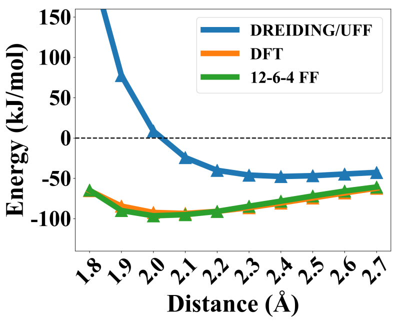

(b) H_2O

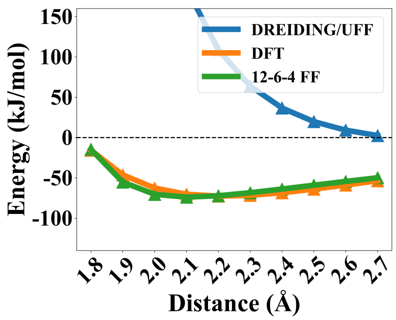

(c) C_2H_4

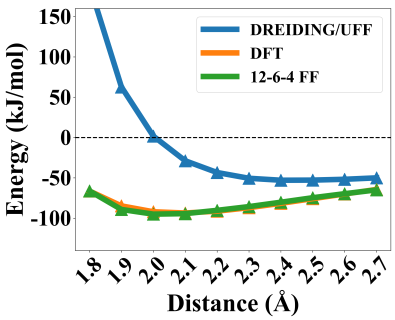

(d) CH_3OH

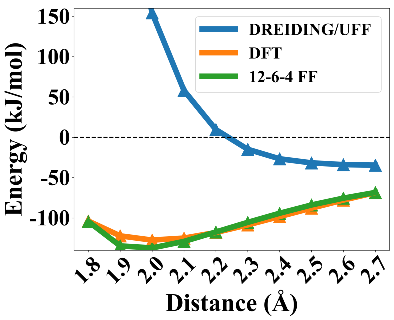

(e) NH_3

Figure S8: PES scans for CO_2, H_2O, C_2H_4, CH_3OH and NH_3 binding on Cu(II)-TMC.

Fe(II)-TMC

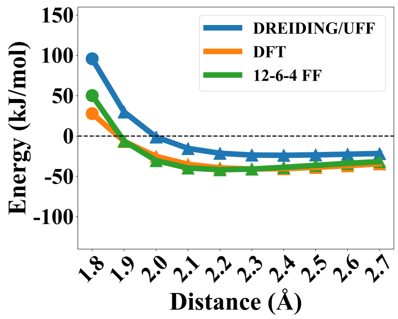

(a) CO_2

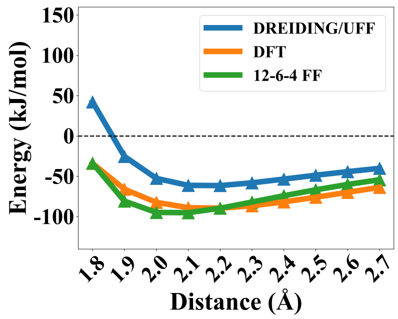

(b) H_2O

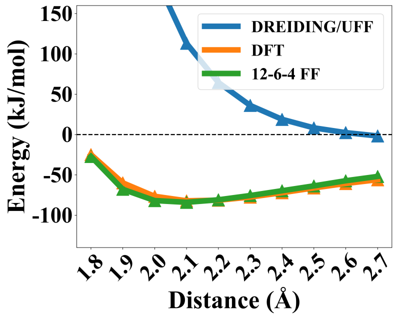

(c) C_2H_4

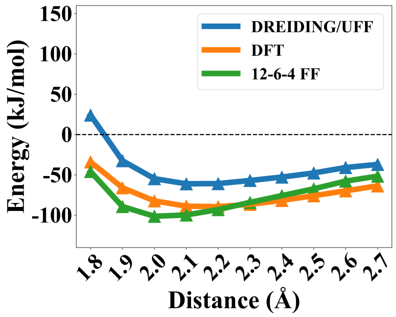

(d) CH_3OH

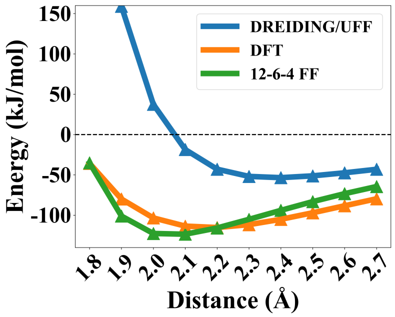

(e) NH_3

Figure S9: PES scans for CO_2, H_2O, C_2H_4, CH_3OH and NH_3 binding on Fe(II)-TMC.

Mg(II)-TMC

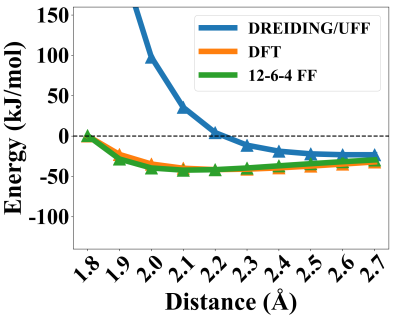

(a) CO_2

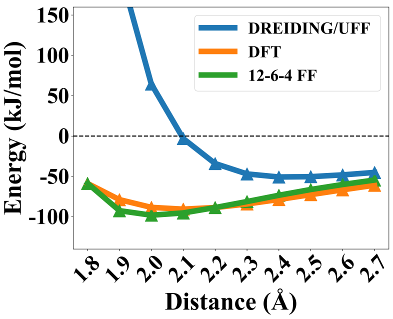

(b) H_2O

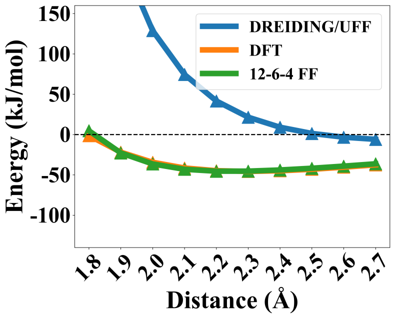

(c) C_2H_4

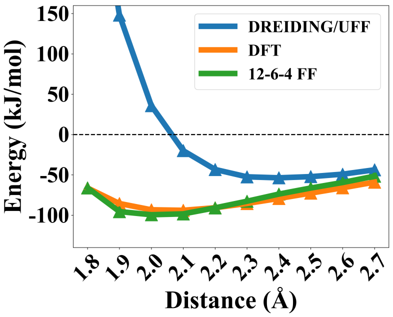

(d) CH_3OH

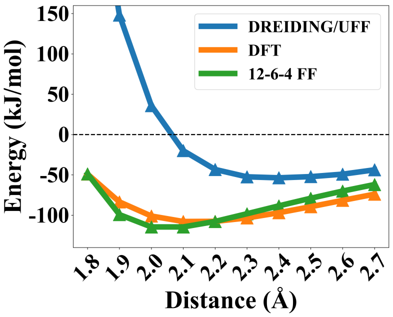

(e) NH_3

Figure S10: PES scans for CO_2, H_2O, C_2H_4, CH_3OH and NH_3 binding on Mg(II)-TMC.

Mn(II)-TMC

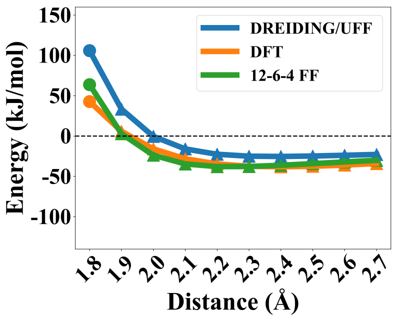

(a) CO_2

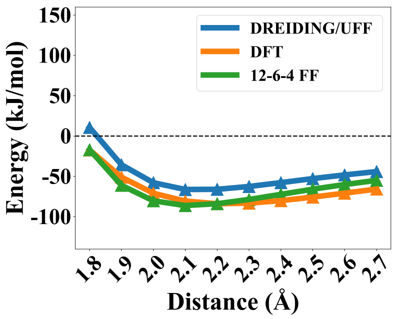

(b) H_2O

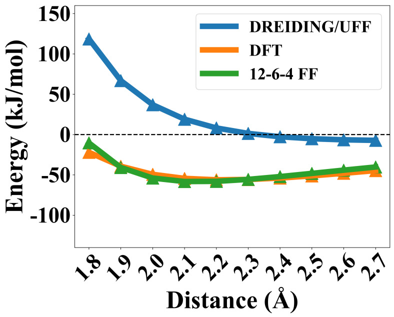

(c) C_2H_4

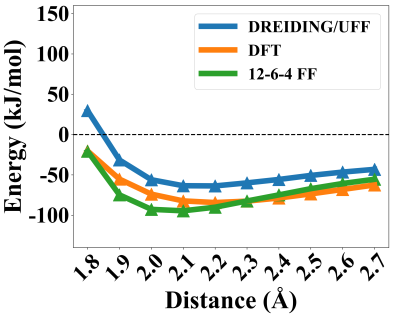

(d) CH_3OH

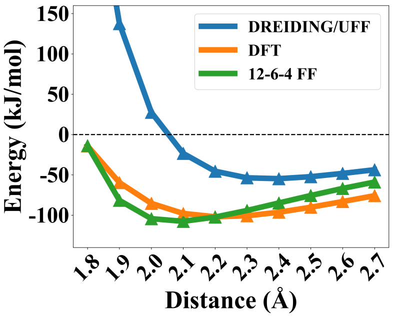

(e) NH_3

Figure S11: PES scans for CO_2, H_2O, C_2H_4, CH_3OH and NH_3 binding on Mn(II)-TMC.

Ni(II)-TMC

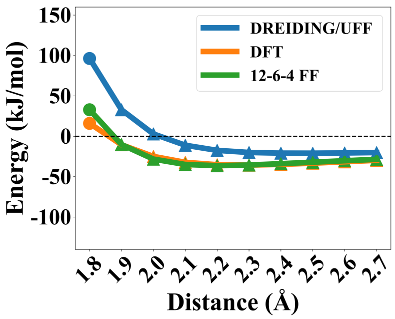

(a) CO_2

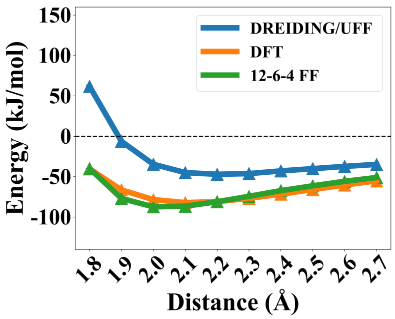

(b) H_2O

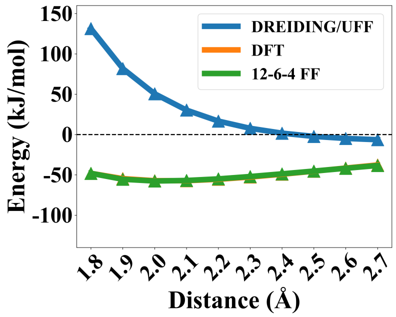

(c) C_2H_4

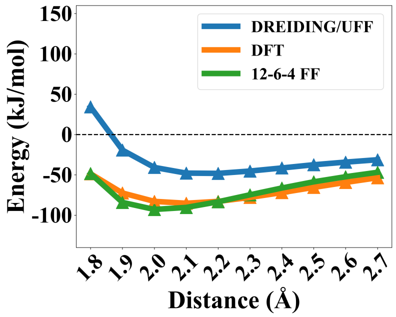

(d) CH_3OH

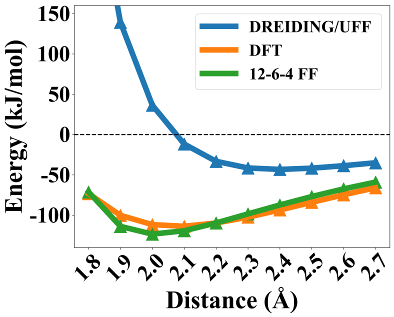

(e) NH_3

Figure S12: PES scans for CO_2, H_2O, C_2H_4, CH_3OH and NH_3 binding on Ni(II)-TMC.

Zn(II)-TMC

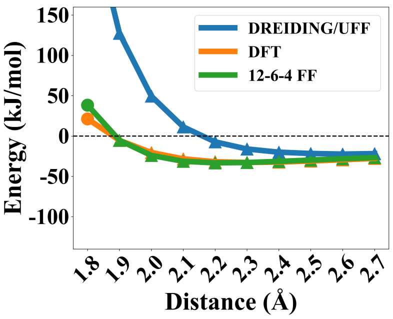

(a) CO_2

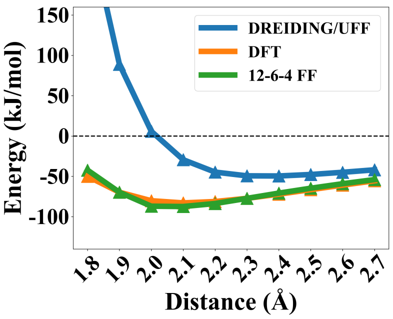

(b) H_2O

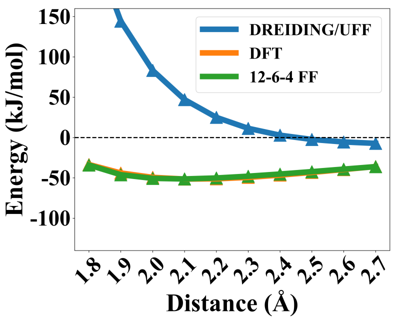

(c) C_2H_4

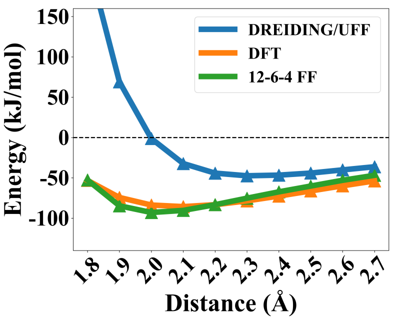

(d) CH_3OH

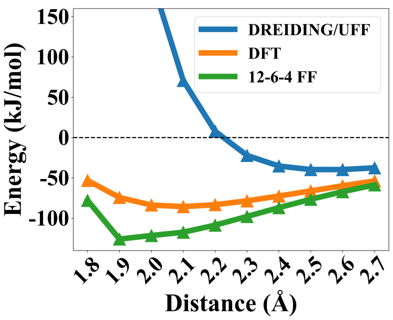

(e) NH_3

Figure S13: PES scans for CO_2, H_2O, C_2H_4, CH_3OH and NH_3 binding on Zn(II)-TMC.

Co-MOF-74

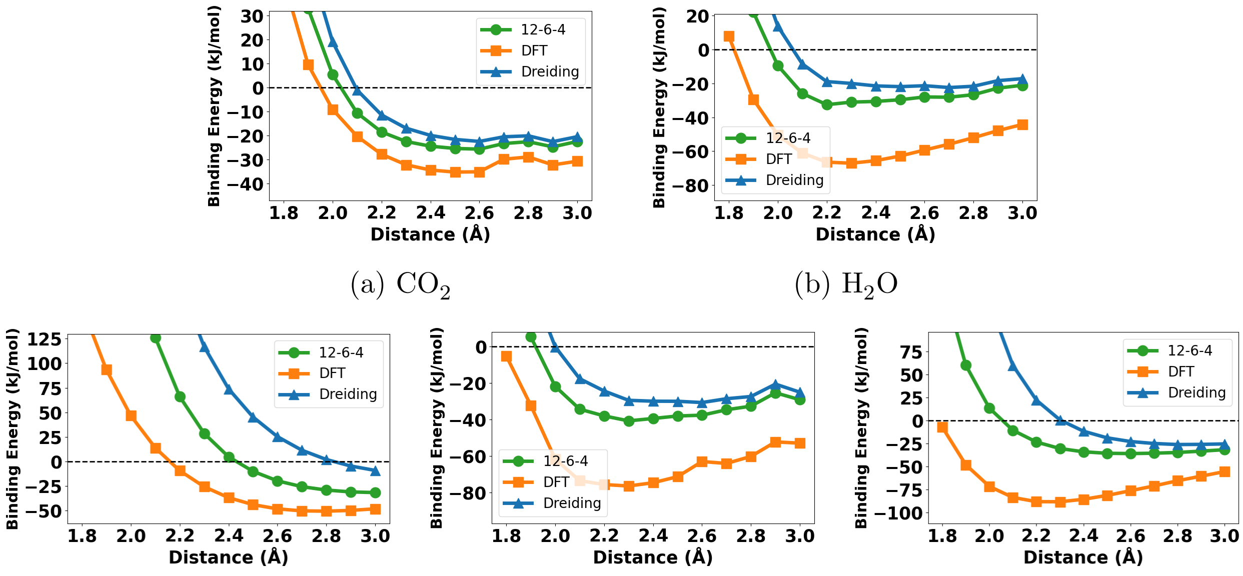

(a) CO_2 (b) H_2O

(c) C_2H_4 (d) CH_3OH (e) NH_3

Figure S14: PES scans for CO_2, H_2O, C_2H_4, CH_3OH and NH_3 binding on Co-MOF-74.

Cu-MOF-74

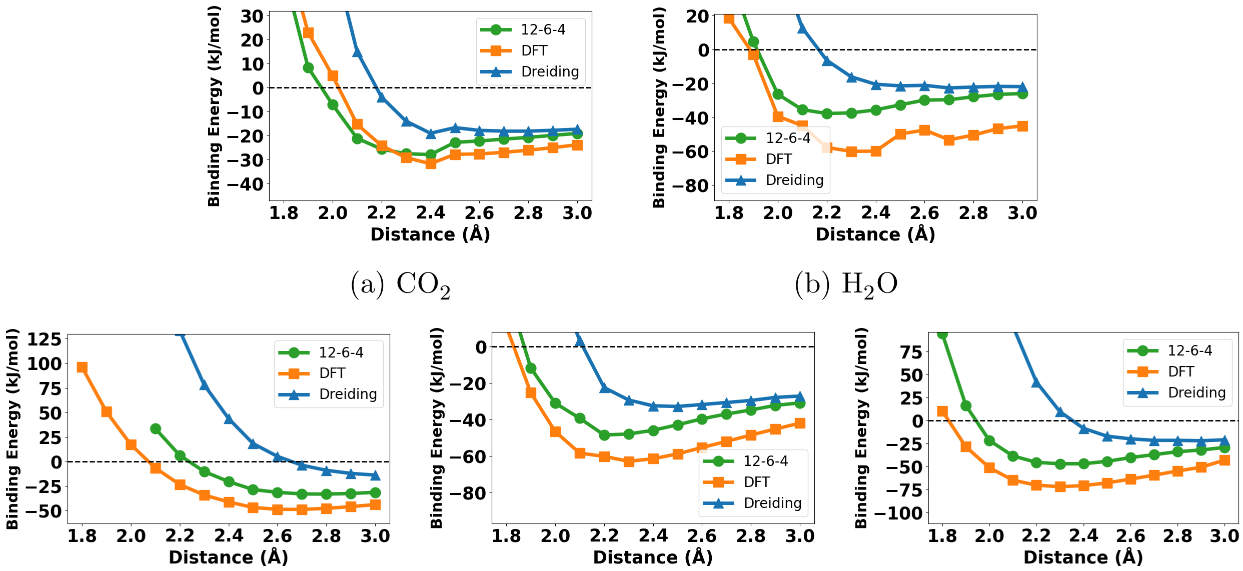

(a) CO_2 (b) H_2O

(c) C_2H_4 (d) CH_3OH (e) NH_3

Figure S15: PES scans for CO_2, H_2O, C_2H_4, CH_3OH and NH_3 binding on Cu-MOF-74.

Fe-MOF-74

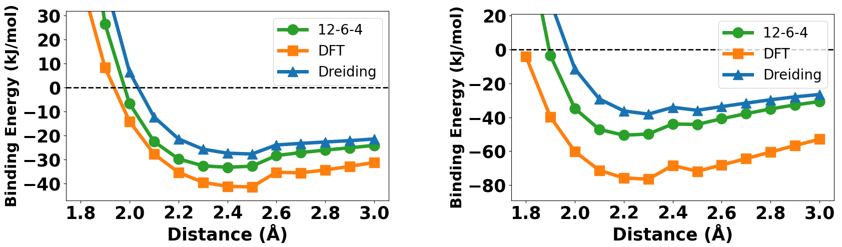

(a) CO_2 (b) H_2O

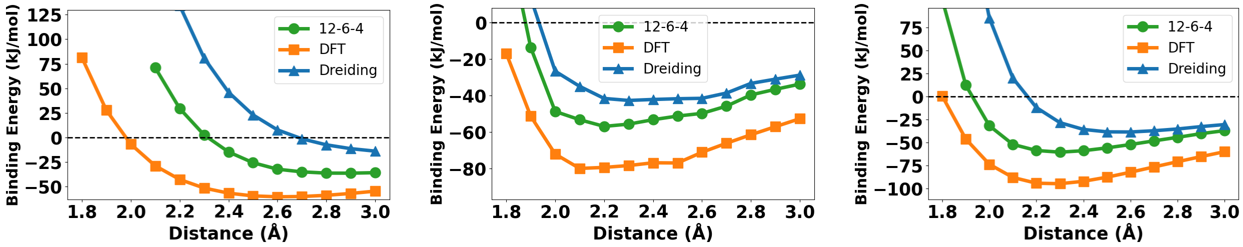

(c) C_2H_4 (d) CH_3OH (e) NH_3

Figure S16: PES scans for CO_2, H_2O, C_2H_4, CH_3OH and NH_3 binding on Fe-MOF-74.

Mg-MOF-74

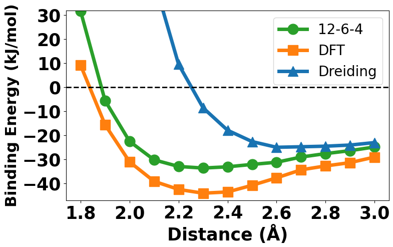

(a) CO_2

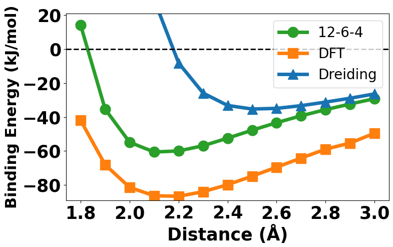

(b) H_2O

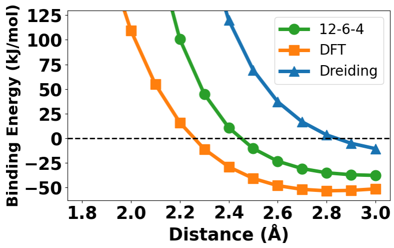

(c) C_2H_4

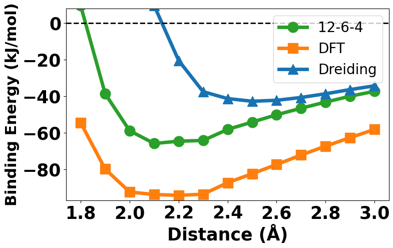

(d) CH_3OH

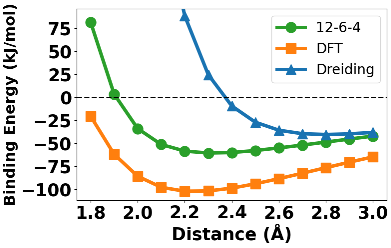

(e) NH_3

Figure S17: PES scans for CO_2, H_2O, C_2H_4, CH_3OH and NH_3 binding on Mg-MOF-74.

Mn-MOF-74

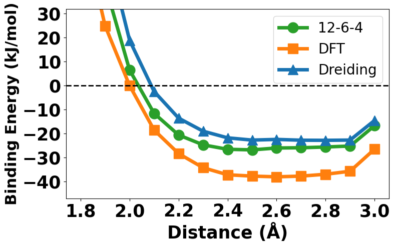

(a) CO_2

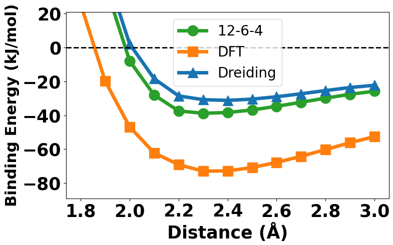

(b) H_2O

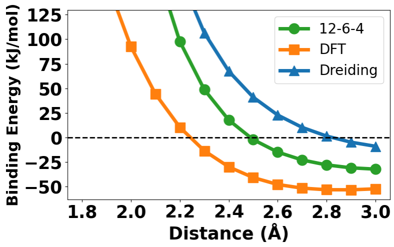

(c) C_2H_4

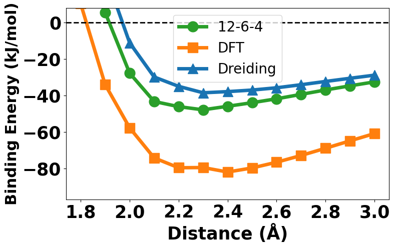

(d) CH_3OH

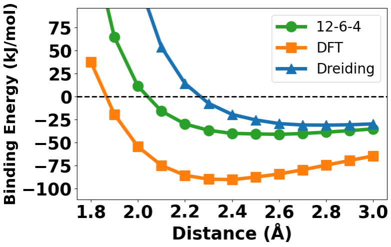

(e) NH_3

Figure S18: PES scans for CO_2, H_2O, C_2H_4, CH_3OH and NH_3 binding on Mn-MOF-74.

Ni-MOF-74

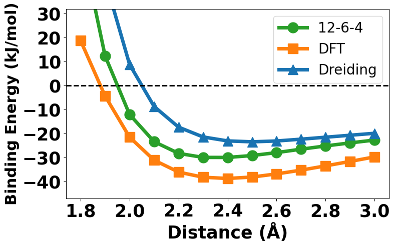

(a) CO_2

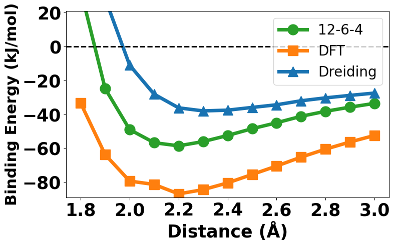

(b) H_2O

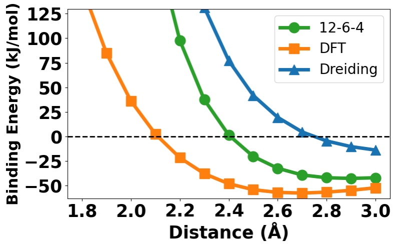

(c) C_2H_4

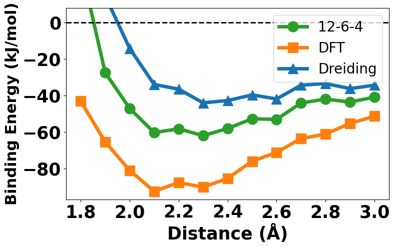

(d) CH_3OH

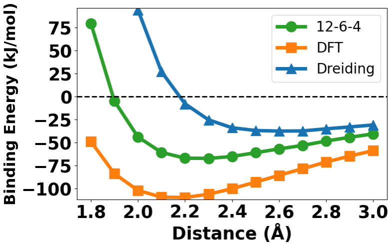

(e) NH_3

Figure S19: PES scans for CO_2, H_2O, C_2H_4, CH_3OH and NH_3 binding on Ni-MOF-74.

Zn-MOF-74

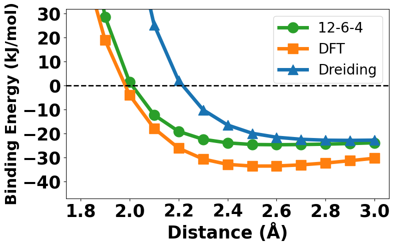

(a) CO_2

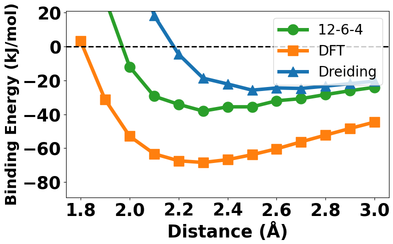

(b) H_2O

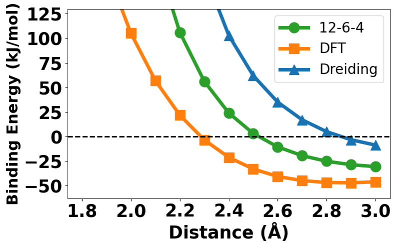

(c) C_2H_4

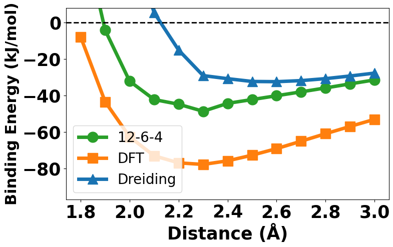

(d) CH_3OH

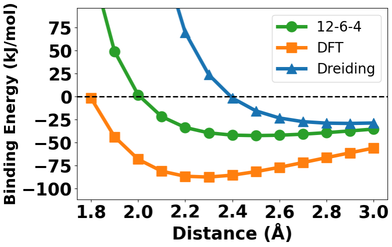

(e) NH_3

Figure S20: PES scans for CO_2, H_2O, C_2H_4, CH_3OH and NH_3 binding on Zn-MOF-74.

Cu-BTC

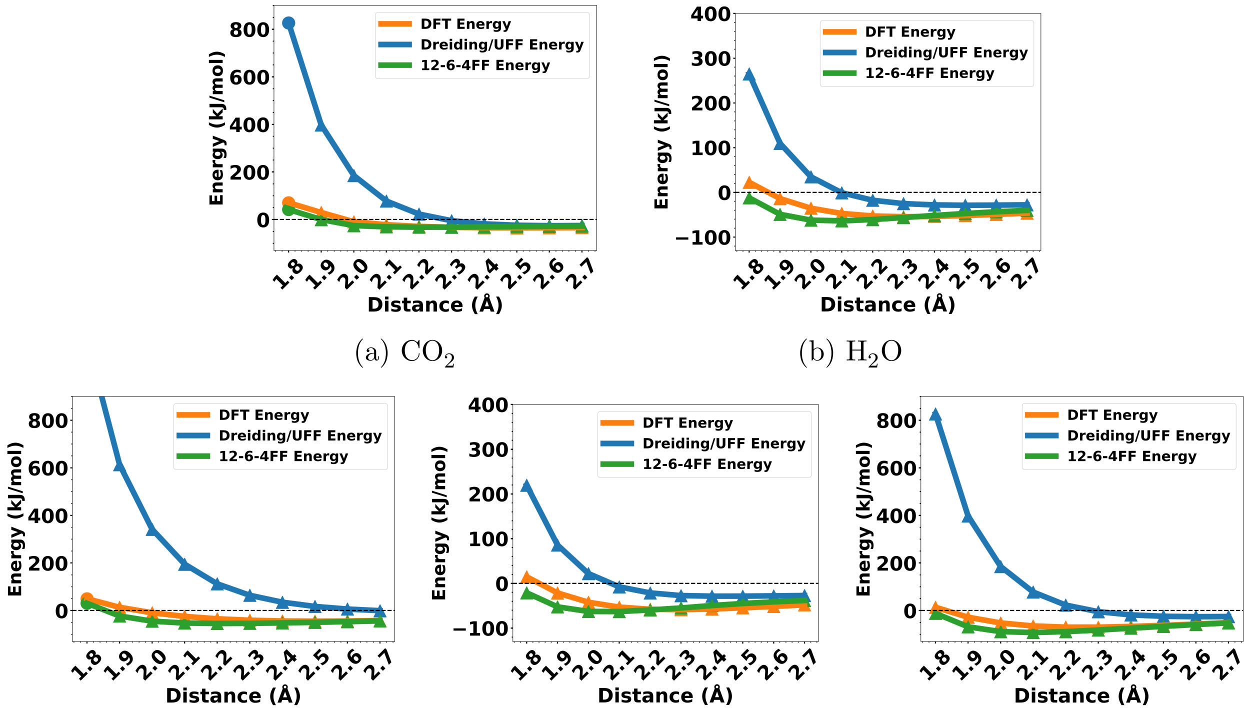

(a) CO_2 (b) H_2O

(c) C_2H_4 (d) CH_3OH (e) NH_3

Figure S21: PES scans for CO_2, H_2O, C_2H_4, CH_3OH and NH_3 binding on Cu-BTC.

Comparison of Parametrization Schemes

To assess the impact of different fitting assumptions, parameter constraints, and model sensitivities on the accuracy and transferability of the 12-6-4 force field, multiple parametrization schemes were systematically tested. This section presents: (1) effect of parameter space dimensionality, (2) effect of search range of κ ($\kappa = C_4/C_6$), and (3) effect of water model.

Parameter Space

All the 12-6-4 parameters used in generating PESs shown in the main text and the previous section were obtained by fitting C_{12}, C_6, and C_4 simultaneously (referred to as S6). In addition to that, we also considered five alternative fitting schemes:

- **S1**: C_{12} and C_6 from DREIDING/UFF/TraPPE were retained. A single base C_4 was fitted, then scaled for each metal–guest pair as:

$$C_4^{\text{pair}} = C_4^{\text{base}} \times q_{\text{metal}}^2 \times \varepsilon_{\text{guest}}.$$

- **S2**: Same as S1, but also scaled by σ_{guest}^6:

$$C_4^{\text{pair}} = C_4^{\text{base}} \times q_{\text{metal}}^2 \times \varepsilon_{\text{guest}} \times \sigma_{guest}^6.$$

- **S3**: C_{12} and C_6 from DREIDING/UFF/TraPPE retained. A separate C_4 was fitted for each guest molecule, and then scaled by q_{metal}^2 for each metal-guest pair.
- **S4**: C_{12} and C_6 from DREIDING/UFF/TraPPE retained. A separate C_4 was fitted for each metal ion, then scaled by $\varepsilon_{\text{guest}} \times \sigma_{guest}^6$ for each pair.
- **S5**: C_{12} and C_6 from DREIDING/UFF/TraPPE retained. A unique C_4 was directly fitted for each metal-guest pair.

- **S6 (default)**: For each metal–guest pair, all three parameters (C_{12}, C_6, and C_4) were simultaneously refitted. This is the strategy used in our GCMC simulations.

The rationale behind S1-S5 is (1) C_4 is proportional to the polarizability of the guest α_{guest}, which is related to ε_{guest}; (2) to reduce the dimensionality of the parameter space. As shown in Figure S22 however, S6 consistently achieves the lowest or one of the lowest MAEs across all guests. This suggests that a full refitting of C_{12}, C_6, and C_4 is necessary.

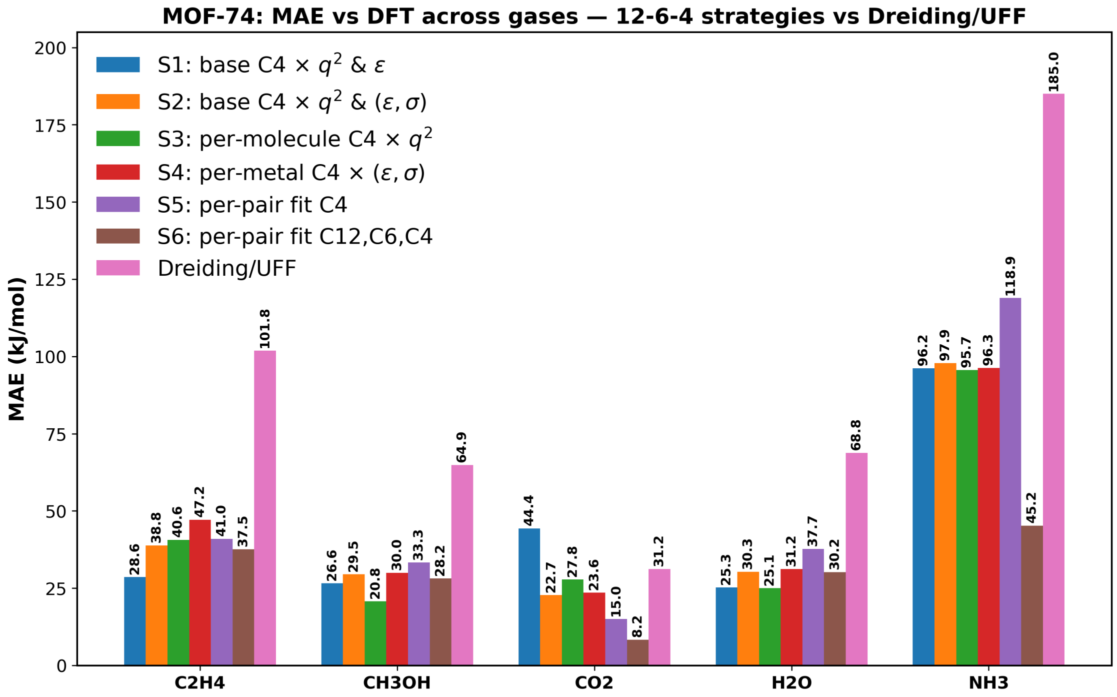

Figure S22: Mean absolute errors (MAEs) between classical and DFT binding energies for MOF-74 across five gases, comparing different 12–6–4 parameterization strategies (S1–S6) against DREIDING/UFF.

Search Range of κ

As discussed above, we set constraint on the κ parameter to avoid unphysical C_4 values from the fitting. To further evaluate the effect of κ search range, two independent sets of fittings were performed with κ constrained to 0–3 Å^{-2} and 0–6 Å^{-2}, respectively. As shown in Figure S23, the results of the two fitting processes were almost identical, in terms of

mean absolute error of the PESs compared to DFT (31.7 kJ/mol vs 31.2 kJ/mol). This demonstrated that the parametrization is robust with respect to the search range of κ.

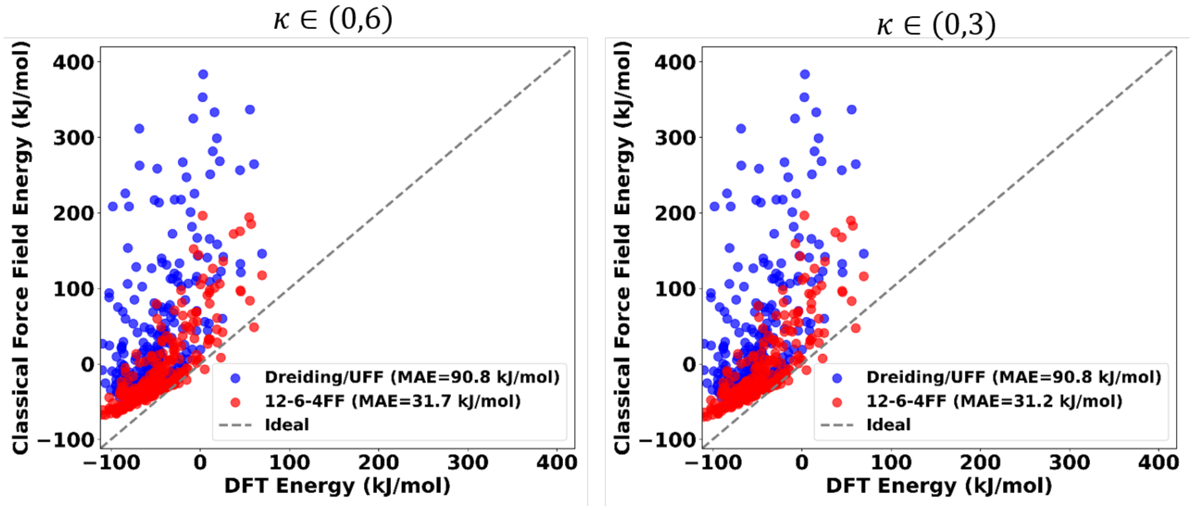

Figure S23: Comparison of fitted 12–6–4 force fields obtained using different κ search ranges. right: κ constrained to 0–3 Å^{-2}; left: κ constrained to 0–6 Å^{-2}. Each panel shows correlations between classical force field and DFT interaction energies for all MOF-74 configurations used in fitting.

Choice of Water Model

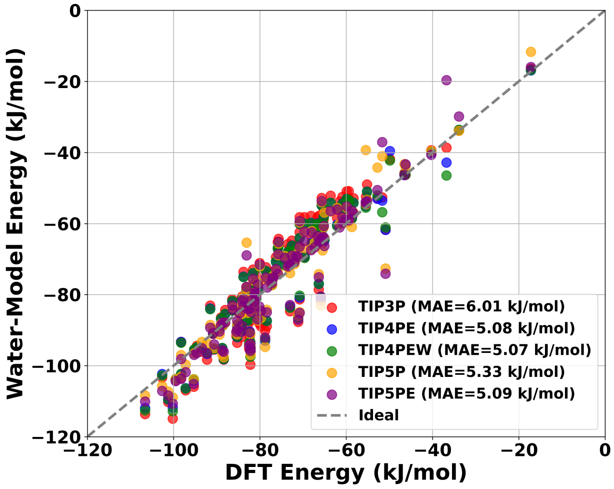

Figure S24: Correlation plots between 12-6-4 and DFT TMC-H_2O binding energies for five rigid water models: TIP3P, TIP4PE, TIP4PEW, TIP5P, and TIP5PE. Dashed gray line: parity line.

To evaluate the sensitivity of the fitted 12-6-4 parameters to the choice of water model, we scanned the TMC-H_2O binding PESs with five widely used rigid water models (TIP3P, TIP4PE, TIP4PEW, TIP5P, and TIP5PE). Figure S24 shows the correlation between 12-6-4 and DFT energies, from which we can tell that all five water models yielded very similar results, with mean absolute errors ranging from 5.07 to 6.01 kJ mol^{-1}. This suggests that our parametrization procedure is largely insensitive to the water model chosen, providing further evidence of the transferability and robustness of our 12-6-4 force field.

Adsorption Isotherms

CO_2 adsorption in M-MOF-74

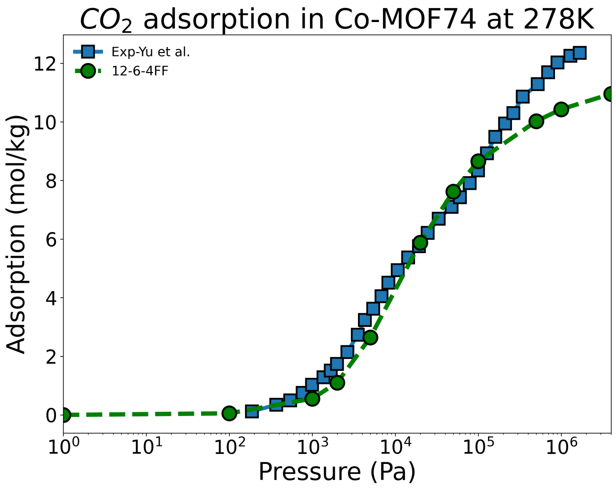

Figure S25: CO_2 adsorption in Co-MOF-74 at 278 K. Experimental data from Yu *et al.*[24] (squares); results from the fitted 12–6–4 force field are plotted as green dashed lines.

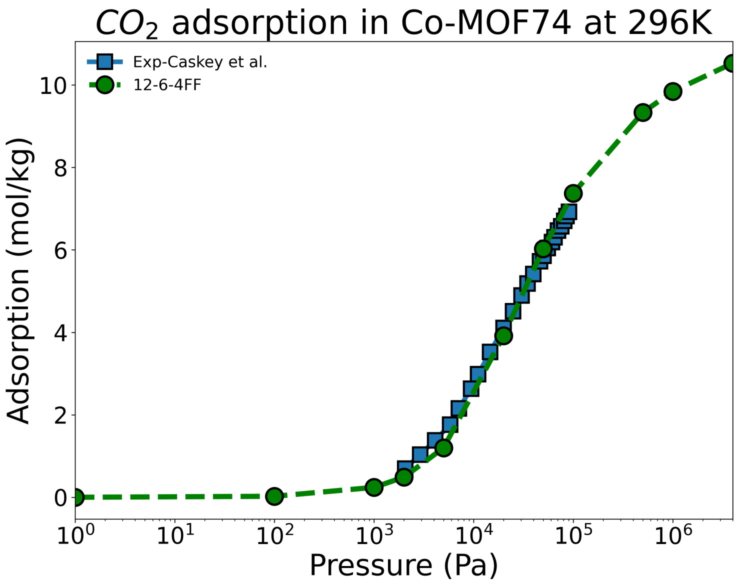

Figure S26: CO_2 adsorption in Co-MOF-74 at 296 K. Experimental data from Caskey *et al.*[25] (squares); results from the fitted 12–6–4 force field are plotted as green dashed lines.

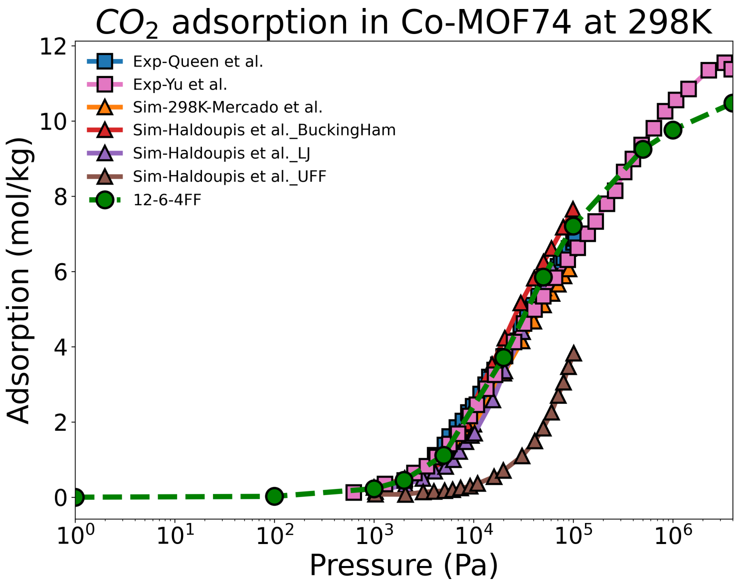

Figure S27: CO_2 adsorption in Co-MOF-74 at 298 K. Experimental data from Queen *et al.*[26] and Yu *et al.*[24] (squares); simulation data from Mercado *et al.*[27] and Haldoupis *et al.*[28] (triangles); results from the fitted 12–6–4 force field are plotted as green dashed lines.

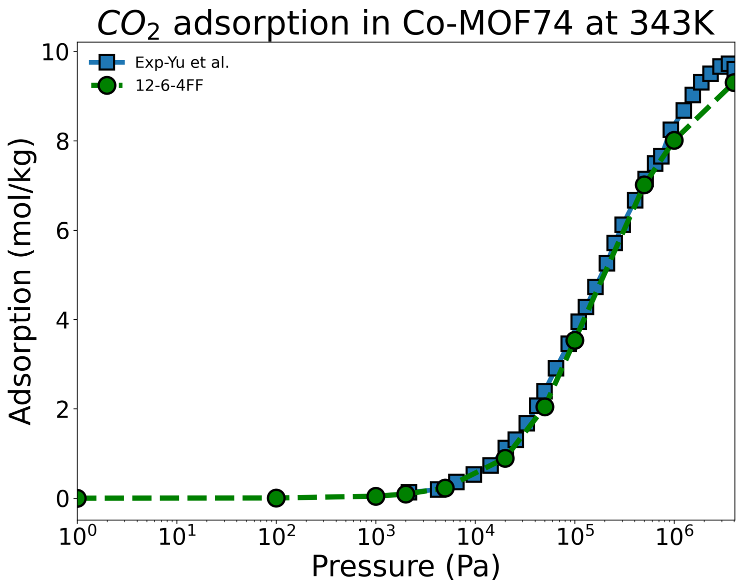

Figure S28: CO_2 adsorption in Co-MOF-74 at 343 K. Experimental data from Yu *et al.*[24] (squares); results from the fitted 12–6–4 force field are plotted as green dashed lines.

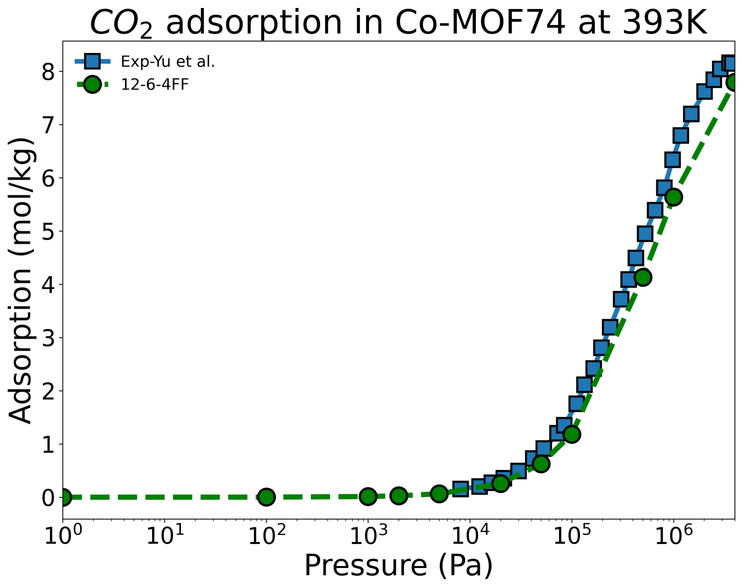

Figure S29: CO_2 adsorption in Co-MOF-74 at 393 K. Experimental data from Yu *et al.*[24] (squares); results from the fitted 12–6–4 force field are plotted as green dashed lines.

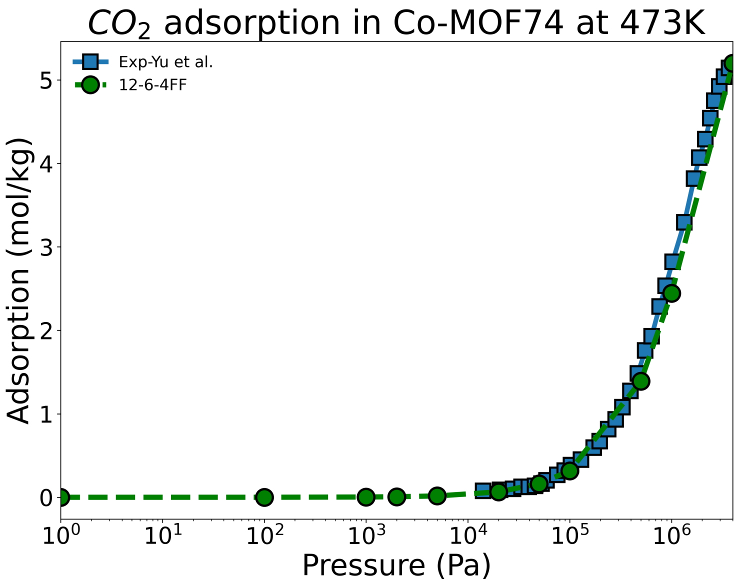

Figure S30: CO_2 adsorption in Co-MOF-74 at 473 K. Experimental data from Yu *et al.*[24] (squares); results from the fitted 12–6–4 force field are plotted as green dashed lines.

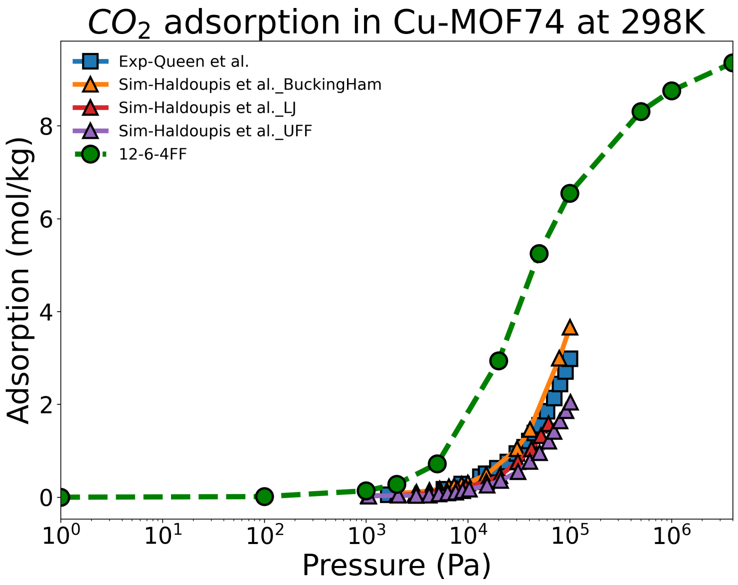

Figure S31: CO_2 adsorption in Cu-MOF-74 at 298 K. Experimental data from Queen *et al.*[26] (squares); simulation data from Haldoupis *et al.*[28] (triangles); results from the fitted 12–6–4 force field are plotted as green dashed lines.

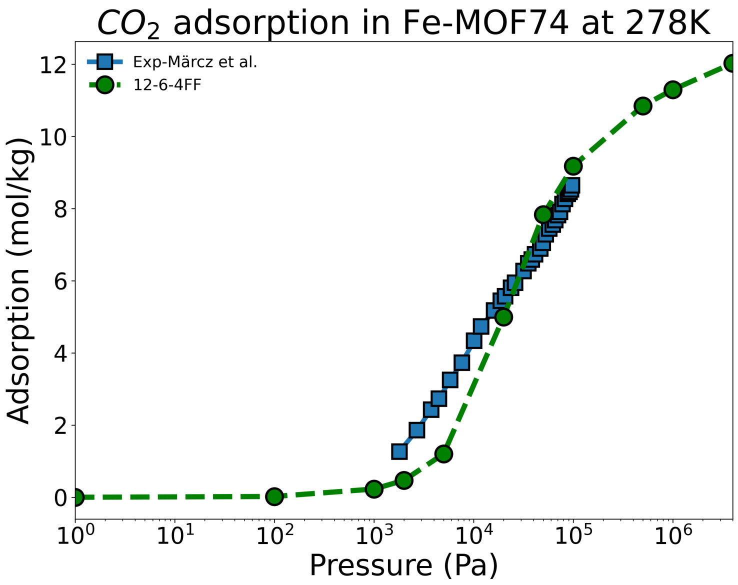

Figure S32: CO_2 adsorption in Fe-MOF-74 at 278 K. Experimental data from Märcz *et al.*[29] (squares); results from the fitted 12–6–4 force field are plotted as green dashed lines.

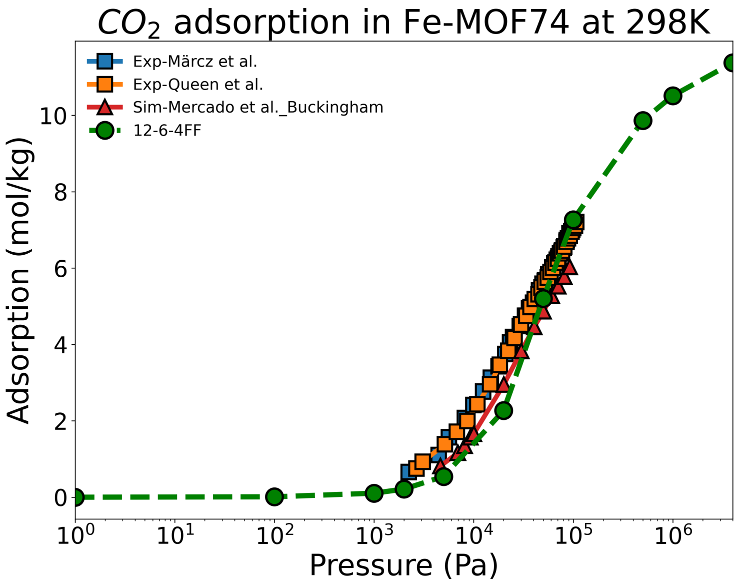

Figure S33: CO_2 adsorption in Fe-MOF-74 at 298 K. Experimental data from Märcz *et al.*[29] and Queen *et al.*[26] (squares); simulation data from Mercado *et al.*[27] (triangles); results from the fitted 12–6–4 force field are plotted as green dashed lines.

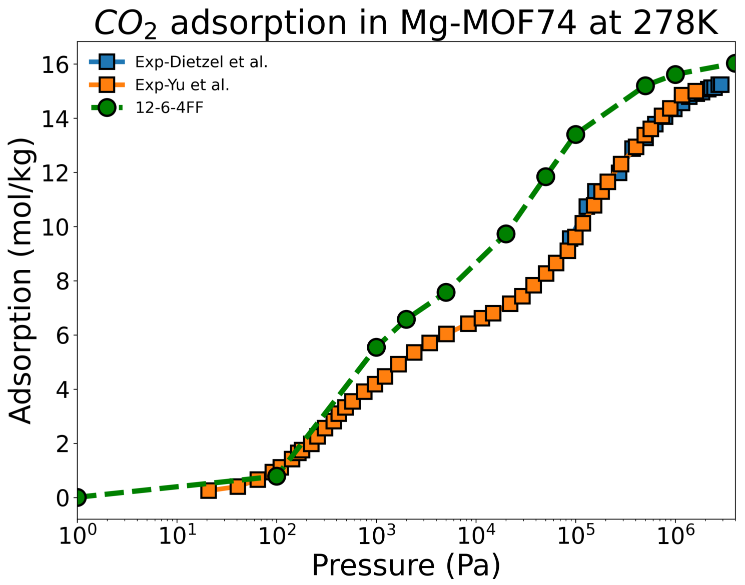

Figure S34: CO_2 adsorption in Mg-MOF-74 at 278 K. Experimental data from Dietzel *et al.*[30] and Yu *et al.*[24] (squares); results from the fitted 12–6–4 force field are plotted as green dashed lines.

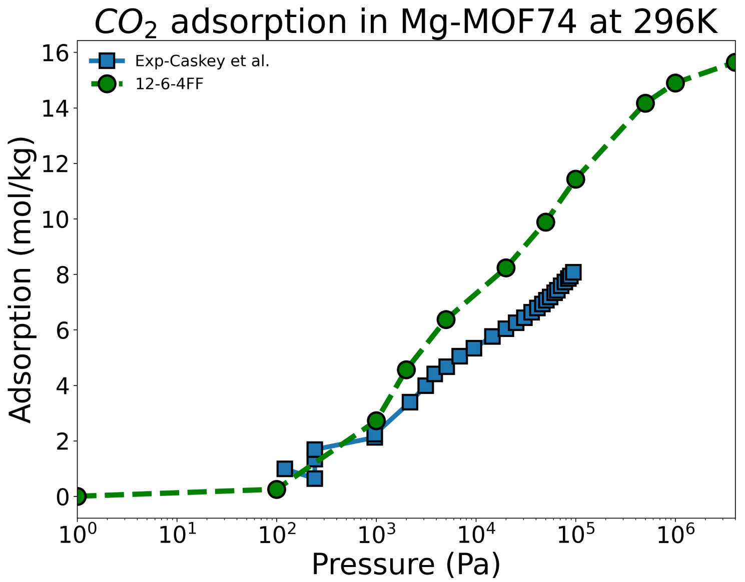

Figure S35: CO_2 adsorption in Mg-MOF-74 at 296 K. Experimental data from Caskey *et al.*[25] (squares); results from the fitted 12–6–4 force field are plotted as green dashed lines.

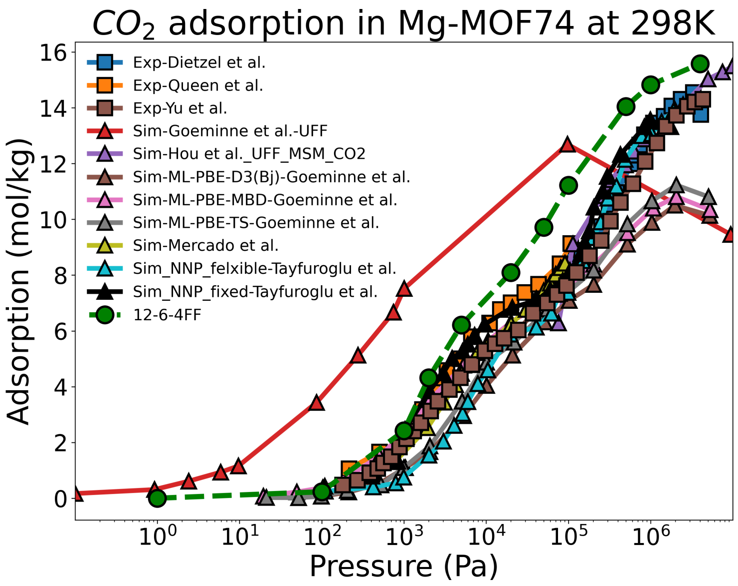

Figure S36: CO_2 adsorption in Mg-MOF-74 at 298 K. Experimental data from Dietzel *et al.*,[30] Queen *et al.*,[26] and Yu *et al.*[24] (squares); simulation data from Tayfuroglu *et al.*,[31] Goeminne *et al.*,[32] Hou *et al.*,[33] and Mercado *et al.*[27] (triangles); results from the fitted 12–6–4 force field are plotted as green dashed lines.

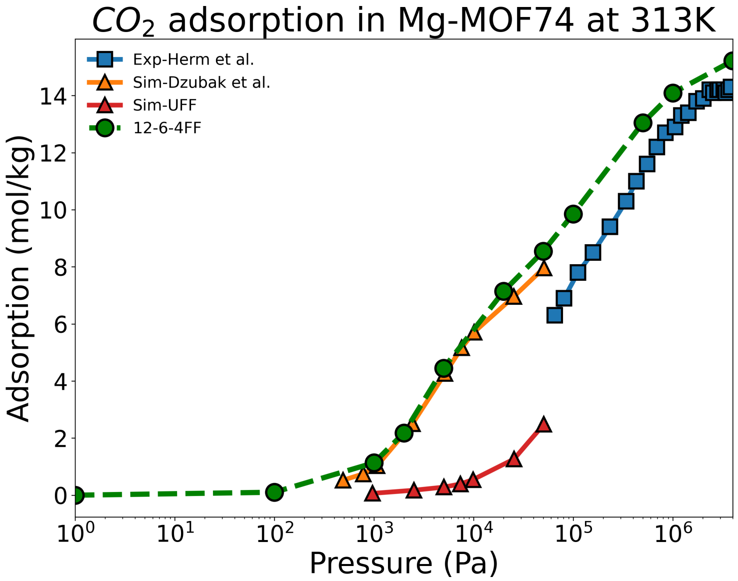

Figure S37: CO_2 adsorption in Mg-MOF-74 at 313 K. Experimental data from Herm *et al.*[34] (squares); simulation data from Dzubak *et al.*[35] (triangles); results from the fitted 12–6–4 force field are plotted as green dashed lines.

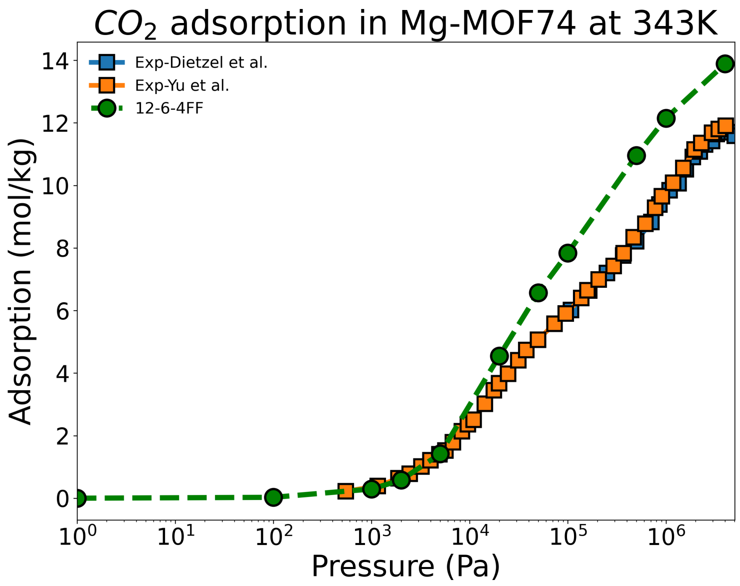

Figure S38: CO_2 adsorption in Mg-MOF-74 at 343 K. Experimental data from Dietzel *et al.*[30] and Yu *et al.*[24] (squares); results from the fitted 12–6–4 force field are plotted as green dashed lines.

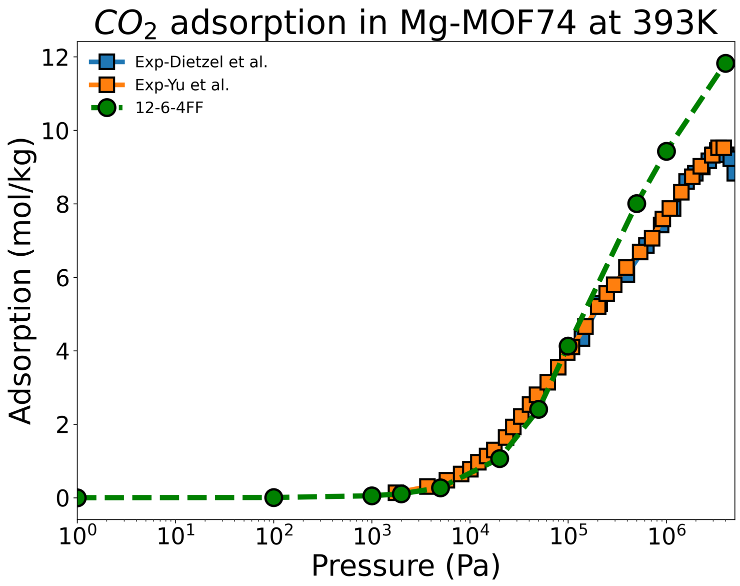

Figure S39: CO_2 adsorption in Mg-MOF-74 at 393 K. Experimental data from Dietzel *et al.*[30] and Yu *et al.*[24] (squares); results from the fitted 12–6–4 force field are plotted as green dashed lines.

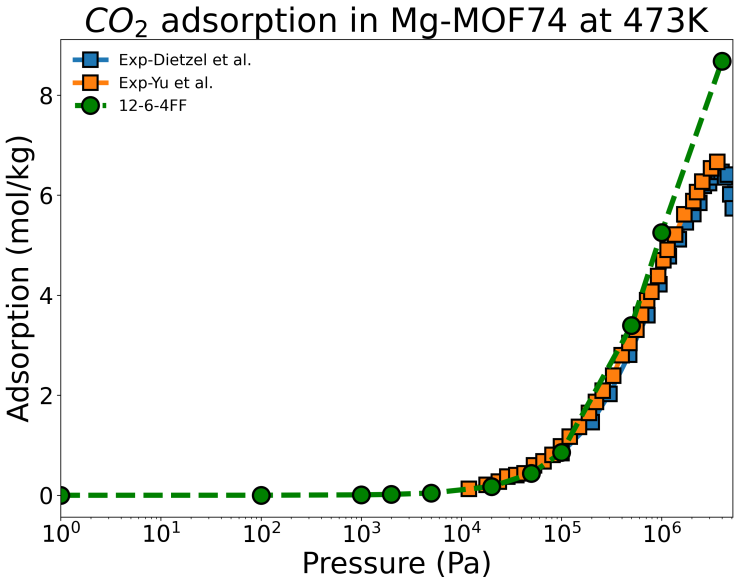

Figure S40: CO_2 adsorption in Mg-MOF-74 at 473 K. Experimental data from Dietzel *et al.*[30] and Yu *et al.*[24] (squares); results from the fitted 12–6–4 force field are plotted as green dashed lines.

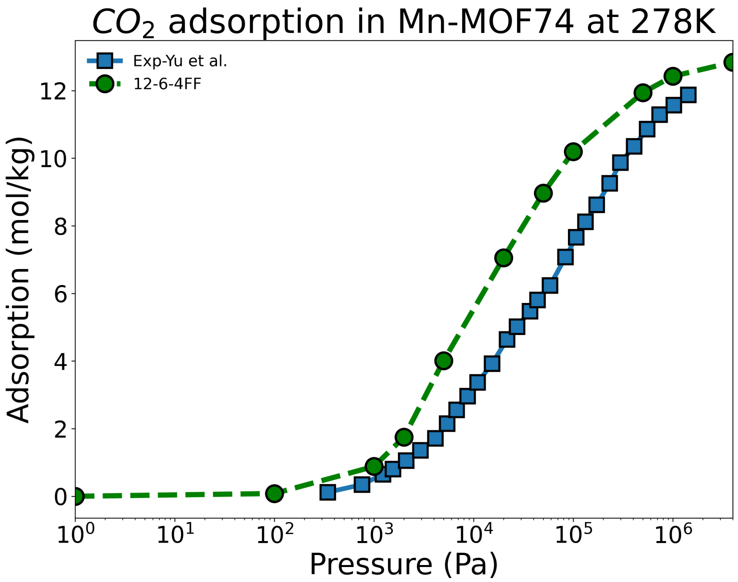

Figure S41: CO_2 adsorption in Mn-MOF-74 at 278 K. Experimental data from Yu *et al.*[24] (squares); results from the fitted 12–6–4 force field are plotted as green dashed lines.

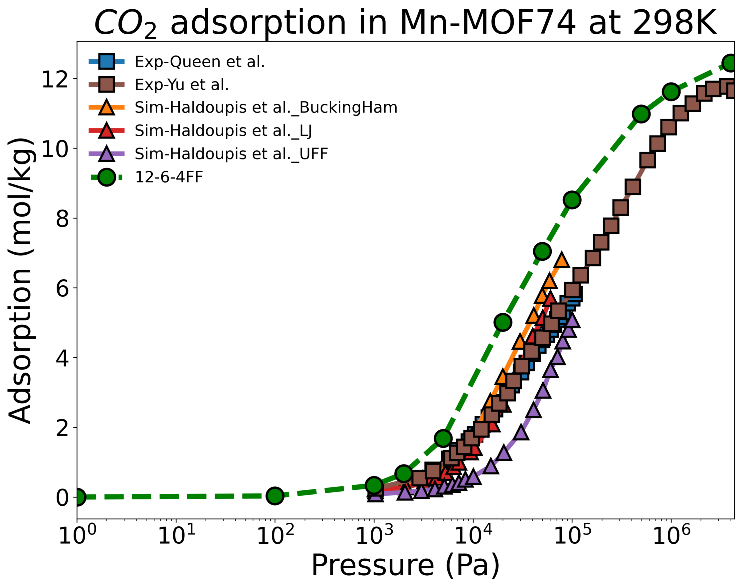

Figure S42: CO_2 adsorption in Mn-MOF-74 at 298 K. Experimental data from Queen *et al.*,[26] Yu *et al.*[24] (squares); simulation results from Haldoupis *et al.*[28] (dashed lines); fitted 12–6–4 force field shown as green dashed lines.

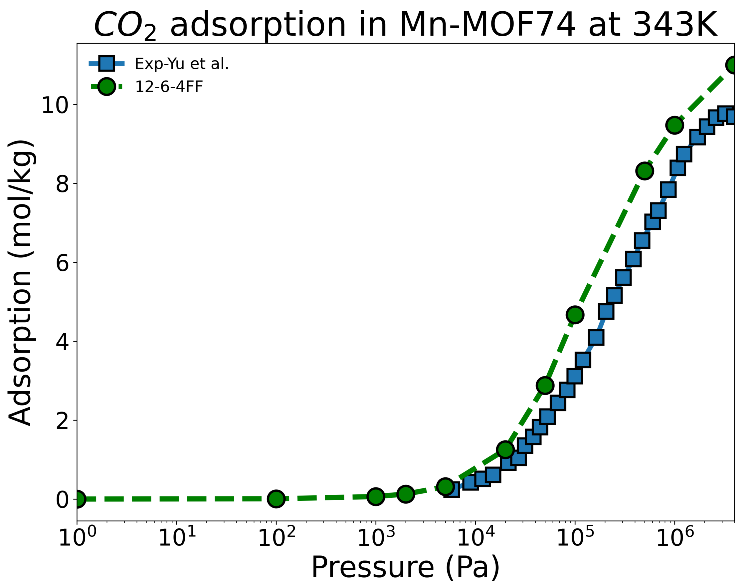

Figure S43: CO_2 adsorption in Mn-MOF-74 at 343 K. Experimental data from Yu *et al.*[24] (squares); fitted 12–6–4 force field shown as green dashed lines.

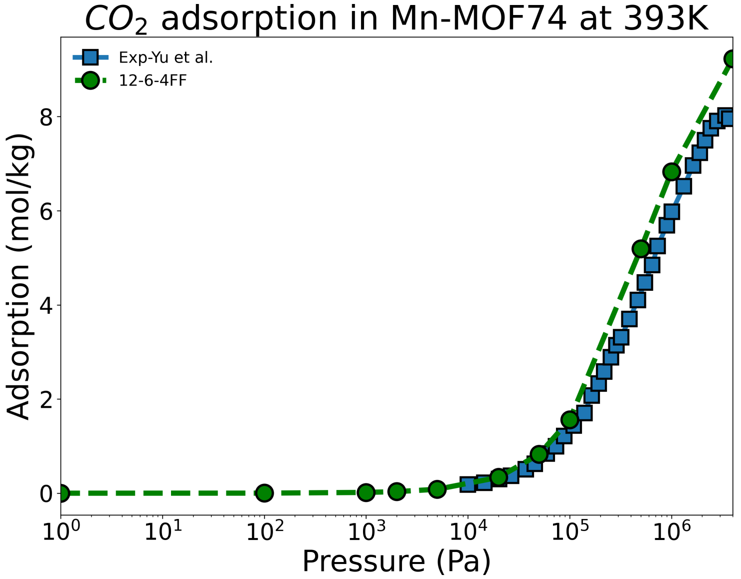

Figure S44: CO_2 adsorption in Mn-MOF-74 at 393 K. Experimental data from Yu *et al.*[24] (squares); fitted 12–6–4 force field shown as green dashed lines.

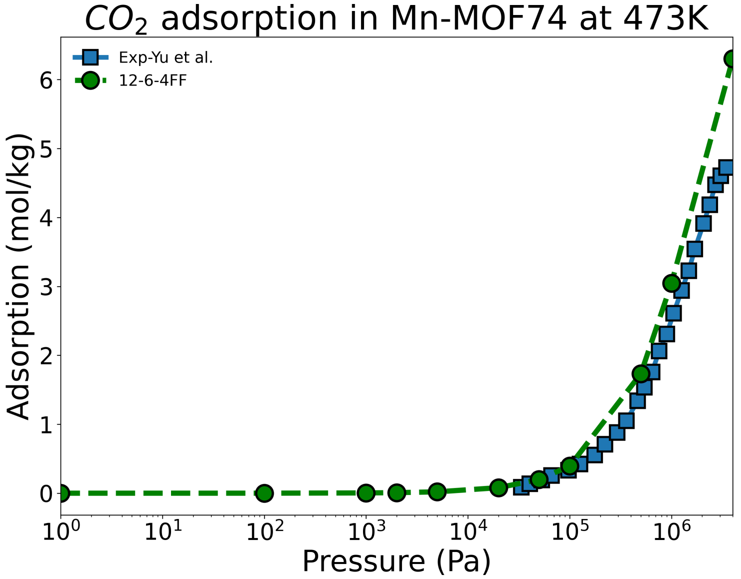

Figure S45: CO_2 adsorption in Mn-MOF-74 at 473 K. Experimental data from Yu *et al.*[24] (squares); fitted 12–6–4 force field shown as green dashed lines.

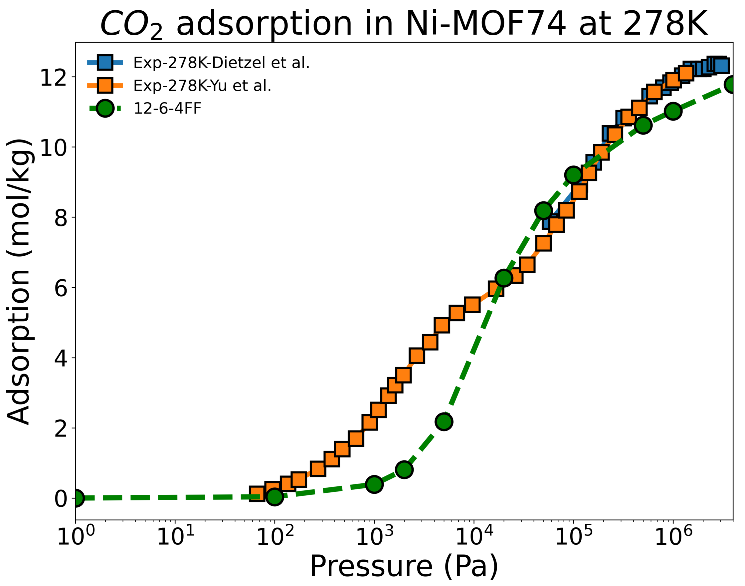

Figure S46: CO_2 adsorption in Ni-MOF-74 at 278 K. Experimental data from Yu *et al.*,[24] Dietzel *et al.*[30] (squares); fitted 12–6–4 force field shown as green dashed lines.

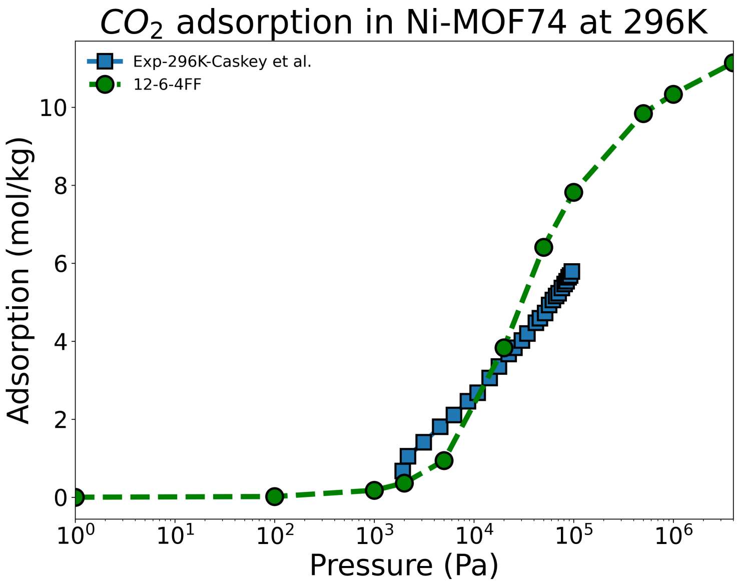

Figure S47: CO_2 adsorption in Ni-MOF-74 at 296 K. Experimental data from Caskey *et al.*[25] (squares); fitted 12–6–4 force field shown as green dashed lines.

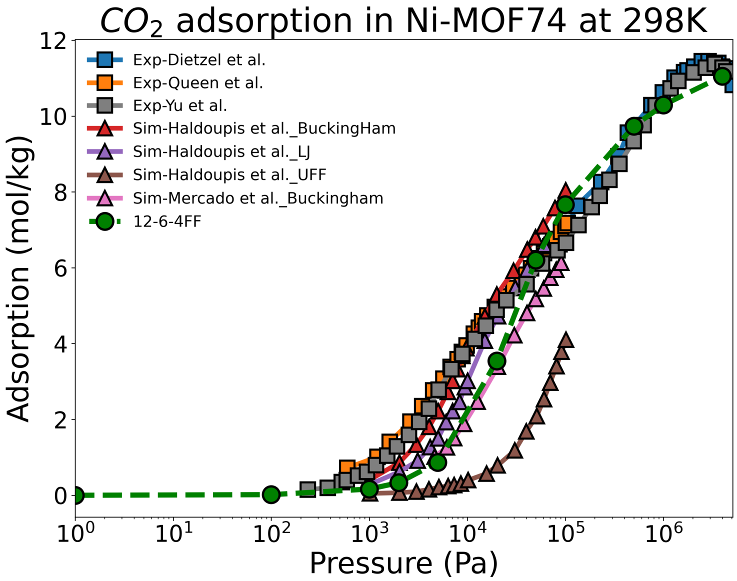

Figure S48: CO_2 adsorption in Ni-MOF-74 at 298 K. Experimental data from Yu *et al.*,[24] Dietzel *et al.*,[30] Queen *et al.*[26] (squares); simulation results from Mercado *et al.*,[27] Haldoupis *et al.*[28] (dashed lines); fitted 12–6–4 force field shown as green dashed lines.

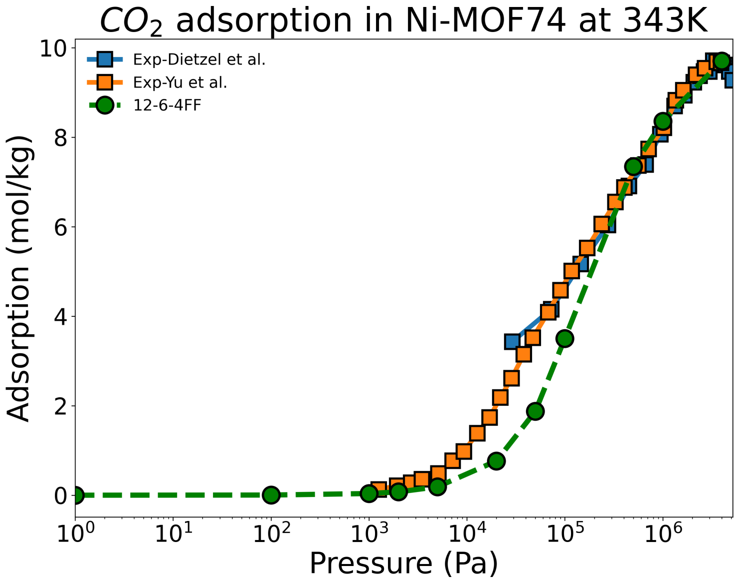

Figure S49: CO_2 adsorption in Ni-MOF-74 at 343 K. Experimental data from Yu *et al.*,[24] Dietzel *et al.*[30] (squares); fitted 12–6–4 force field shown as green dashed lines.

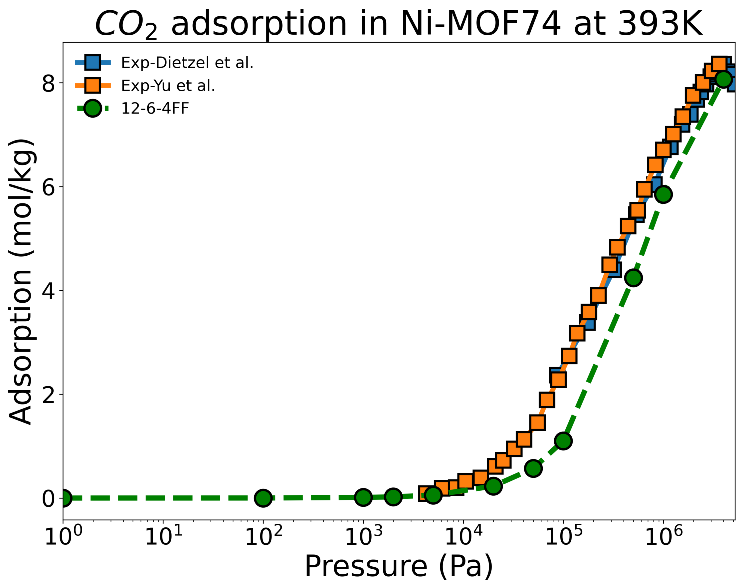

Figure S50: CO_2 adsorption in Ni-MOF-74 at 393 K. Experimental data from Yu *et al.*,[24] Dietzel *et al.*[30] (squares); fitted 12–6–4 force field shown as green dashed lines.

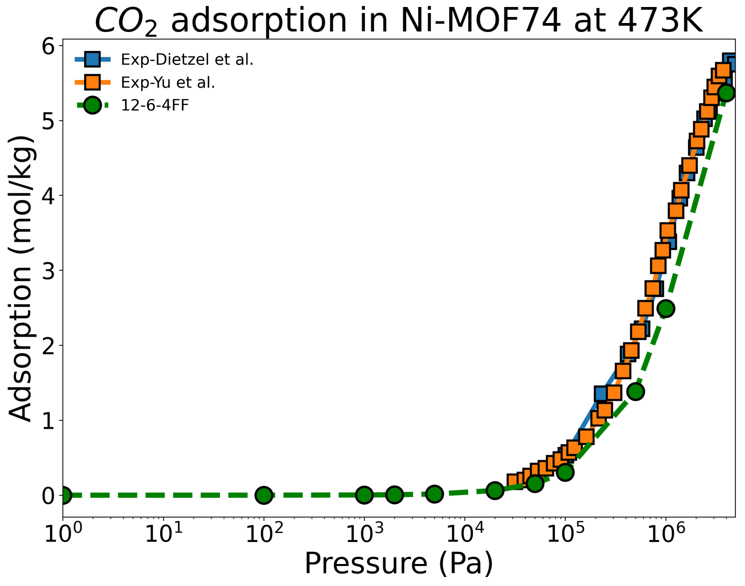

Figure S51: CO_2 adsorption in Ni-MOF-74 at 473 K. Experimental data from Yu *et al.*,[24] Dietzel *et al.*[30] (squares); fitted 12–6–4 force field shown as green dashed lines.

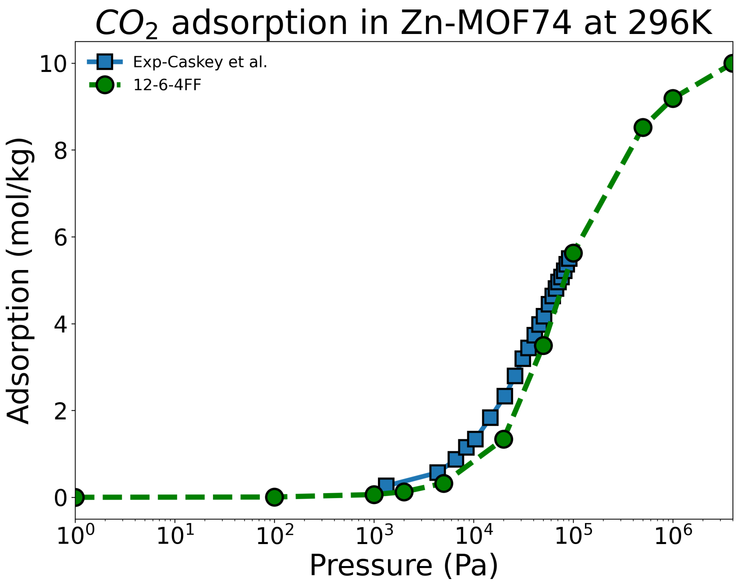

Figure S52: CO_2 adsorption in Zn-MOF-74 at 296 K. Experimental data from Caskey *et al.*[25] (squares); fitted 12–6–4 force field shown as green dashed lines.

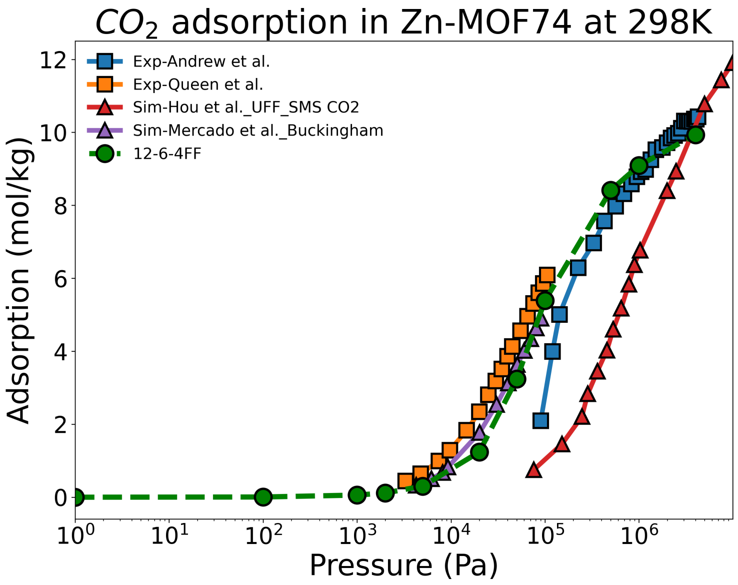

Figure S53: CO_2 adsorption in Zn-MOF-74 at 298 K. Experimental data from Andrew *et al.*,[7] Queen *et al.*[26] (squares); simulation results from Hou *et al.*,[33] Mercado *et al.*[27] (dashed lines); fitted 12–6–4 force field shown as green dashed lines.

H_2O adsorption in M-MOF-74

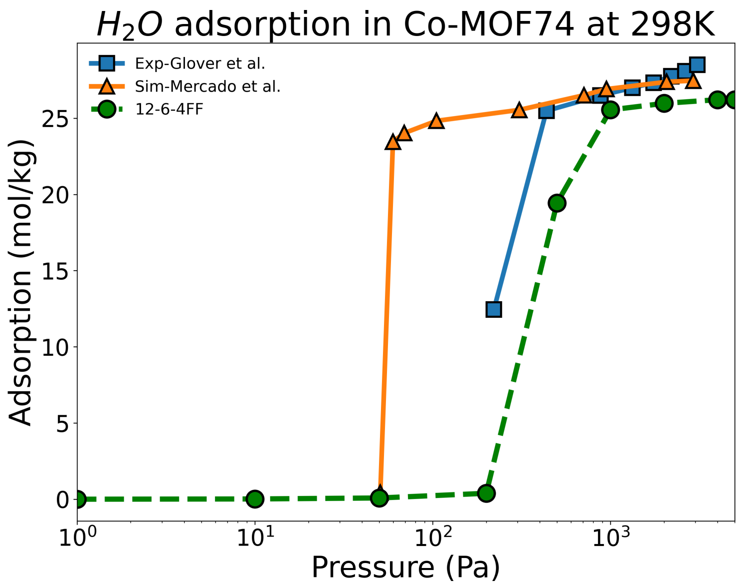

Figure S54: H_2O adsorption in Co-MOF-74 at 298 K. Experimental data from Glover *et al.*,[36] Mercado *et al.*[27] (squares); fitted 12–6–4 force field shown as green dashed lines.

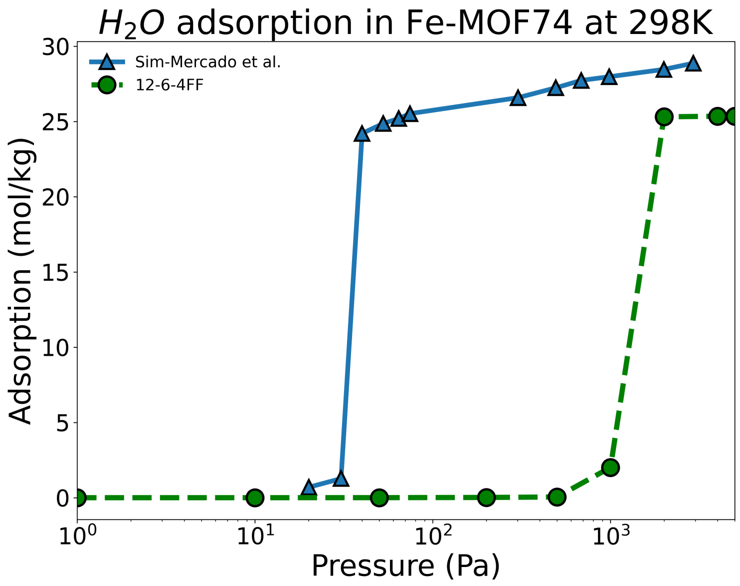

Figure S55: H_2O adsorption in Fe-MOF-74 at 298 K. Simulation results from Mercado *et al.*[27] (dashed lines); fitted 12–6–4 force field shown as green dashed lines.

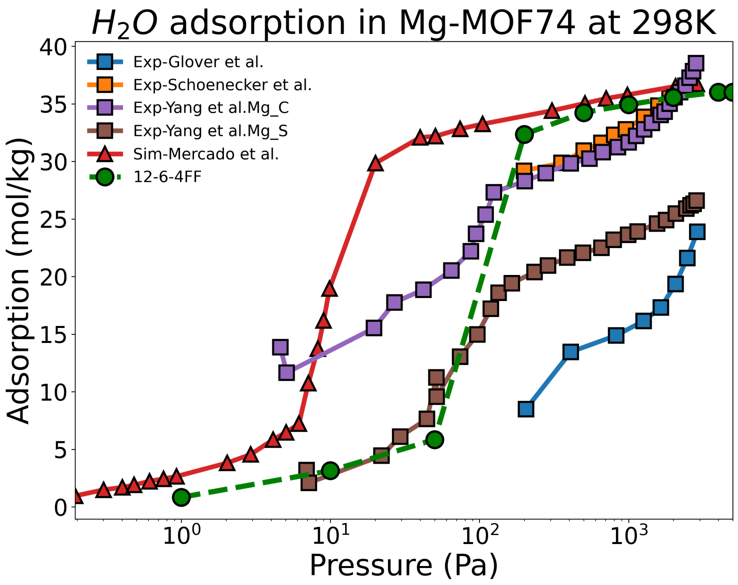

Figure S56: H_2O adsorption in Mg-MOF-74 at 298 K. Experimental data from Glover *et al.*,[36] Schoenecker *et al.*,[37] Yang *et al.*[38] (squares); simulation results from Mercado *et al.*[27] (dashed lines); fitted 12–6–4 force field shown as green dashed lines.

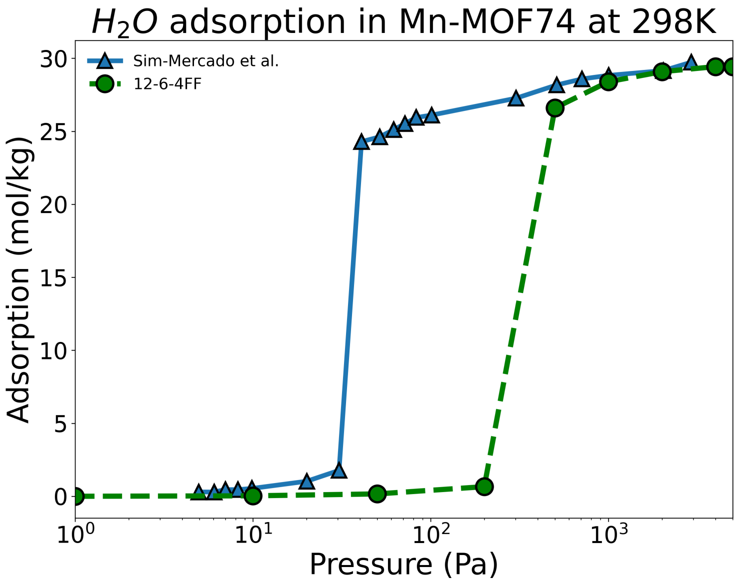

Figure S57: H_2O adsorption in Mn-MOF-74 at 298 K. Simulation results from Mercado *et al.*[27] (dashed lines); fitted 12–6–4 force field shown as green dashed lines.

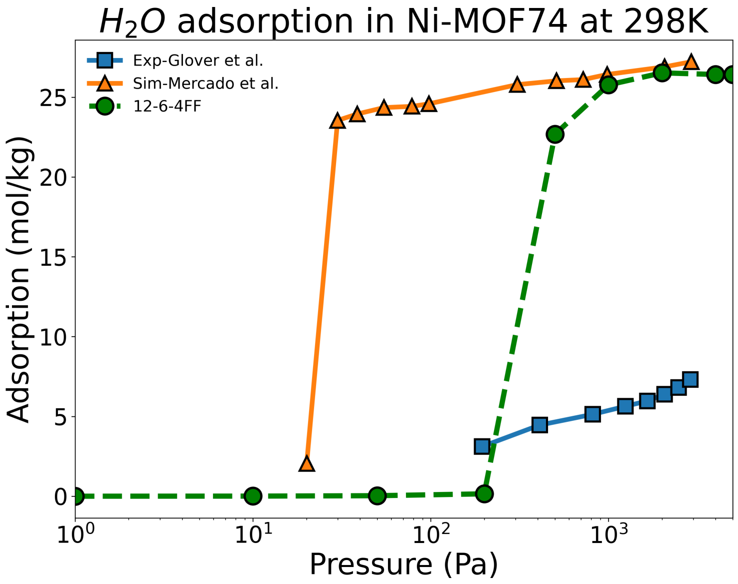

Figure S58: H_2O adsorption in Ni-MOF-74 at 298 K. Experimental data from Glover *et al.*[36] (squares); simulation results from Mercado *et al.*[27] (dashed lines); fitted 12–6–4 force field shown as green dashed lines.

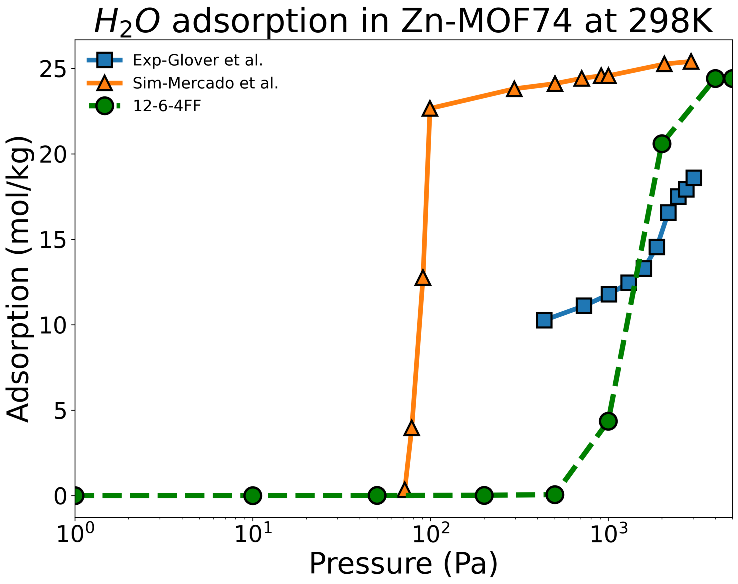

Figure S59: H_2O adsorption in Zn-MOF-74 at 298 K. Experimental data from Glover *et al.*[36] (squares); simulation results from Mercado *et al.*[27] (dashed lines); fitted 12–6–4 force field shown as green dashed lines.

CO_2 adsorption in Cu-BTC

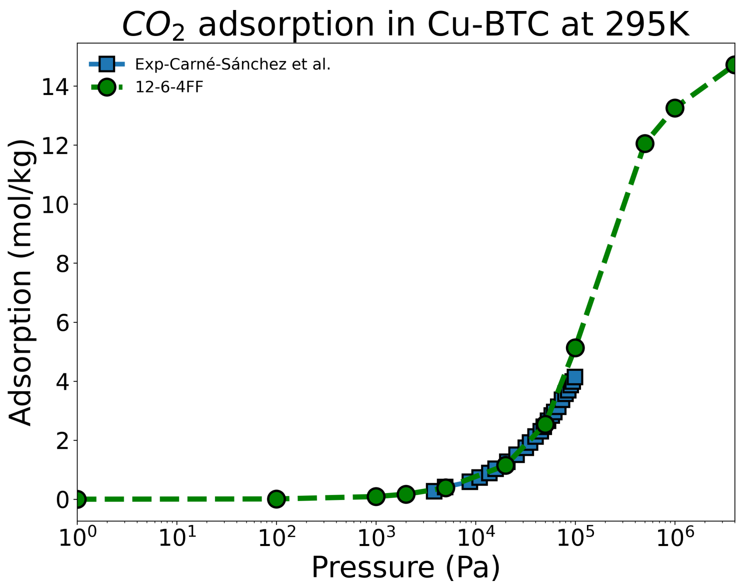

Figure S60: CO_2 adsorption in Cu-BTC at 295 K. Experimental data from Carné-Sánchez *et al.*[39] (squares); fitted 12–6–4 force field shown as green dashed lines.

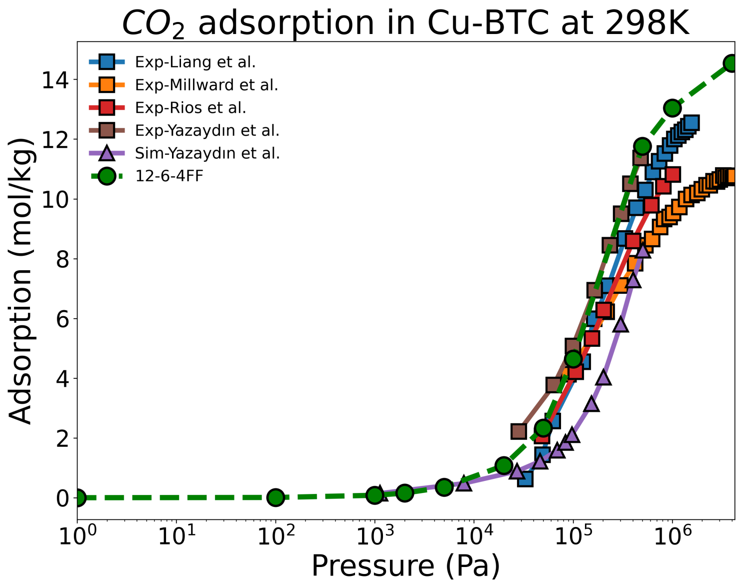

Figure S61: CO_2 adsorption in Cu-BTC at 298 K. Experimental data from Liang *et al.*,[40] Millward *et al.*,[41] Rios *et al.*,[42] Yazaydın *et al.*[43] (squares); simulation results from Yazaydın *et al.*[43] (dashed lines); fitted 12–6–4 force field shown as green dashed lines.

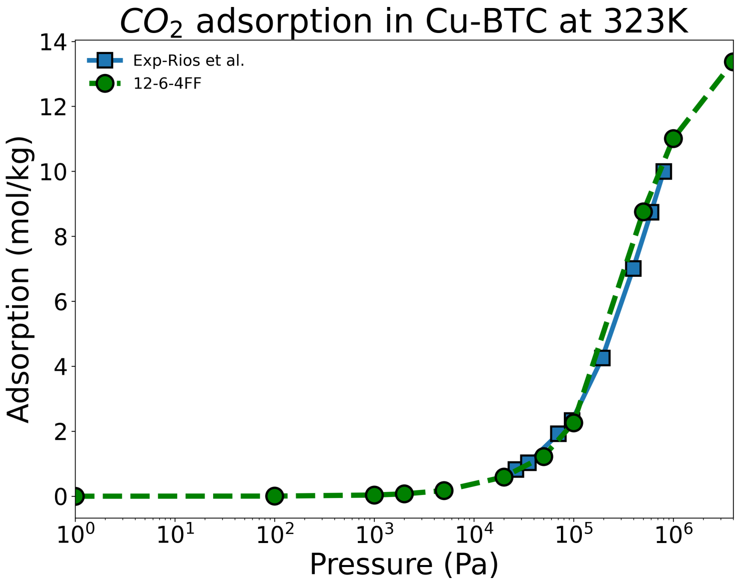

Figure S62: CO_2 adsorption in Cu-BTC at 323 K. Experimental data from Rios *et al.*[42] (squares); fitted 12–6–4 force field shown as green dashed lines.

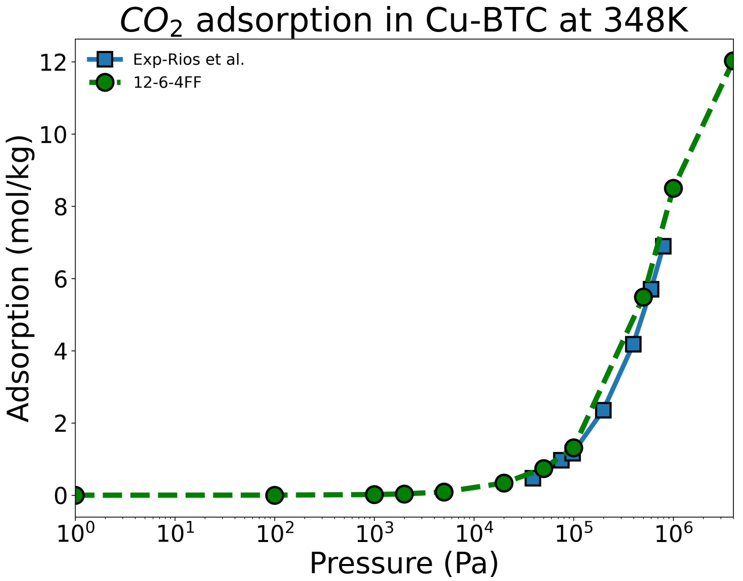

Figure S63: CO_2 adsorption in Cu-BTC at 348 K. Experimental data from Rios *et al.*[42] (squares); fitted 12–6–4 force field shown as green dashed lines.

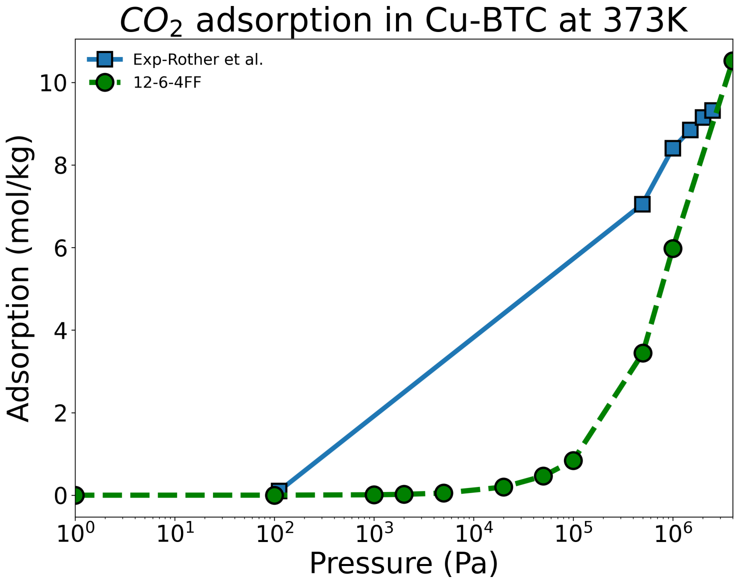

Figure S64: CO_2 adsorption in Cu-BTC at 373 K. Experimental data from Rother *et al.*[44] (squares); fitted 12–6–4 force field shown as green dashed lines.

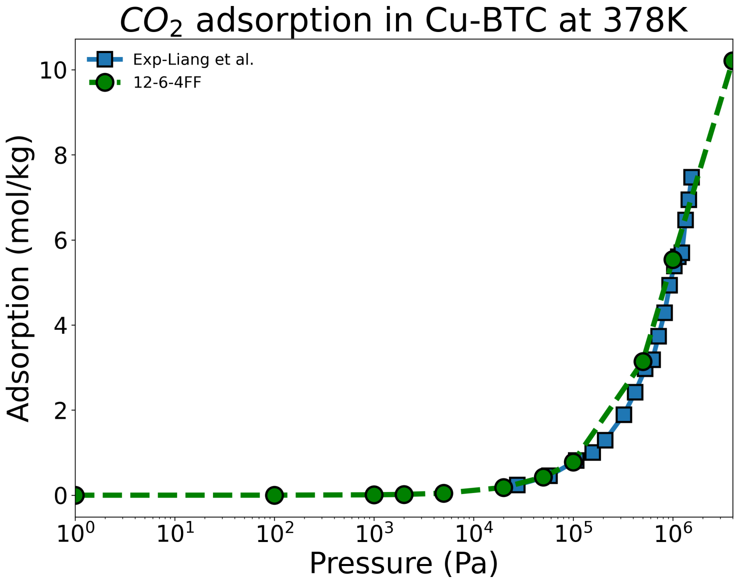

Figure S65: CO_2 adsorption in Cu-BTC at 378 K. Experimental data from Liang *et al.*[40] (squares); fitted 12–6–4 force field shown as green dashed lines.

H_2O adsorption in Cu-BTC

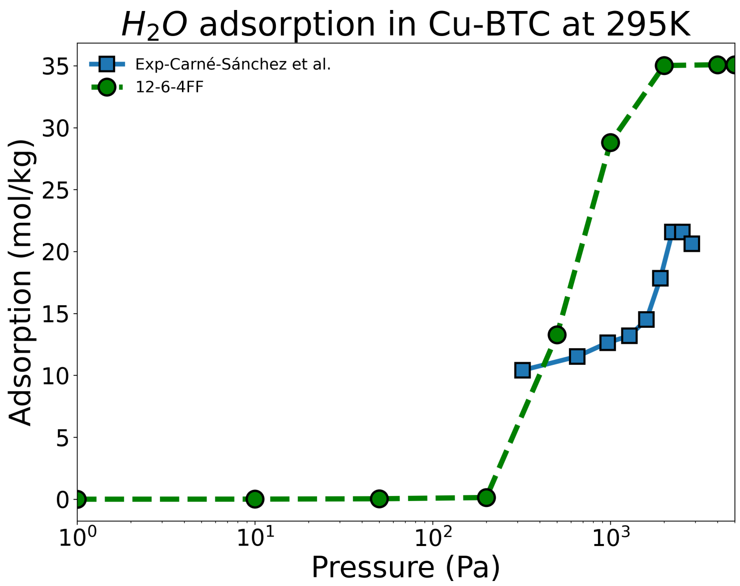

Figure S66: H_2O adsorption in Cu-BTC at 295 K. Experimental data from Carné-Sánchez *et al.*[39] (squares); fitted 12–6–4 force field shown as green dashed lines.

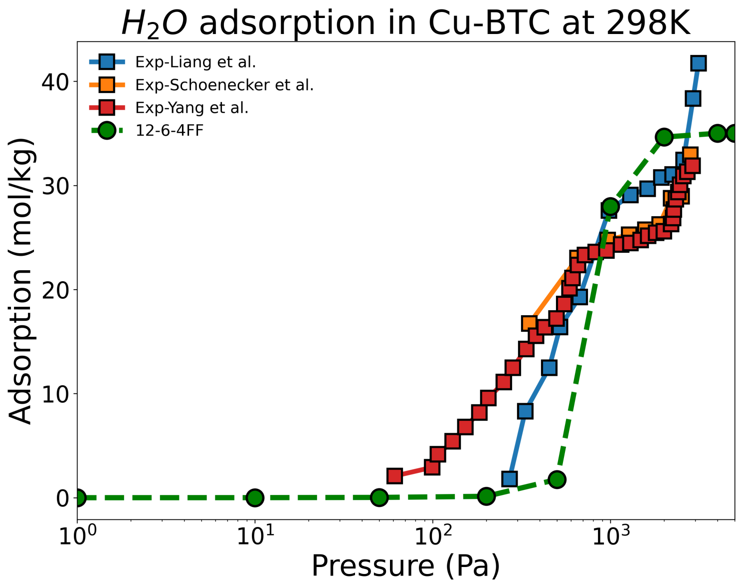

Figure S67: H_2O adsorption in Cu-BTC at 298 K. Experimental data from Liang *et al.*,[40] Schoenecker *et al.*,[37] Yang *et al.*[38] (squares); fitted 12–6–4 force field shown as green dashed lines.

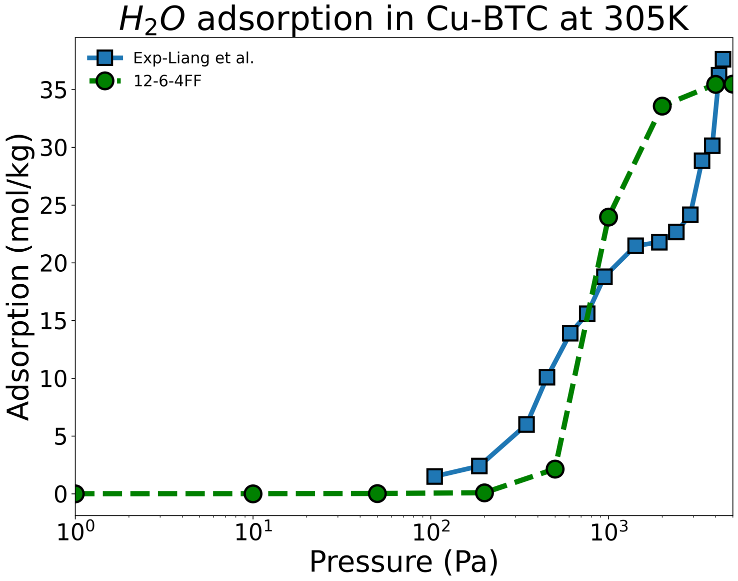

Figure S68: H_2O adsorption in Cu-BTC at 305 K. Experimental data from Liang *et al.*[40] (squares); fitted 12–6–4 force field shown as green dashed lines.

Example Input Files

This section provides representative Gaussian and RASPA input files used for parameter fitting, potential energy surface scan, and GCMC simulations.

Tri-Metal Cluster (TMC) Prototype: $MgAl_2$

The following input corresponds to the $MgAl_2$ cluster model used as a representative prototype for parameterization.

```
%NProcShared=128
#n M06L/def2TZVP em=gd3 pop=chelpg


MgAl2 TMC cluster


0 1
 H   3.42400  -0.36192  -2.61780
 H  -1.32082   3.08247   2.63067
 H  -1.32097   3.08143  -2.63176
 H   3.42430  -0.36125   2.61760
 C   2.68026  -0.29235  -1.80566
 C  -1.61553  -2.08547   1.81780
 C  -1.05422   2.40630   1.79815
 C  -1.05443   2.40561  -1.79893
 C   2.68045  -0.29191   1.80554
 C  -1.61560  -2.08634  -1.81689
 O   2.18778  -1.37700  -1.41078
 O   2.43908   0.88207  -1.39934
 O  -2.34712  -1.13234   1.41607
```

```
O  -0.48029  -2.43507   1.41798
O  -1.98199   1.65330   1.40227
O   0.13306   2.51581   1.38600
O  -1.98214   1.65253  -1.40307
O   0.13270   2.51555  -1.38649
O   2.43926   0.88239   1.39887
O   2.18786  -1.37667   1.41109
O  -2.34724  -1.13318  -1.41531
O  -0.48024  -2.43567  -1.41718
O  -0.07337   0.04940  -0.00012
Al -1.88271   0.18515  -0.00002
Mg  0.78346  -1.71256   0.00026
Al  1.14173   1.43529  -0.00023
H  -1.88128  -2.91326   2.44016
H  -2.14008  -2.60934  -2.57388
```

—

Mg-MOF-74 with CO_2 (Representative PES Scan)

Representative input file of Mg-MOF-74 interacting with CO_2, used for PES scans.

```
%NProcShared=128
#n M06L/def2TZVP Opt=(ReadOpt,modredundant) em=gd3


Mg-MOF74 with CO2 scan


0 1
C   -1.252  -3.548  -2.651
```

C	-2.141	-4.575	-2.910
C	-3.104	-4.918	-1.973
O	3.980	-2.042	-0.085
H	-2.081	-5.112	-3.848
C	-2.189	0.918	1.357
C	2.991	-1.605	0.523
C	0.973	1.260	-1.691
C	-3.174	-4.214	-0.780
C	3.231	-0.871	4.703
C	1.723	5.297	-2.738
C	-3.658	1.158	1.366
C	3.043	-1.263	1.953
C	1.251	2.656	-1.981
C	-2.288	-3.186	-0.513
C	2.217	-0.262	3.983
C	1.553	4.987	-1.408
C	-4.544	0.149	0.989
C	4.081	-1.831	2.698
C	1.467	3.003	-3.325
C	-5.907	0.391	0.949
C	4.182	-1.654	4.060
C	1.688	4.300	-3.716
C	-1.305	-2.844	-1.445
C	2.083	-0.437	2.597
C	1.312	3.665	-0.965
Mg	0.016	-0.793	0.456
Mg	0.484	1.916	1.173

Mg	1.317	-1.487	-1.979
O	-1.773	-0.239	1.060
O	1.259	0.363	-2.540
O	-1.424	1.889	1.620
O	1.843	-1.479	-0.071
O	0.427	0.930	-0.570
O	-0.414	-1.885	-1.184
O	1.079	0.163	1.947
O	1.197	3.434	0.309
H	1.482	0.357	4.483
H	4.799	-2.436	2.159
H	-2.346	-2.630	0.416
H	-4.146	-0.819	0.713
H	1.598	5.756	-0.647
H	1.431	2.206	-4.056
C	-6.408	1.643	1.286
C	-5.530	2.650	1.668
C	-4.166	2.411	1.706
H	-5.910	3.628	1.932
H	3.282	-0.720	5.774
H	4.981	-2.122	4.619
H	-6.583	-0.398	0.647
H	-7.473	1.832	1.251
H	-3.472	3.192	1.989
H	-3.926	-4.471	-0.044
H	-3.798	-5.723	-2.174
H	-0.501	-3.278	-3.390

```
H    1.895   6.329  -3.024

H    1.828   4.545  -4.760

O    2.982  -2.458  -2.430

H    3.584  -2.485  -3.174

H    3.524  -2.374  -1.566

C    0.219  -3.504   1.953

O    0.433  -3.742   0.825

O    0.011  -3.318   3.064


notatoms=3,10,11,44

B 27 62 S 12 -0.1
```

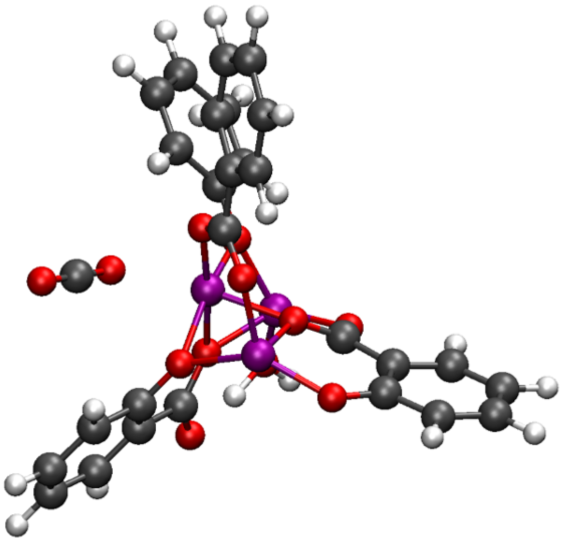

Figure S69: Representative input geometry of CO_2 interacting with Mg-MOF-74 used for potential energy surface (PES) scans, corresponding to the Gaussian input shown above. Color code: gray—C; red—O; white—H; purple—Mg.

—

Cu-BTC with CO_2 (Representative PES Scan)

Representative input file of Cu-BTC interacting with CO_2, used for PES scans.

```
%NProcShared=128
#n M06L/def2TZVP Opt=(readopt,modredundant) em=gd3

Cu-BTC with CO2 scan

0 3
O   1.404  -1.374   1.126
O  -1.404   1.374   1.126
O  -1.404  -1.374   1.126
O   1.404   1.374   1.126
O   1.404   1.374  -1.126
O  -1.404  -1.374  -1.126
O  -1.404   1.374  -1.126
O   1.404  -1.374  -1.126
C   1.853  -1.736   0.000
C  -1.853   1.736   0.000
C  -1.853  -1.736   0.000
C   1.853   1.736   0.000
C  -4.689  -3.893   1.211
C   4.689  -3.893  -1.211
C   4.689  -3.893   1.211
C  -4.689  -3.893  -1.211
C   3.021  -2.653   0.000
C  -3.021   2.653   0.000
C   4.689   3.893  -1.211
C  -3.021  -2.653   0.000
C   3.021   2.653   0.000
```

C	-4.689	3.893	-1.211
C	-4.689	3.893	1.211
C	4.689	3.893	1.211
C	-3.578	-3.068	1.207
C	3.578	-3.068	-1.207
C	3.578	-3.068	1.207
C	-3.578	-3.068	-1.207
C	-3.578	3.068	1.207
C	-5.242	-4.303	0.000
C	5.242	4.303	0.000
C	3.578	3.068	1.207
C	5.242	-4.303	0.000
C	-5.242	4.303	0.000
C	3.578	3.068	-1.207
C	-3.578	3.068	-1.207
H	-3.129	-2.728	2.131
H	3.129	-2.728	-2.131
H	3.129	-2.728	2.131
H	-3.129	-2.728	-2.131
H	-3.129	2.728	2.131
H	-6.112	-4.947	0.000
H	6.112	4.947	0.000
H	3.129	2.728	2.131
H	6.112	-4.947	0.000
H	-6.112	4.947	0.000
H	3.129	2.728	-2.131
H	-3.129	2.728	-2.131

```
H  -5.127  -4.217   2.146

H  -5.127  -4.217  -2.146

H   5.127  -4.217   2.146

H   5.127  -4.217  -2.146

H   5.127   4.217   2.146

H   5.127   4.217  -2.146

H  -5.127   4.217   2.146

H  -5.127   4.217  -2.146

Cu   0.000   0.000  -1.234

Cu   0.000   0.000   1.234

C  -0.088  -0.817   4.431

O   0.238  -0.001   3.696

O  -0.422  -1.619   5.220


notatoms=1-58

B 58 60 S 9 -0.1
```

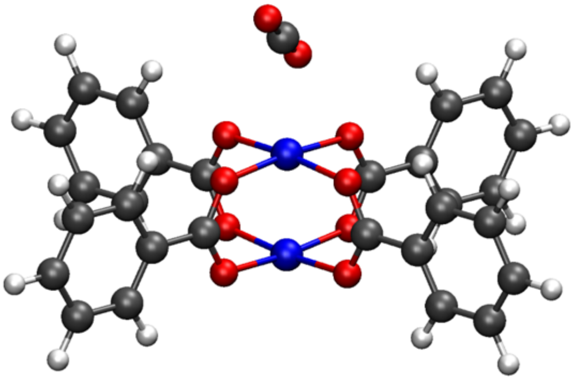

Figure S70: Representative input geometry of CO_2 interacting with Cu-BTC used for potential energy surface (PES) scans, corresponding to the Gaussian input shown above. Color code: gray—C; red—O; white—H; blue—Cu.

—

GCMC

Mg-MOF-74 (CO_2, 298 K, 100 kPa)

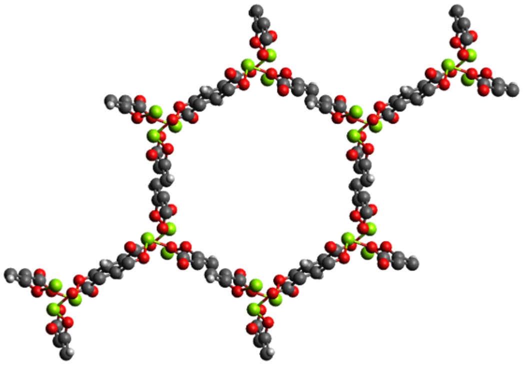

Figure S71: Crystal structure of Mg-MOF-74 used in GCMC simulations, corresponding to the supercell defined in `simulation.input` (4 × 2 × 2). Color code: gray—C; red—O; white—H; green—Mg.

Representative input files used for the GCMC simulation of CO_2 adsorption in Mg-MOF-74 at 298 K and 100 kPa are shown below. The simulation was carried out using the RASPA2 package with local force field definitions.

simulation.input

```
SimulationType         MonteCarlo
NumberOfCycles         100000
NumberOfInitializationCycles 10000
PrintEvery                     1000
WriteBinaryRestartFileEvery    1000
ContinueAfterCrash yes


Forcefield                          local
UseChargesFromCIFFile               yes
ChargeMethod                        Ewald
```

```
EwaldPrecision                    1e-6
CutOff                            12.8
CutOffVDW                         12.8

Framework 0
FrameworkName qmof-aa922ec
UnitCells 4 2 2
ExternalTemperature 298.0
ExternalPressure 100000

Movies yes
WriteMoviesEvery 5000

Component 0 MoleculeName             CO2
            MoleculeDefinition       local
            MolFraction              1.0
            TranslationProbability   0.5
            RotationProbability      0.5
            ReinsertionProbability   0.5
            SwapProbability          1.0
            CreateNumberOfMolecules  0
```

CO2.def

```
# critical constants: Temperature [T], Pressure [Pa], and Acentric factor [-]
304.1282
7377300.0
```

```
0.22394
# total number Of atoms
3
# number of groups
1
# water-group
rigid
# number of atoms
3
# atomic positions
0 C_CO2       -4.263152   -0.264102   0.403237
1 O_CO2       -4.760725   -0.284676   1.445173
2 O_CO2       -3.791769   -0.249455         -0.661392
# Chiral centers Bond  BondDipoles Bend  UrayBradley InvBend  Torsion Imp. Torsion
Bond/Bond Stretch/Bend Bend/Bend Stretch/Torsion Bend/Torsion IntraVDW IntraCoulomb
     0    2    0    0      0   0     0     0    0   0    0      0      0  0   0
# Bond stretch: atom n1-n2, type, parameters
0 1 RIGID_BOND
0 2 RIGID_BOND
# Number of config moves
0
```

forcefield.def

```
# rules to overwrite
0
# number of defined interactions
```

```
1
# type      type2       interaction
Mg_  O_CO2         GENERIC2_HC   0  0    18656     5716      0   -12209018
# mixing rules to overwrite
0
```

force_field_mixing_rules.def

```
# general rule for shifted vs truncated
truncated
# general rule tailcorrections
no
# number of defined interactions
27
# type interaction
O_             lennard-jones    48.1581   3.03315      // Dreiding
C_             lennard-jones    47.8562   3.47299      // Dreiding
H_             lennard-jones    7.64893  2.84642      // Dreiding
Cu_            lennard-jones    2.5161   3.11369      // UFF
Co_            lennard-jones    7.04507  2.55866      // UFF
Fe_            lennard-jones    6.54185  2.5943      // UFF
Mg_            lennard-jones    55.8574   2.69141      // UFF
Mn_            lennard-jones    6.54185  2.63795      // UFF
Ni_            lennard-jones    7.54829  2.52481      // UFF
Zn_            lennard-jones    62.3992   2.46155      // UFF
OwH2O_TIP3P    lennard-jones    76.53   3.15      // TIP3P
HwH2O_TIP3P    none                              // none
```

```
OwH2O_TIP4P    lennard-jones    78.0     3.154      // TIP4P
HwH2O_TIP4P    none                                 // none
MwH2O_TIP4P    none                                 // none
OwH2O_TIP4PEW    lennard-jones    81.9     3.164      // TIP4PEW
HwH2O_TIP4PEW    none                                 // none
MwH2O_TIP4PEW    none                                 // none
OwH2O_TIP5P    lennard-jones    80.52    3.12      // TIP5P
HwH2O_TIP5P    none                                 // none
MwH2O_TIP5P    none                                 // none
OwH2O_TIP5PEW    lennard-jones    89.57     3.097     // TIP5PEW
HwH2O_TIP5PEW    none                                 // none
MwH2O_TIP5PEW    none                                 // none
C_CO2  lennard-jones    27.0    2.8      // trap
O_CO2     lennard-jones    79.0    3.05      // trap
CH2      lennard-jones    85.0     3.675      // trap
# general mixing rule for Lennard-Jones
Lorentz-Berthelot
```

pseudo_atoms.def

```
#number of pseudo atoms
25
#type       print   as    chem  oxidation   mass        charge   polarization
B-factor radii  connectivity anisotropic anisotropic-type   tinker-type
O_CO2 yes O O 0 15.9996 -0.350 0.0 1.0 1.0 1 0 absolute 0
C_CO2 yes C C 0 12.0110 0.700 0.0 1.0 1.0 2 0 absolute 0
CH2 yes C C 0 14.0126 0.000 0.0 1.0 1.0 1 0 absolute 0
```

```
Mg yes Mg Mg 0 24.3050 -0.834 0.0 1.0 1.0 5 0 absolute 0
Cu yes Cu Cu 0 63.5460 1.080 0.0 1.0 1.0 5 0 absolute 0
Co yes Co Co 0 58.9330 0.944 0.0 1.0 1.0 5 0 absolute 0
Fe yes Fe Fe 0 55.8450 1.090 0.0 1.0 1.0 5 0 absolute 0
Mn yes Mn Mn 0 54.9380 1.232 0.0 1.0 1.0 5 0 absolute 0
Ni yes Ni Ni 0 58.6930 0.915 0.0 1.0 1.0 5 0 absolute 0
Zn yes Zn Zn 0 65.3800 1.171 0.0 1.0 1.0 5 0 absolute 0
C yes C C 0 12.0110 0.417 0.0 1.0 1.0 3 0 absolute 0
O yes O O 0 15.9996 -0.834 0.0 1.0 1.0 3 0 absolute 0
H yes H H 0 1.0008 0.417 0.0 1.0 1.0 1 0 absolute 0
OwH2O_TIP4P yes O O 0 15.9996 0.000 0.0 1.0 1.0 3 0 absolute 0
HwH2O_TIP4P yes H H 0 1.0008 0.520 0.0 1.0 1.0 1 0 absolute 0
MwH2O_TIP4P yes M - 0 0.000 -1.040 0.0 1.0 1.0 1 0 absolute 0
OwH2O_TIP4PEW yes O O 0 15.9996 0.000 0.0 1.0 1.0 3 0 absolute 0
HwH2O_TIP4PEW yes H H 0 1.0008 0.52422 0.0 1.0 1.0 1 0 absolute 0
MwH2O_TIP4PEW yes M - 0 0.000 -1.04844 0.0 1.0 1.0 1 0 absolute 0
OwH2O_TIP5P yes O O 0 15.9996 0.000 0.0 1.0 1.0 3 0 absolute 0
HwH2O_TIP5P yes H H 0 1.0008 0.241 0.0 1.0 1.0 1 0 absolute 0
MwH2O_TIP5P yes M - 0 0.000 -0.241 0.0 1.0 1.0 1 0 absolute 0
OwH2O_TIP5PEW yes O O 0 15.9996 0.000 0.0 1.0 1.0 3 0 absolute 0
HwH2O_TIP5PEW yes H H 0 1.0008 0.241 0.0 1.0 1.0 1 0 absolute 0
MwH2O_TIP5PEW yes M - 0 0.000 -0.241 0.0 1.0 1.0 1 0 absolute 0
```

qmof-aa922ec.cif

```
#
data_MgHC4O3
```

_symmetry_space_group_name_H-M   'P 1'

_cell_length_a   6.88935740

_cell_length_b   15.23153998

_cell_length_c   15.23233797

_cell_angle_alpha   117.77089256

_cell_angle_beta   98.67768450

_cell_angle_gamma   98.67432658

_symmetry_Int_Tables_number   1

_chemical_formula_structural   MgHC4O3

_chemical_formula_sum 'Mg6

_cell_volume   1352.70389140

_cell_formula_units_Z   6

loop_

_symmetry_equiv_pos_site_id

_symmetry_equiv_pos_as_xyz

1

loop_

_atom_site_type_symbol

_atom_site_label

_atom_site_symmetry_multiplicity

_atom_site_fract_x

_atom_site_fract_y

_atom_site_fract_z

_atom_site_charge

Mg Mg0 1 0.01751067 0.73176571 0.40605260 1.406928

Mg Mg1 1 0.28580893 0.67424527 0.26821473 1.407919

Mg Mg2 1 0.61142624 0.59391716 0.32573987 1.408025

Mg	Mg3	1	0.98248950	0.26823409	0.59394701	1.406924
Mg	Mg4	1	0.71419309	0.32575530	0.73178536	1.407853
Mg	Mg5	1	0.38857392	0.40608263	0.67425974	1.408036
H	H6	1	0.68595139	0.55475504	0.53273334	0.132848
H	H7	1	0.13114625	0.97795544	0.44523675	0.132742
H	H8	1	0.15319564	0.46718725	0.02203476	0.132422
H	H9	1	0.31404839	0.44524399	0.46726588	0.132849
H	H10	1	0.86885430	0.02204512	0.55476324	0.132723
H	H11	1	0.84680600	0.53281254	0.97796495	0.132393
C	C12	1	0.93311690	0.56646853	0.46543479	0.336357
C	C13	1	0.36662573	0.89893238	0.43351556	0.334591
C	C14	1	0.46772935	0.53451974	0.10102429	0.335749
C	C15	1	0.06687995	0.43353050	0.53456488	0.336332
C	C16	1	0.63337443	0.10106742	0.56648404	0.334567
C	C17	1	0.53227082	0.46548006	0.89897466	0.335773
C	C18	1	0.24291357	0.56396701	0.39109208	0.678806
C	C19	1	0.67898850	0.82711247	0.43603325	0.678709
C	C20	1	0.85190751	0.60894184	0.17293386	0.675981
C	C21	1	0.75708698	0.43603355	0.60890856	0.678787
C	C22	1	0.32101167	0.17288733	0.56396636	0.678802
C	C23	1	0.14809304	0.39105872	0.82706613	0.676041
C	C24	1	0.82434757	0.53084159	0.51796305	-0.204058
C	C25	1	0.29347483	0.98710876	0.46914056	-0.204362
C	C26	1	0.30635596	0.48195067	0.01287385	-0.20423
C	C27	1	0.17564927	0.46915744	0.48203729	-0.20401
C	C28	1	0.70652533	0.01289103	0.53085970	-0.204272
C	C29	1	0.69364382	0.51804836	0.98712538	-0.204213

C	C30	1	0.11488902	0.53377751	0.44730610	-0.164125
C	C31	1	0.58113561	0.91353864	0.46621874	-0.162848
C	C32	1	0.66766261	0.55271174	0.08648212	-0.162958
C	C33	1	0.88510967	0.46622229	0.55269406	-0.164111
C	C34	1	0.41886455	0.08646115	0.53378087	-0.162813
C	C35	1	0.33233756	0.44728806	0.91351749	-0.16299
O	O36	1	0.86170774	0.62836823	0.43504624	-0.790472
O	O37	1	0.23332557	0.80663039	0.37162656	-0.789549
O	O38	1	0.42673133	0.56492705	0.19332382	-0.790621
O	O39	1	0.13829242	0.37163156	0.56495468	-0.790477
O	O40	1	0.76667460	0.19336941	0.62837305	-0.789525
O	O41	1	0.57326884	0.43507274	0.80667513	-0.790585
O	O42	1	0.19152133	0.62619945	0.36173066	-0.761903
O	O43	1	0.56534542	0.73552585	0.37378324	-0.762702
O	O44	1	0.82976162	0.63828531	0.26450067	-0.760949
O	O45	1	0.80847922	0.37380111	0.63826933	-0.761893
O	O46	1	0.43465474	0.26447395	0.62621636	-0.762743
O	O47	1	0.17024001	0.36171448	0.73549838	-0.760959
O	O48	1	0.39897925	0.53052627	0.37272284	-0.634872
O	O49	1	0.86848910	0.84217884	0.46948321	-0.634336
O	O50	1	0.02637484	0.62732051	0.15790226	-0.633147
O	O51	1	0.60102091	0.46947352	0.62727677	-0.634865
O	O52	1	0.13151106	0.15782096	0.53051640	-0.634393
O	O53	1	0.97362571	0.37268005	0.84209708	-0.63318

Cu-BTC (CO_2, 298 K, 100 kPa)

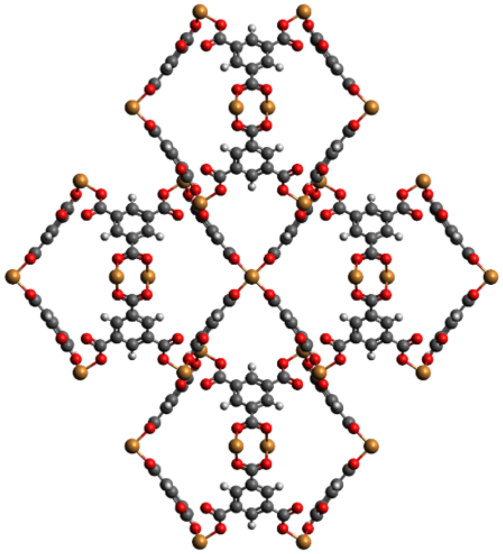

Figure S72: Crystal structure of Cu-BTC used in GCMC simulations, corresponding to the supercell defined in `simulation.input` (2 × 2 × 2). Color code: gray—C; red—O; white—H; brown—Cu.

Representative input files used for the GCMC simulation of CO_2 adsorption in Cu-BTC at 298 K and 100 kPa are shown below. Except for the framework file (`qmof-8b5bb88.cif`), the framework-specific parameters in `forcefield.def`, and the unit cell settings in `simulation.input`, the remaining definitions (`CO2.def`, `force_field_mixing_rules.def`, and `pseudo_atoms.def`) are identical to those used for Mg-MOF-74.

simulation.input

```
SimulationType         MonteCarlo

NumberOfCycles         100000

NumberOfInitializationCycles 10000

PrintEvery             1000

WriteBinaryRestartFileEvery 1000

ContinueAfterCrash      yes
```

```
Forcefield              local
UseChargesFromCIFFile   yes
ChargeMethod            Ewald
EwaldPrecision          1e-6
CutOff                  12.8
CutOffVDW               12.8

Framework 0
FrameworkName           qmof-8b5bb88
UnitCells               2 2 2
ExternalTemperature     298.0
ExternalPressure        100000

Movies yes
WriteMoviesEvery        5000

Component 0 MoleculeName            CO2
            MoleculeDefinition      local
            MolFraction             1.0
            TranslationProbability  0.5
            RotationProbability     0.5
            ReinsertionProbability  0.5
            SwapProbability         1.0
            CreateNumberOfMolecules 0
```

forcefield.def

```
# rules to overwrite
```

```
0
# number of defined interactions
1
# type      type2       interaction
Cu_  O_CO2         GENERIC2_HC   0  0    18031     7702      0   -11506934
# mixing rules to overwrite
0
```

qmof-8b5bb88.cif

```
#
data_CuH2(C3O2)2
_symmetry_space_group_name_H-M   'P 1'
_cell_length_a   18.72306011
_cell_length_b   18.71893229
_cell_length_c   18.71683659
_cell_angle_alpha   60.01879442
_cell_angle_beta   60.00353360
_cell_angle_gamma   59.98513483
_symmetry_Int_Tables_number   1
_chemical_formula_structural   CuH2(C3O2)2
_chemical_formula_sum  'Cu12
_cell_volume   4638.73725646
_cell_formula_units_Z   12
loop_
_symmetry_equiv_pos_site_id
_symmetry_equiv_pos_as_xyz
```

```
1
loop_
_atom_site_type_symbol
_atom_site_label
_atom_site_symmetry_multiplicity
_atom_site_fract_x
_atom_site_fract_y
_atom_site_fract_z
_atom_site_charge
Cu  Cu0  1  0.43437784  0.00001315  0.56562364  0.837056
Cu  Cu1  1  0.99999052  0.56563385  0.00001292  0.836485
Cu  Cu2  1  0.56561115  0.99998785  0.43439108  0.836772
Cu  Cu3  1  0.00000766  0.43434145  0.99998954  0.836782
Cu  Cu4  1  0.43437418  0.00000396  0.99999739  0.837149
Cu  Cu5  1  0.00000221  0.43435376  0.56565269  0.837125
Cu  Cu6  1  0.99998921  0.00000263  0.56568309  0.837356
Cu  Cu7  1  0.43435597  0.56563663  0.00001040  0.836646
Cu  Cu8  1  0.99999469  0.56560162  0.43439498  0.836815
Cu  Cu9  1  0.56564440  0.43435987  0.99999289  0.836699
Cu  Cu10  1  0.56561180  0.99999479  0.00000187  0.83701
Cu  Cu11  1  0.00000624  0.99999648  0.43437521  0.836578
H  H12  1  0.49629287  0.26606279  0.26617052  0.134413
H  H13  1  0.02853958  0.50368852  0.73383429  0.134547
H  H14  1  0.26616903  0.97147068  0.49629281  0.134467
H  H15  1  0.73384014  0.73393919  0.02854147  0.134449
H  H16  1  0.73381706  0.02852791  0.73392722  0.134434
H  H17  1  0.26611107  0.49627199  0.97149555  0.134449
```

H	H18	1	0.02853947	0.73383931	0.50365864	0.134389
H	H19	1	0.73383317	0.02852519	0.50370140	0.13438
H	H20	1	0.97149473	0.26612276	0.26610942	0.134441
H	H21	1	0.73388982	0.50372853	0.02850862	0.134494
H	H22	1	0.49633782	0.26604343	0.97146131	0.134442
H	H23	1	0.49634409	0.97143608	0.26610539	0.134574
H	H24	1	0.26610253	0.26612015	0.49634092	0.134606
H	H25	1	0.26606529	0.49627725	0.26618816	0.134389
H	H26	1	0.26616622	0.26605955	0.97146165	0.134563
H	H27	1	0.97146058	0.26616150	0.49633141	0.134458
H	H28	1	0.26618910	0.97146926	0.26606311	0.134416
H	H29	1	0.97145928	0.49631705	0.26616578	0.134547
H	H30	1	0.73390001	0.73388297	0.50365351	0.134536
H	H31	1	0.50366166	0.02856087	0.73389772	0.134636
H	H32	1	0.73393268	0.50372766	0.73381167	0.134385
H	H33	1	0.50366705	0.73395686	0.02854002	0.134415
H	H34	1	0.02850734	0.73387331	0.73388826	0.134503
H	H35	1	0.50370778	0.73393406	0.73382963	0.134467
C	C36	1	0.38638189	0.25700465	0.38643011	-0.030614
C	C37	1	0.02979244	0.61359692	0.74297616	-0.030667
C	C38	1	0.38642595	0.97018298	0.38638537	-0.030461
C	C39	1	0.74298004	0.61363677	0.02979479	-0.030697
C	C40	1	0.61357061	0.02981548	0.74299309	-0.030653
C	C41	1	0.25702189	0.38636755	0.97020940	-0.030644
C	C42	1	0.02980195	0.61359879	0.61357516	-0.031123
C	C43	1	0.74295198	0.02980885	0.61360719	-0.030522
C	C44	1	0.97020895	0.38640735	0.25701857	-0.030689

C	C45	1	0.61358833	0.61363789	0.02979275	-0.030484
C	C46	1	0.38641779	0.38635844	0.97021020	-0.030579
C	C47	1	0.38643560	0.97018093	0.25700375	-0.031077
C	C48	1	0.25700479	0.38638527	0.38643008	-0.030502
C	C49	1	0.38638707	0.38637191	0.25704841	-0.030425
C	C50	1	0.38641989	0.25697598	0.97020007	-0.031219
C	C51	1	0.97020686	0.25702006	0.38640908	-0.030689
C	C52	1	0.25704937	0.97018920	0.38638073	-0.030413
C	C53	1	0.97019917	0.38640791	0.38641506	-0.031163
C	C54	1	0.61361504	0.74299746	0.61357098	-0.03059
C	C55	1	0.61357370	0.02981518	0.61361407	-0.03061
C	C56	1	0.74299538	0.61362720	0.61355867	-0.030952
C	C57	1	0.61358162	0.74302406	0.02980220	-0.031073
C	C58	1	0.02979402	0.74298203	0.61357944	-0.030683
C	C59	1	0.61361784	0.61362413	0.74294940	-0.030346
C	C60	1	0.43056557	0.16297834	0.43059944	0.58281
C	C61	1	0.02417442	0.56940235	0.83699520	0.582573
C	C62	1	0.43059330	0.97586044	0.43056918	0.582923
C	C63	1	0.83699811	0.56943070	0.02417779	0.582689
C	C64	1	0.56939982	0.02412856	0.83702583	0.583065
C	C65	1	0.16300312	0.43057509	0.97582343	0.582443
C	C66	1	0.02412834	0.56941815	0.56939964	0.583151
C	C67	1	0.83696121	0.02418412	0.56941951	0.582773
C	C68	1	0.97582224	0.43060006	0.16300494	0.582496
C	C69	1	0.56939262	0.56942510	0.02418780	0.582466
C	C70	1	0.43061590	0.43056730	0.97581446	0.582562
C	C71	1	0.43059999	0.97586873	0.16297274	0.583394

C	C72	1	0.16297313	0.43056179	0.43059597	0.582984
C	C73	1	0.43058478	0.43056263	0.16303716	0.582501
C	C74	1	0.43059882	0.16294386	0.97586692	0.583561
C	C75	1	0.97581152	0.16301005	0.43061185	0.582625
C	C76	1	0.16303804	0.97581658	0.43057866	0.582479
C	C77	1	0.97586870	0.43058449	0.43059903	0.583597
C	C78	1	0.56943208	0.83702335	0.56940506	0.582813
C	C79	1	0.56940118	0.02414117	0.56943603	0.582875
C	C80	1	0.83702572	0.56944698	0.56939821	0.58286
C	C81	1	0.56940004	0.83705611	0.02413202	0.583538
C	C82	1	0.02418230	0.83699114	0.56939454	0.582649
C	C83	1	0.56942452	0.56942458	0.83696300	0.582227
C	C84	1	0.42965959	0.30009679	0.30016914	-0.075545
C	C85	1	0.02992597	0.57031305	0.69984481	-0.075031
C	C86	1	0.30016654	0.97007154	0.42965800	-0.075737
C	C87	1	0.69984949	0.69991897	0.02992723	-0.075013
C	C88	1	0.69982737	0.02992783	0.69989071	-0.075507
C	C89	1	0.30013505	0.42962809	0.97010875	-0.075508
C	C90	1	0.02992590	0.69985603	0.57029211	-0.075045
C	C91	1	0.69983249	0.02992559	0.57033681	-0.075408
C	C92	1	0.97010725	0.30013465	0.30012862	-0.075515
C	C93	1	0.69987004	0.57037230	0.02989550	-0.075683
C	C94	1	0.42970427	0.30007487	0.97007755	-0.075107
C	C95	1	0.42972617	0.97004050	0.30011556	-0.07528
C	C96	1	0.30011304	0.30012268	0.42972427	-0.075556
C	C97	1	0.30010676	0.42965018	0.30017305	-0.075435
C	C98	1	0.30015208	0.30008438	0.97007644	-0.075123

C	C99	1	0.97007560	0.30014625	0.42970073	-0.075102
C	C100	1	0.30017943	0.97006935	0.30010088	-0.075283
C	C101	1	0.97007486	0.42969070	0.30015048	-0.075086
C	C102	1	0.69988355	0.69988493	0.57027165	-0.075171
C	C103	1	0.57027895	0.02995672	0.69988414	-0.075507
C	C104	1	0.69989976	0.57035226	0.69981960	-0.075248
C	C105	1	0.57030058	0.69992240	0.02992465	-0.075193
C	C106	1	0.02989559	0.69986828	0.69986386	-0.075594
C	C107	1	0.57034209	0.69989866	0.69983230	-0.075705
O	O108	1	0.38627122	0.12646888	0.50814576	-0.515027
O	O109	1	0.02092083	0.61370924	0.87353099	-0.514455
O	O110	1	0.50813814	0.97912407	0.38627429	-0.515012
O	O111	1	0.87352489	0.49184126	0.02092460	-0.514579
O	O112	1	0.49185843	0.02086089	0.87354300	-0.515109
O	O113	1	0.12649900	0.38626431	0.97907962	-0.514861
O	O114	1	0.02086404	0.49187509	0.61369235	-0.515025
O	O115	1	0.87347066	0.02092834	0.61373628	-0.514973
O	O116	1	0.97908345	0.50815793	0.12649611	-0.514721
O	O117	1	0.49181721	0.61373453	0.02094054	-0.51484
O	O118	1	0.38629492	0.50815898	0.97906529	-0.514611
O	O119	1	0.38629931	0.97913523	0.12644525	-0.515034
O	O120	1	0.12643685	0.50812819	0.38629487	-0.514879
O	O121	1	0.50816830	0.38625372	0.12651055	-0.514657
O	O122	1	0.50815897	0.12641235	0.97912989	-0.514988
O	O123	1	0.97906429	0.12647618	0.38629524	-0.514585
O	O124	1	0.12651168	0.97906415	0.50816279	-0.514797
O	O125	1	0.97912507	0.38630259	0.50815530	-0.51511

O	O126	1	0.49186008	0.87355984	0.61370548	-0.514811
O	O127	1	0.61369753	0.02087997	0.49186071	-0.514736
O	O128	1	0.87355306	0.61373867	0.49184942	-0.514862
O	O129	1	0.61369427	0.87357292	0.02086924	-0.515179
O	O130	1	0.02093135	0.87351604	0.49181405	-0.514857
O	O131	1	0.61374319	0.49185466	0.87347534	-0.514615
O	O132	1	0.61372661	0.87353365	0.49185913	-0.51491
O	O133	1	0.97907058	0.38628723	0.12647988	-0.514477
O	O134	1	0.49185533	0.02088169	0.61373281	-0.515096
O	O135	1	0.12646800	0.50816593	0.97907818	-0.514414
O	O136	1	0.50813978	0.97913863	0.12645199	-0.515152
O	O137	1	0.87350915	0.61373801	0.02091862	-0.514915
O	O138	1	0.97913355	0.50814105	0.38630014	-0.515198
O	O139	1	0.12651313	0.97907955	0.38626050	-0.514757
O	O140	1	0.02091573	0.49184492	0.87349683	-0.514904
O	O141	1	0.50819112	0.38626092	0.97906121	-0.514907
O	O142	1	0.61370894	0.49183340	0.02093697	-0.514581
O	O143	1	0.61369584	0.02086140	0.87355754	-0.514822
O	O144	1	0.87355194	0.49189163	0.61369228	-0.514837
O	O145	1	0.49183917	0.61373442	0.87348628	-0.514471
O	O146	1	0.49183912	0.87358561	0.02086989	-0.515052
O	O147	1	0.02093448	0.87350992	0.61371062	-0.514697
O	O148	1	0.87350074	0.02093106	0.49182554	-0.514854
O	O149	1	0.02086828	0.61370857	0.49183116	-0.514855
O	O150	1	0.50813853	0.12644112	0.38630102	-0.514697
O	O151	1	0.38629628	0.97912036	0.50814479	-0.514884
O	O152	1	0.12645445	0.38627807	0.50813380	-0.51504

```
O  O153  1  0.38630445  0.12642871  0.97912642  -0.515247
O  O154  1  0.97905291  0.12649956  0.50818348  -0.514968
O  O155  1  0.38626314  0.50813398  0.12653003  -0.51461
```

References

(1) Breneman, C. M.; Wiberg, K. B. Determining atom-centered monopoles from molecular electrostatic potentials. The need for high sampling density in formamide conformational analysis. *Journal of Computational Chemistry* **1990**, *11*, 361–373.

(2) Zhao, Y.; Truhlar, D. G. A new local density functional for main-group thermochemistry, transition metal bonding, thermochemical kinetics, and noncovalent interactions. *The Journal of Chemical Physics* **2006**, *125*, 194101.

(3) Weigend, F.; Ahlrichs, R. Balanced basis sets of split valence, triple zeta valence and quadruple zeta valence quality for H to Rn: Design and assessment of accuracy. *Phys. Chem. Chem. Phys.* **2005**, *7*, 3297.

(4) Grimme, S.; Antony, J.; Ehrlich, S.; Krieg, H. A consistent and accurate ab initio parametrization of density functional dispersion correction (DFT-D) for the 94 elements H-Pu. *The Journal of Chemical Physics* **2010**, *132*, 154104.

(5) Perdew, J. P.; Burke, K.; Ernzerhof, M. Generalized Gradient Approximation Made Simple. *Phys. Rev. Lett.* **1996**, *77*, 3865–3868.

(6) Manz, T. A. Introducing DDEC6 atomic population analysis: part 3. Comprehensive method to compute bond orders. *RSC advances* **2017**, *7*, 45552–45581.

(7) Rosen, A. S.; Iyer, S. M.; Ray, D.; Yao, Z.; Aspuru-Guzik, A.; Gagliardi, L.; Notestein, J. M.; Snurr, R. Q. Machine learning the quantum-chemical properties of

metal–organic frameworks for accelerated materials discovery. *Matter* **2021**, *4*, 1578–1597.

(8) Jorgensen, W. L.; Chandrasekhar, J.; Madura, J. D.; Impey, R. W.; Klein, M. L. Comparison of simple potential functions for simulating liquid water. *The Journal of chemical physics* **1983**, *79*, 926–935.

(9) Horn, H. W.; Swope, W. C.; Pitera, J. W.; Madura, J. D.; Dick, T. J.; Hura, G. L.; Head-Gordon, T. Development of an improved four-site water model for biomolecular simulations: TIP4P-Ew. *The Journal of chemical physics* **2004**, *120*, 9665–9678.

(10) Mahoney, M. W.; Jorgensen, W. L. A five-site model for liquid water and the reproduction of the density anomaly by rigid, nonpolarizable potential functions. *The Journal of chemical physics* **2000**, *112*, 8910–8922.

(11) Rick, S. W. A reoptimization of the five-site water potential (TIP5P) for use with Ewald sums. *The Journal of chemical physics* **2004**, *120*, 6085–6093.

(12) Potoff, J. J.; Siepmann, J. I. Vapor–liquid equilibria of mixtures containing alkanes, carbon dioxide, and nitrogen. *AIChE Journal* **2001**, *47*, 1676–1682.

(13) Zhang, L.; Siepmann, J. I. Development of the trappe force field for ammonia. *Collection of Czechoslovak Chemical Communications* **2010**, *75*, 577–591.

(14) Wick, C. D.; Martin, M. G.; Siepmann, J. I. Transferable Potentials for Phase Equilibria. 4. United-Atom Description of Linear and Branched Alkenes and Alkylbenzenes. *The Journal of Physical Chemistry B* **2000**, *104*, 8008–8016.

(15) Chen, B.; Potoff, J. J.; Siepmann, J. I. Monte Carlo Calculations for Alcohols and Their Mixtures with Alkanes. Transferable Potentials for Phase Equilibria. 5. United-Atom Description of Primary, Secondary, and Tertiary Alcohols. *The Journal of Physical Chemistry B* **2001**, *105*, 3093–3104.

(16) Rappe, A. K.; Casewit, C. J.; Colwell, K. S.; Goddard, W. A. I.; Skiff, W. M. UFF, a full periodic table force field for molecular mechanics and molecular dynamics simulations. *Journal of the American Chemical Society* **1992**, *114*, 10024–10035.

(17) Mayo, S. L.; Olafson, B. D.; Goddard, W. A. DREIDING: a generic force field for molecular simulations. *Journal of Physical chemistry* **1990**, *94*, 8897–8909.

(18) Eisenschitz, R.; London, F. Über das Verhältnis der van der Waalsschen Kräfte zu den homöopolaren Bindungskräften. *Zeitschrift für Physik* **1930**, *60*, 491–527.

(19) London, F. Zur theorie und systematik der molekularkräfte. *Zeitschrift für Physik* **1930**, *63*, 245–279.

(20) Li, P.; Merz Jr, K. M. Taking into account the ion-induced dipole interaction in the nonbonded model of ions. *Journal of chemical theory and computation* **2014**, *10*, 289–297.

(21) Hayashi, H.; Watanabe, N.; Udagawa, Y.; Kao, C.-C. The complete optical spectrum of liquid water measured by inelastic x-ray scattering. *Proceedings of the National Academy of Sciences* **2000**, *97*, 6264–6266.

(22) Gold, V., Ed. *Ionization Potentials11We have used throughout the term ionization potential (I.P.) in preference to the more precise ionization energy on grounds of common usage.*; Advances in Physical Organic Chemistry; Academic Press, 1966; Vol. 4; pp 31–71.

(23) Dibeler, V. H.; Walker, J. A. Mass-Spectrometric Study of Photoionization. VI. O2, CO2, COS, and CS2†. *J. Opt. Soc. Am.* **1967**, *57*, 1007–1012.

(24) Yu, D.; Yazaydin, A. O.; Lane, J. R.; Dietzel, P. D.; Snurr, R. Q. A combined experimental and quantum chemical study of CO 2 adsorption in the metal–organic framework CPO-27 with different metals. *Chemical Science* **2013**, *4*, 3544–3556.

(25) Caskey, S. R.; Wong-Foy, A. G.; Matzger, A. J. Dramatic tuning of carbon dioxide uptake via metal substitution in a coordination polymer with cylindrical pores. *Journal of the American Chemical Society* **2008**, *130*, 10870–10871.

(26) Queen, W. L.; Hudson, M. R.; Bloch, E. D.; Mason, J. A.; Gonzalez, M. I.; Lee, J. S.; Gygi, D.; Howe, J. D.; Lee, K.; Darwish, T. A.; others Comprehensive study of carbon dioxide adsorption in the metal–organic frameworks M 2 (dobdc)(M= Mg, Mn, Fe, Co, Ni, Cu, Zn). *Chemical Science* **2014**, *5*, 4569–4581.

(27) Mercado, R.; Vlaisavljevich, B.; Lin, L.-C.; Lee, K.; Lee, Y.; Mason, J. A.; Xiao, D. J.; Gonzalez, M. I.; Kapelewski, M. T.; Neaton, J. B.; others Force field development from periodic density functional theory calculations for gas separation applications using metal–organic frameworks. *The Journal of Physical Chemistry C* **2016**, *120*, 12590–12604.

(28) Haldoupis, E.; Borycz, J.; Shi, H.; Vogiatzis, K. D.; Bai, P.; Queen, W. L.; Gagliardi, L.; Siepmann, J. I. Ab initio derived force fields for predicting CO2 adsorption and accessibility of metal sites in the metal–organic frameworks M-MOF-74 (M= Mn, Co, Ni, Cu). *The Journal of Physical Chemistry C* **2015**, *119*, 16058–16071.

(29) Märcz, M.; Johnsen, R. E.; Dietzel, P. D.; Fjellvåg, H. The iron member of the CPO-27 coordination polymer series: Synthesis, characterization, and intriguing redox properties. *Microporous and mesoporous materials* **2012**, *157*, 62–74.

(30) Dietzel, P. D.; Besikiotis, V.; Blom, R. Application of metal–organic frameworks with coordinatively unsaturated metal sites in storage and separation of methane and carbon dioxide. *Journal of Materials Chemistry* **2009**, *19*, 7362–7370.

(31) Tayfuroglu, O.; Keskin, S. Modeling CO2 Adsorption in Flexible MOFs with Open Metal Sites via Fragment-Based Neural Network Potentials. **2025**,

(32) Goeminne, R.; Vanduyfhuys, L.; Van Speybroeck, V.; Verstraelen, T. DFT-Quality adsorption simulations in metal–organic frameworks enabled by machine learning Potentials. *Journal of Chemical Theory and Computation* **2023**, *19*, 6313–6325.

(33) Hou, X.-J.; He, P.; Li, H.; Wang, X. Understanding the adsorption mechanism of C2H2, CO2, and CH4 in isostructural metal–organic frameworks with coordinatively unsaturated metal sites. *The Journal of Physical Chemistry C* **2013**, *117*, 2824–2834.

(34) Herm, Z. R.; Swisher, J. A.; Smit, B.; Krishna, R.; Long, J. R. Metal- organic frameworks as adsorbents for hydrogen purification and precombustion carbon dioxide capture. *Journal of the American Chemical Society* **2011**, *133*, 5664–5667.

(35) Dzubak, A. L.; Lin, L.-C.; Kim, J.; Swisher, J. A.; Poloni, R.; Maximoff, S. N.; Smit, B.; Gagliardi, L. Ab initio carbon capture in open-site metal–organic frameworks. *Nature chemistry* **2012**, *4*, 810–816.

(36) Glover, T. G.; Peterson, G. W.; Schindler, B. J.; Britt, D.; Yaghi, O. MOF-74 building unit has a direct impact on toxic gas adsorption. *Chemical Engineering Science* **2011**, *66*, 163–170.

(37) Schoenecker, P. M.; Carson, C. G.; Jasuja, H.; Flemming, C. J.; Walton, K. S. Effect of water adsorption on retention of structure and surface area of metal–organic frameworks. *Industrial & Engineering Chemistry Research* **2012**, *51*, 6513–6519.

(38) Yang, D.-A.; Cho, H.-Y.; Kim, J.; Yang, S.-T.; Ahn, W.-S. CO 2 capture and conversion using Mg-MOF-74 prepared by a sonochemical method. *Energy & Environmental Science* **2012**, *5*, 6465–6473.

(39) Carné-Sánchez, A.; Stylianou, K. C.; Carbonell, C.; Naderi, M.; Imaz, I.; Maspoch, D. Protecting metal–organic framework crystals from hydrolytic degradation by spray-dry encapsulating them into polystyrene microspheres. *Advanced Materials* **2015**, *27*, 869–873.

(40) Liang, Z.; Marshall, M.; Chaffee, A. L. Comparison of Cu-BTC and zeolite 13X for adsorbent based CO2 separation. *Energy Procedia* **2009**, *1*, 1265–1271.

(41) Millward, A. R.; Yaghi, O. M. Metal- organic frameworks with exceptionally high capacity for storage of carbon dioxide at room temperature. *Journal of the American Chemical Society* **2005**, *127*, 17998–17999.

(42) Rios, R. B.; Correia, L. S.; Bastos-Neto, M.; Torres, A. E. B.; Hatimondi, S. A.; Ribeiro, A. M.; Rodrigues, A. E.; Cavalcante Jr, C. L.; de Azevedo, D. C. Evaluation of carbon dioxide–nitrogen separation through fixed bed measurements and simulations. *Adsorption* **2014**, *20*, 945–957.

(43) Yazaydın, A. O.; Benin, A. I.; Faheem, S. A.; Jakubczak, P.; Low, J. J.; Willis, R. R.; Snurr, R. Q. Enhanced CO2 adsorption in metal-organic frameworks via occupation of open-metal sites by coordinated water molecules. *Chemistry of Materials* **2009**, *21*, 1425–1430.

(44) Rother, J.; Fieback, T. Multicomponent adsorption measurements on activated carbon, zeolite molecular sieve and metal–organic framework. *Adsorption* **2013**, *19*, 1065–1074.
